# Effects of Isohexide Stereochemistry on Vinylogous Urethane Covalent Adaptable Networks

**DOI:** 10.1021/acs.macromol.5c00270

**Published:** 2025-09-29

**Authors:** Noé Fanjul-Mosteirín, Karin Odelius

**Affiliations:** Department of Fibre and Polymer Technology, 7655 KTH Royal Institute of Technology, SE-100 44 Stockholm, Sweden

## Abstract

The starch-derived isohexides, with their unique structures of two fused tetrahydrofuran rings in a *cis* conformation, have been exploited to prepare covalent adaptable networks (CANs) and to tailor and understand their structure–property relationships, in pursuit of replacing oil-based thermosets. Here, dynamicity was achieved through vinylogous urethane chemistry, rigidity via the use of the starch-derived isomeric building blocks isosorbide, isomannide, and isoidide, and flexibility through the amines utilized. Similar to what is known for thermoplastics, depending on the isomer chosen, thermal stability and mechanical properties could be tailored to some extent. The distance between cross-links was ruled by the amines employed, and when this distance was long enough to allow sufficient chain mobility, stereochemical effects on mechanical performance were observed. The CAN structures all display thermoset properties, and as a consequence of the incorporated dynamic bonds, they were mechanically reprocessable. Based on the CANs structural design, i.e., isohexide isomer and amine structure, tensile strengths (σ_b_) ranging from 1.57 to 19.1 MPa, glass transition temperatures (*T*_g_) ranging from 20 to 114 °C, and thermal stabilities (*T*_d,5%_) between 200 and 305 °C were achievable. Mechanical reprocessing was proven, and no mechanical performance decay was observed after two reprocessing cycles. This provides important information on the structure–property relationship of CANs from starch-derived building blocks, and consequently, how material properties can be tailored depending on the targeted application.

## Graphical abstract

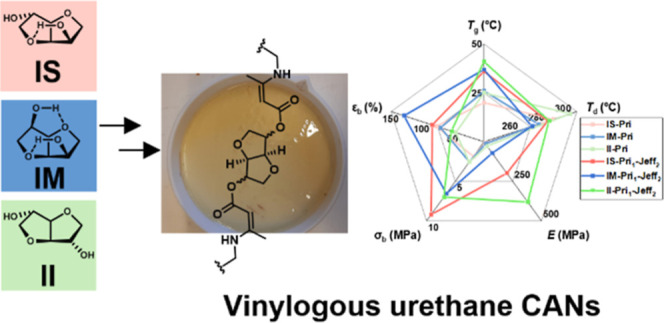

## Introduction

Isosorbide, being one of the isohexide isomers and a biobased platform chemical originating from starch, is of large interest as a biobased building block in plastics.^1^ Thanks to the two *cis*-fused tetrahydrofuran rings in the isohexide having a V-shape, rigidity and toughness are conferred to isohexide-based polymers.^2–6^ The different configurations of the isohexide stereoisomers render them different reactivities, with isomannide (*endo-endo*) being the least reactive due to the steric hindrance of its hydroxyl groups, and as a consequence, polymers obtained by polycondensation reactions based on isomannide tend to have the lowest molecular weights.^7^ Isoidide with an *exo-exo* conformation and therefore the least sterically hindered isomer is also the most reactive, but unfortunately, this stereoisomer is the least commercially available since its precursor, L-iditol, is a rather rare sugar derivative.^8^ Isosorbide is obtained from dehydration of D-sorbitol and is also the most available and exploited one, with a reactivity between the other two isomers. Isosorbide has its hydroxyl group at the C2 position in the *exo* conformation, whereas the hydroxyl group at the C5 position is in the *endo* conformation.^9^

In addition to different reactivities conferred by the stereochemistry of isohexide, the stereochemistry has also been proven to impart changes in the physical properties for thermoplastics containing a combination of urethane, ester, and thioether structures in their backbone.^2^ As an example, the isomers isomannide and isoidide, with opposite optical configurations and reactivity, were used in the synthesis of linear hybrid poly­(urethane-thioether)­s.^10^ As a consequence of the different stereochemistries, the thermomechanical properties were vastly different, with the isoidide-based polymer being semicrystalline with a *T*_g_ of 15 °C and a *T*_m_ ranging from 111 to 158 °C and the isomannide-based polymer being completely amorphous with a *T*_g_ of 15 °C. These thermal features consequently had a large impact on the material’s mechanical properties, where the semicrystalline isoidide-based polymers displayed plastic properties, with lower elongations at break and higher strength at break compared to the elastomeric properties, observed for the amorphous isomannide-based polymers.

Covalent adaptable networks, CANs, are a class of thermosets that bear reversible covalent bonds, which, under certain stimuli such as heat or light, can perform bond exchange that ultimately allows the thermoset to be recycled and reprocessed. This feature represents a very promising approach to bridge the gap between thermosets and thermoplastics.^11–18^ Among the different dynamic reactions involved in the development of covalent adaptable networks (CANs), we can find imine exchange,^19,20^ boronic ester metathesis,^21,22^ disulfide exchange,^23,24^ transesterification,^25,26^ transcarbamoylation reactions,^27,28^ and vinylogous urethane exchanges.^29,30^ The vinylogous urethane moieties are formed when an acetoacetate reacts with a primary amine, with an equivalent of water formed as a byproduct.^31,32^ This type of reversible chemistry has been exploited in the synthesis of various CAN systems ranging from linear poly­(acrylate/methacrylate)-bearing pendant acetoacetate moieties that subsequently were cross-linked^33,34^ to a more classical thermoset from low-molar-mass molecules, where at least one of the monomers bears either a multifunctional (<2) amine or acetoacetate.^35,36^ Isosorbide-containing CANs have for example been designed utilizing radical polymerization to form copolymers of 2-levunoyl-5-methacrylate-isosorbide and methyl acrylate, rendering a large set of statistical copolymers with a broad comonomer composition. After cross-linking the copolymers with dynamic acylhydrazone bonds, the formed CANs were compared to their linear thermoplastic counterparts and shown to portray *T*_g_s increased from 115 to 170 °C and thermal stabilities (*T*_d,5%_) enhanced by 10 to 80 °C depending on the formulation. The CANs were also chemically recycled by treating them under acidic aqueous conditions with acetone that ultimately acted as a scavenging agent for the free amines to be released.^37^ Isosorbide has also been utilized to form vinylogous urethane CANs for which acetoacetate reacted with triethylenetetramine and amines of various lengths. The linear prepolymers were subsequently cross-linked utilizing a vanillic acid bis-epoxide monomer. The thermomechanical properties of the isosorbide-containing CANs were ruled by the length of the amines employed, and a *T*_g_ range of 36–57 °C, a tensile strength range of 31.5–114.6 MPa, and an elongation at break range of 9.5–150% were confirmed.^38^

In the present work, we aim to use the intrinsic strong and rigid fused furan rings present in the biobased isohexide isomers to prepare a series of renewable vinylogous urethane CANs with tailorable and stereo-influenced thermomechanical properties. For this purpose, the hydroxyl groups on isohexides were converted into acetoacetate monomers, which subsequently were reacted with amines of different lengths and functionalities, enabling the tuning of the thermomechanical properties. We believed that the distance between cross-links is critical, as the longer this distance is, the lower the *T*_g_ and the higher the chain mobility would be, and consequently, the more likely stereoisomer effects of the isohexides would uncover.

## Experimental Section

### Materials

All chemicals were used as received without further purification unless otherwise indicated. They include isosorbide (TCI Europe, ≥98.0%), isomannide (Combi blocks, 97%), 2,2,6-trimethyl-4*H*-1,3-dioxin-2-one (TDMO) distilled under high vacuum at 54 °C and stored under N_2_ (TCI Europe, 95%), tosyl chloride (Sigma-Aldrich, ≥99%), triethylamine (Sigma-Aldrich, ≥99%), potassium acetate (Sigma-Aldrich, ≥99%), tris­(2-aminoethyl)­amine (TREN) (Sigma-Aldrich, 96%), para-toluene sulfonic acid monohydrate (Sigma-Aldrich, ≥99%), *N*,*N*-4-dimethylaminopyridine (DMAP) (Sigma-Aldrich, ≥99%), 1,4-butanediol (Riedel-de Haën, 99%), trimethylolpropane tris­[poly­(propylene glycol), amine terminated] ether (Jeff) (Sigma-Aldrich, average *M*_n_ ≈ 440 g/mol), Priamine 1071 (Pri) (kindly supplied by Croda), benzylamine (Sigma-Aldrich, 99%), hexylamine (Sigma-Aldrich, ≥99%), xylene (mixture of isomers) (VWR, ≥98%), dichloromethane (Sigma-Aldrich, ≥99%), *N*-methyl-2-pyrrolidone (Sigma-Aldrich, ≥99%), and methanol (anhydrous) (Sigma-Aldrich, 99.8%).

### Characterization

NMR spectra were recorded on a Bruker Avance 400 MHz spectrometer. Chemical shifts are reported in parts per million (ppm) and referenced to the residual solvent signal (CDCl_3_: ^1^H, δ = 7.26 ppm, ^13^C, δ = 77.2 ppm, DMSO: ^1^H, δ = 2.50 ppm, ^13^C, δ = 39.5 ppm, TMS: ^1^H, δ = 0.00 ppm). Multiplicities are reported as s = singlet, brs = broad singlet, d = doublet, t = triplet, and m = multiplet. Multiplicity is followed by integration and coupling constant (*J*) in Hz.

FTIR spectra were recorded on a PerkinElmer Spectrum 2000 instrument equipped with a single reflection attenuated total reflectance accessory. 8 to 16 scans were recorded with a 4 cm^–1^ resolution, between 4000 and 600 cm^–1^.

Dynamic mechanical analysis (DMA) of the covalent adaptable networks (CANs) was measured with a Q800 dynamic mechanical analyzer (TA Instruments) in tension mode. Rectangular-shaped specimens were taken from hot-pressed samples. Determination of *T*_g_ was carried out in triplicate with a 3 °C min^–1^ heating rate and 0.1% strain at 1 Hz. Stress relaxation experiments were carried out with a 2% strain rate for 20 min. Creep experiments were carried out by applying a stress of 0.01 MPa for 20 min. Cross-linking density (υ_e_) was calculated using eq 1, where *E*′ is the storage modulus at the rubbery plateau at the respective temperature *T* and *R* is the universal gas constant (8.314 J K^–1^ mol^–1^). DMA data were analyzed by TA Universal Analysis Software (v. 4.5).

1 $$\upsilon_{e}=\frac{E^{\prime }}{3RT}$$

DSC analysis was performed on a Mettler Toledo DSC 1 instrument with the samples (5–10 mg) sealed in Al crucibles. The samples were subjected to a heating–cooling–heating–cooling cycle with a 10 °C min^–1^ temperature ramp and under a N_2_ atmosphere with a 50 mL min^–1^ flow. The temperature range was from −10 °C to 140 °C. The glass transition temperature (*T*_g_) was taken from the second heating scan as the midpoint of the *T*_g_.

Compression molding of the CANs was carried out on a TP 400 hot-press (Fontijne Presses BV). Each CAN was first ground with a coffee grinder and then dispersed in a steel mold with the appropriate shape (dumbbell or strip). The mold was sandwiched between two stainless steel disks (Ø = 23 cm, *T* = 0.5 cm) covered with thin PTFE sheets (*T* = 0.1 mm). The samples were pressed at 140 °C for 20 min with several (at least three) venting cycles.

The gel content (GC) of the synthesized CANs was calculated by the following equation:

2 $$GC=\frac{m_{i}{-m}_{f}}{m_{i}}\times 100$$

A piece of each CAN with an initial mass *m*_i_ was immersed in the corresponding solvent for 24 h. Afterward, the solvent was decanted off, and the CAN was dried under reduced pressure until constant mass *m*_f_ was reached. GC % is given as the average value with its corresponding standard deviation of three different measurements for every single CAN.

Tensile testing was performed on an Instron 5944 tensile tester. The CANs were tested using dumbbell-shaped specimens (38 mm (*L*) × 5 mm (*W*) × 0.8 mm (*T*), effective gauge length of 22 mm) prepared by hot-pressing the grinded CANs in a custom-made steel mold with the same dimensions. The cross-head speed was set to 0.05 mm·min^–1^. CANs were conditioned at 23 ± 2 °C and 50% relative humidity for 24 h prior to testing.

The thermal stability of the synthesized CANs was evaluated by a Mettler Toledo TGA/DSC 851e module instrument. An inert flow (nitrogen) of 50 mL min^-1^ and a heating rate of 5 °C per minute were utilized. The temperature scan was performed from 25 to 650 °C. The onset temperatures (*T*_onset_) at 5 wt % mass loss were determined.

### Synthesis of Isohexide Acetoacetate and 1,4-Butanediol Acetoacetate

In a 50 mL round-bottomed flask, isosorbide, isomannide, isoidide (prepared according to Schemes S1–S3 and the experimental procedure given in Supporting Information) (5 g, 34.21 mmol, 1 equiv) or 1,4-butanediol (3.13 mL, 34.21 mmol, 1 equiv), freshly distilled 2,2,6-trimethyl-4*H*-1,3-dioxin-4-one (TMDO) (9.54 mL, 71.85 mmol, 2.1 equiv), and xylene (6.2 mL) were mixed, and a still head with a thermometer and a collecting round-bottomed flask was attached and heated up to 135 °C. The temperature of the still head was at 54 °C, revealing that acetone was being distilled off from the reaction media. After 90 min, the still head temperature dropped and the reaction was distilled off at 60 °C under high vacuum in order to remove the excess of TMDO and the remaining acetone and xylene. Depending on the isomer used, either a pale-yellow oil (isomannide acetoacetate (IM-AAc) (10.32 g, 32.84 mmol, 96% yield), a solid (isosorbide acetoacetate (IS-AAc) (10.52 g, 33.53 mmol, 98% yield), or isoidide acetoacetate (II-AAc) (10.53 g, 33.53 mmol, 98% yield) was obtained. Characterization data was in agreement with those reported in the literature.^38^ An orangish oil was obtained (1,4-butanediol acetoacetate (1,4-BD-AAc) (8.64 g, 33.45 mmol, 98% yield)). Characterization data was in agreement with those reported in the literature.^39^ IS-AAc, ^**1**^ **H NMR** (400 MHz, CDCl_3_) δ 5.24–5.23 (m, 1H, C_(1)_H–O–C=O), 5.21–5.17 (m, 1H, C_(5)_H–O–C=O), 4.83 (t, 1H, C*H*–C_(5)_H), 4.52–4.48 (m, 1H, C*H*–C_(1)_H), 4.03–3.87 (m, 3H, C*H*_anti_–C_(5)_H–CH–C*H*_2_), 3.82 (dd, 1H, ^3^ *J*_H–H_ = 10.1 and 4.9 Hz, C*H*_syn_–C_(5)_H), 3.50 (s, 2H, C_(5)_H–O–C­(=O)–C*H*_2_), 3.47 (s, 2H, C_(1)_H–O–C­(=O)–C*H*_2_), 2.27 (s, 3H, C*H*_3_–C­(=O)–CH_2_–C­(=O)–O–CH_syn_–CH), 2.23 (s, 3H, C*H*_3_–C­(=O)–CH_2_–C­(=O)–O–CH_anti_–CH). ^**13**^ **C APT NMR** (100 MHz, CDCl_3_) δ 200.1 (CH_3_–*C*=O), 166.5 (C_(1)_H–O–*C*=O), 166.3 (C_(5)_H–O–*C*=O), 85.9 (*C*H–C_(1)_H–O–C=O), 80.8 (*C*H–C_(5)_H–O–C=O), 78.7 (*C*_(5)_H–O–C=O), 74.9 (*C*_(1)_H–O–C=O), 73.2 (*C*H_2_–C_(1)_H–O–C=O), 70.5 (*C*H_2_–C_(5)_H–O–C=O), 49.9 (*C*H_2_–C=O), 30.4 (*C*H_3_–C­(=O)–CH_2_–C­(=O)–O–CH_syn_–CH), 30.2 (*C*H_3_–C­(=O)–CH_2_–C­(=O)–O–CH_anti_–CH).

IM-AAc, ^**1**^ **H NMR** (400 MHz, CDCl_3_): δ 5.19–5.15 (m, 2H, CH–O–C=O), 4.77–4.67 (m, 2H, C*H*–CH–O–C=O), 4.04 (dd, 2H, ^3^ *J*_H–H_ = 9.5 and 6.4 Hz, C*H*_anti_–CH–O–C=O), 3.79 (dd, 2H, ^3^ *J*_H–H_ = 9.5 and 6.7 Hz, C*H*_syn_–CH–O–C=O), 3.51 (s, 4H, CH_2_–C=O), 2.28 (s, 6H, CH_3_). ^**13**^ **C APT NMR** (100 MHz, CDCl_3_): δ 200.0 (CH_3_–*C*=O), 166.6 (O–C=O), 80.4 (*C*H–O–C=O), 74.5 (*C*H–CH–O–C=O), 70.5 (*C*H_2_–CH–O–C=O), 49.9 (*C*H_2_–C=O), 30.2 (CH_3_).

II-AAc ^**1**^ **H NMR** (400 MHz, CDCl_3_): δ 5.26–5.25 (m, 2H, CH–O–C=O), 4.64 (s, 2H, C*H*–CH–O–C=O), 4.00–3.96 (m, 4H, C*H*_anti_–CH–O–C­(=O)–C*H*), 3.79 (m, 4H, C*H*_syn_–CH–O–C­(=O)–C*H*), 3.51 (s, 4H, CH_2_–C=O), 2.26 (s, 6H, CH_3_). ^**13**^ **C APT NMR** (100 MHz, CDCl_3_): δ 200.0 (CH_3_–*C*=O), 166.2 (O–C=O), 85.3 (*C*H–O–C=O), 78.4 (*C*H–CH–O–C=O), 72.5 (*C*H_2_–CH–O–C=O), 49.9 (*C*H_2_–C=O), 30.4 (CH_3_).

1,4-BD-AAc ^**1**^ **H NMR** (400 MHz, CDCl_3_): δ 4.18–4.15 (m, 4H, CH_2_–O), 3.46 (s, 4H, CH_2_–C=O), 2.27 (s, 6H, CH_3_), 1.75–1.72 (m, 4H, C*H*_2_–C*H*_2_–CH_2_–O). ^**13**^ **C APT NMR** (100 MHz, CDCl_3_): δ 200.6 (CH_3_–*C*=O), 167.2 (O–C=O), 64.9 (CH_2_–O), 50.2 (*C*H_2_–C=O), 30.3 (CH_3_), 25.2 (*C*H_2_–*C*H_2_–CH_2_–O).

### Synthesis of IH-Pri_*x*_-Jeff_*y*_ and BD-Pri_1_-Jeff_2_

To a solution of Priamine 1071 (0–1 equiv) and Jeff (0.85–0 equiv) in CHCl_3_ (2 mL), a solution of monomer isohexide acetoacetate (IH-AAc) (2 g, 6.37 mmol, 1 equiv) was added. The mixture was stirred in a vortex for 1 min and then poured in a Teflon mold (10 cm diameter). The solvent was left to be evaporated at room temperature overnight, and a film was obtained. The obtained film IH-Pri_*x*_-Jeff_*y*_ was cured in an oven at 140 °C for 8 h.

To a solution of Priamine 1071 (1.30 g, 2.10 mmol, 0.33 equiv) and Jeff (1.88 mL, 4.20 mmol, 0.66 equiv) in CHCl_3_ (2 mL), a solution of monomer 1,4-BD-AAc (1.64 g, 6.37 mmol, 1 equiv) was added. The mixture was stirred in a vortex for 1 min and then poured in a Teflon mold (10 cm diameter). The solvent was left to be evaporated at room temperature overnight, and a film was obtained. The obtained film BD-Pri_*x*_-Jeff_*y*_ was cured in an oven at 140 °C for 8 h.

## Results and Discussion

In pursuit of incorporating a rigid core and a high amount of renewable carbon content in the synthesis of vinylogous urethane CANs, isohexide diacetoacetates (IH-AAc) with beta keto ester moieties were prepared and allowed to react with amines, rendering the corresponding vinylogous urethane functionalization and reversible properties. The prepared CANs were designed to allow both thermal and mechanical properties to be tailored by the amine structure, which provides variable distances between cross-links and enables stereochemical effects on the mechanical properties of the CANs.

IH-AAc were obtained at high yields via coupling the IHs with 2,2,6-trimethyl-4*H*-1,3-dioxin-2-one (TDMO) (Schemes S1–S3). Upon heating, a *retro*-Diels–Alder reaction takes place, which releases acetylketene and acetone,^40^ where the acetylketene reacts with the diols of the corresponding IHs yielding the desired IH-AAc, and the formed acetone is removed by distillation. For the commercially available IHs, i.e., isosorbide (IS) and isomannide (IM), that render the corresponding IS-AAc and IM-AAc, isolated yields of 98 and 96%, respectively, were achieved. For isoiodide (II), the least available isomer, a three-step synthetic methodology involving inversion of the optical configuration of IM was employed, followed by acetyl acetylation yielding II-AAc (98% yield), reaching an overall yield of 64% (Scheme S3).^10^ Prior to the synthesis of CANs, we confirmed that no aminolysis of the ester bond occurred as a side reaction during the formation of the vinylogous urethane moiety. A model system was set up, where IH-AAc (157 mg, 0.5 mmol, 1 equiv) and the primary amine hexylamine (145 μL, 1.1 mmol, 2.2 equiv) in CHCl_3_ (0.5 mL) were allowed to react at room temperature for 4 h. To our delight, the analysis of the crude material by ^1^H NMR revealed full conversion of the starting material and only the formation of the corresponding vinylogous urethane adduct (Figures S1 and S2). For II-AAc, upon addition of hexylamine, a solid was immediately precipitated, revealing the fast kinetics for this particular example, where the solid was insoluble, hampering further characterization. Additionally, to elucidate whether the effects we see on material properties (vide infra) are related to the reactivity of IH-AAc or the stereoregularity of the produced materials, a model reaction with monoamines was conducted. First, IS-AAc and IM-AAc were treated with hexylamine, rendering the corresponding hexyl-based vinylogous urethane, i.e., IS-Hx-VU and IM-Hx-VU (Scheme S4). These compounds were then treated with a large excess of benzyl amine (Bn-NH_2_) (10 equiv) in DMSO-*d*_6_ ([IH-Hx-VU] = 100 mM), and the mixtures were heated to 80, 100, and 120 °C (Scheme S5), respectively. ^1^H NMR spectra were recorded at predefined time intervals, and the VU exchange was followed by integration of the NH–C*H*_2_ signals (Figures S17–S22). The remaining fractions of IH-Hx-VU were plotted against time, and it was possible to calculate the rate constant (k), considering that at low conversions, the reaction occurred under pseudo-first-order conditions as a large excess of Bn-NH_2_ was present in the reaction media (Figures S23–S25). We subsequently calculated the activation energy (*E*_a_) using the Arrhenius law (Figure S26). The *E*_a_ values determined were 40.7 and 38.2 kJ mol^–1^, respectively, for IS-Hx-VU and IM-Hx-VU, revealing that stereochemistry does not have a significant impact on the reactivity of the VU.

IH-derived vinylogous urethane CANs were prepared by combining the three different diacetoacetates (IS-AAc, IM-AAc, and II-AAc) with three different polyamines (TREN, Priamine 1071 (Pri), and trimethylolpropane tris­[poly­(propylene glycol), amine terminated] ether (Jeff)) at different ratios via film casting at room temperature from CHCl_3_, followed by thermal curing at 140 °C for 8 h (Scheme 1). Attempts to conduct the synthesis of CANs in bulk were unsuccessful, as the high reactivity and thereby immediate precipitation of the CAN after mixing the starting materials rendered a heterogeneous thermoset. Thereby, a battery of 13 CANs was prepared. The CANs are named IH-Amine­(a)_*x*_-Amine­(b)_*y*_, where IH refers to the stereoisomer(s) employed and x and y to the molar ratio of amine a to amine b. The amines ranged from short and rigid, TREN, to long and flexible, Pri, with Jeff, the polyethylene glycol (PEG)-based triamine, as an intermediate and were used to prepare a series of CANs with tailorable thermo-mechanical properties (Table 1).

**Scheme 1:**
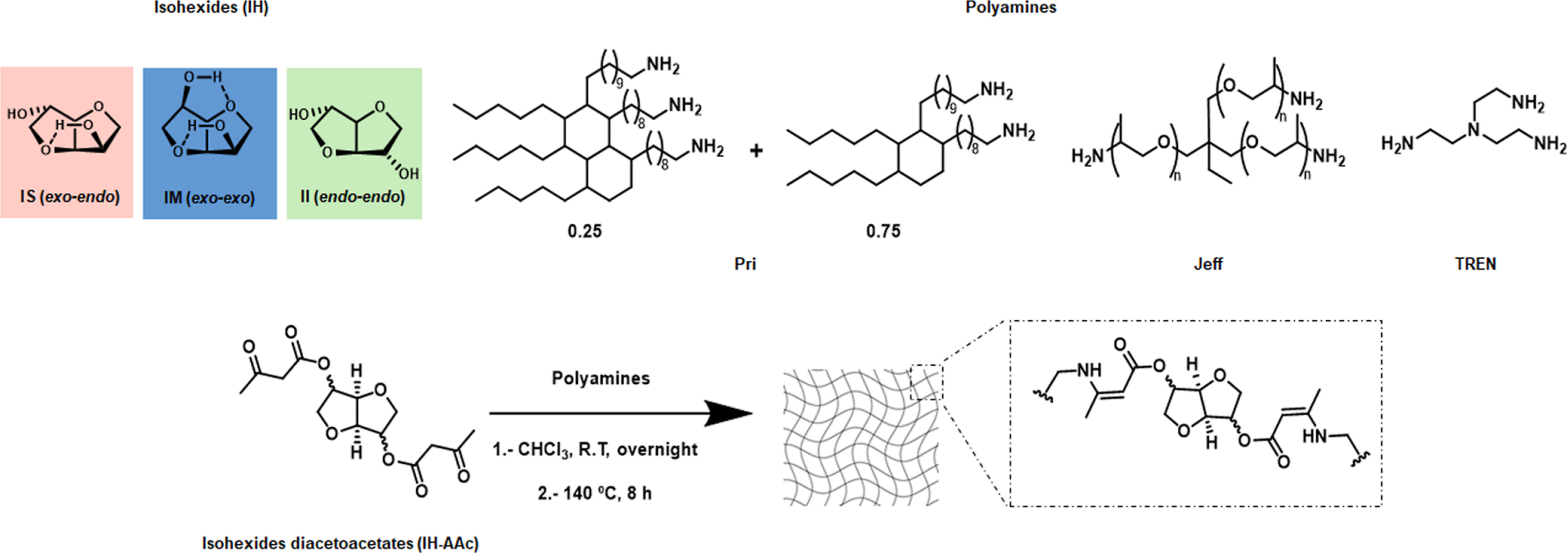
Synthesis of Vinylogous Urethane CANs via Film Casting of IS-AAc, IM-AAc, and II-AAc with Polyamines Priamine 1071 (Pri), Jeffamine T-403 (Jeff), and TREN

**Table 1:** Thermal and Viscoelastic Properties of IH-Based Vinylogous CANs

entry	CAN	*T*_g_ (°C)^a^	*E*_a_ (kJ/mol)^b^	*T*_v_ (°C)^b^	*É* (MPa)^c^	υ_e_ (mol m^–3^)^d^	*T*_d,5%_ (°C)
1	IS-Pri	20 ± 1	57.3	–12	1.15 ± 0.20	123 ± 22	292
2	IM-Pri	26 ± 3	44.1	–38	1.44 ± 0.50	155 ± 54	281
3	II-Pri	25 ± 4	64.7	2	1.43 ± 0.20	154 ± 22	305
4	II_0.5_-IM_0.5_-Pri	28 ± 4	45.2	–33	1.91 ± 0.46	206 ± 49	290
5	IS-Pri_1_-Jeff_2_	36 ± 2	49.0	–18	2.05 ± 0.21	221 ± 22	288
6	IM-Pri_1_-Jeff_2_	37 ± 1	53.4	–14	1.71 ± 0.26	184 ± 28	276
7	II-Pri_1_-Jeff_2_	41 ± 1	53.0	–2	1.45 ± 0.15	156 ± 16	289
8	II_0.5_-IM_0.5_-Pri_1_-Jeff_2_	44 ± 2	54.2	3	2.21 ± 0.12	238 ± 13	282
9	IS-Jeff	61 ± 1	57.7	31	3.26 ± 0.29	350 ± 31	280
10	IM-Jeff	63 ± 1	57.9	29	2.50 ± 0.34	269 ± 36	269
11	II-Jeff	59 ± 1	70.6	51	3.17 ± 0.48	341 ± 52	295
12	II_0.5_-IM_0.5_-Jeff	63 ± 3	55.3	28	3.21 ± 0.31	345 ± 34	272
13	IS-TREN	114 ± 1	n.d	n.d	n.d	n.d	200

a Obtained by triplicate DMA measurements.

b Obtained from strain–stress relaxation curves fitted to the Arrhenius law and adjusted to a Maxwell model.

c Storage modulus in the rubbery plateau (*T* = 100 °C).

d Calculated using the equation $\upsilon_{e}=\frac{E^{\prime }}{3RT}$ .

To confirm the formation of the desired vinylogous urethane CANs, Fourier transform infrared (FTIR) spectroscopy was used. Both ester (1742 and 1741 cm^–1^) and ketone (1712, 1711, and 1707 cm^–1^) bands of IH-AAc (Figures S27–S29) disappeared, and the corresponding new formed vinylogous urethane moiety displayed new bands at lower frequencies (1654–1645 and 1605–1590 cm^–1^) which correspond to the carbonyl ester (C=O) stretching and vinyl (C=C) stretching, respectively (Figures S31–S43). GC experiments to prove cross-linking in the synthesized CANs were performed by immersing the CAN in different solvents (Figure S45). Here, the GC ranged from 84 to 99% (Figure 1 and Table S1) in protic polar solvents like EtOH and medium to high polar aprotic solvents like CHCl_3_, THF, and DMF. When immersed at pH = 7 and under basic conditions such as NaOH (1 M), the CANs exhibited very high stability toward alkali hydrolysis. The highly hydrophobic nature of the CANs, especially those containing Pri, explains the high GC observed under basic and neutral aqueous media. The CANs displayed a thermal stability range of *T*_d,5%_ = 270–305 °C, as determined by TGA (Figure S46), values higher than the monomeric counterparts, exhibiting *T*_d,5%_ = 221–230 °C. IS-TREN was an exception (*T*_d,5%_ = 200), which had a very low thermal stability as a consequence of the high cross-linking density, due to the short and very rigid amine like TREN yielding CANs with a higher N/C ratio.^41^ Overall, two trends were observed originating from the nature of the IH isomeric structure and the ratios and structures of the amines used. The processes linked to thermal degradation phenomena in IH-based systems are a result to β-elimination and subsequent enol–ether reactions occurring at high temperatures.^42,43^ The II-based CANs with the *exo-exo* configuration resulted to be the most stable CANs regardless of the amine employed (Table 1, entries 3, 7, and 11), therefore the degradation process is less prone to happen in the CANs bearing II building blocks. When mixtures of opposite chiral centers, II and IM, were combined, an enhancement of the thermal stability was observed compared to the less stable IM-based CANs, whereas a decrease of the thermal stability was observed compared to the more stable II-based CANs for all the amines employed. This behavior can be attributed to the lower content of less stable IM within the synthesized CANs. With regard to the IS-based CANs, an expected stability in between the less stable IM-based and the more stable II-based CANs was seen, due to the presence of the *endo-exo* conformation of its hydroxyl groups (Table 1, entries 1, 5, and 9).

**Figure 1:**
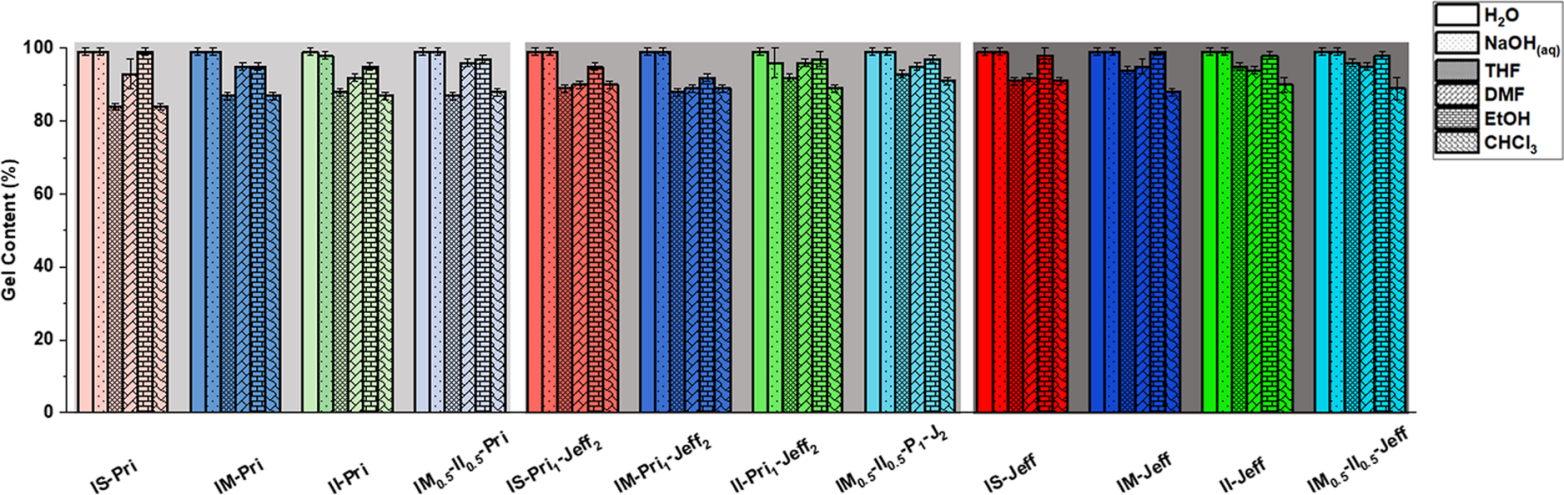
GC of IH-based vinylogous CANs.

The thermal transitions, viscoelastic behavior, and stress relaxation behavior of the obtained vinylogous urethane CANs were analyzed by DMA. The evolution of storage (*E*′) and loss (*E*″) moduli was monitored between 0 and 140 °C, and the *T*_g_ values were determined from the maxima value of the tan δ curves (Figure 2). The presence of different IH stereoisomers in the CAN backbone did not result in a significant difference in *T*_g_ regardless of the amine or the combination of amines employed, as expected. II-based linear polymers have been reported to be semicrystalline as a consequence of its symmetric nature and the *exo-exo* configuration.^43,44^ However, in this study, DSC analysis did not show any crystallinity for II-Pri_*x*_-Jeff_*y*_, likely due to the cross-linked structure of the CANs, confirming that all CANs were amorphous (Figure S47). It was possible to tailor *T*_g_ values from 20 to 114 °C by varying the nature and ratios of the amines employed. The fully Pri-based CANs exhibited low *T*_g_ (20–28 °C) values (Table 1, entries 1–4) as a consequence of long distances between the cross-linking points. The incorporation of a shorter and more rigid triamine like Jeff with a 2:1 ratio compared to Pri resulted in an increase of *T*_g_ (36–44 °C) (Table 1, entries 5–8), as the distance between cross–linking points is shorter. Full substitution of long and flexible Pri for shorter and more rigid Jeff rendered CANs with higher *T*_g_ (59–63 °C) (Table 1, entries 9–12). When the shortest and most rigid amine was employed, i.e., TREN, the highest *T*_g_ (114 °C) (Table 1, entry 13) was observed.

**Figure 2:**
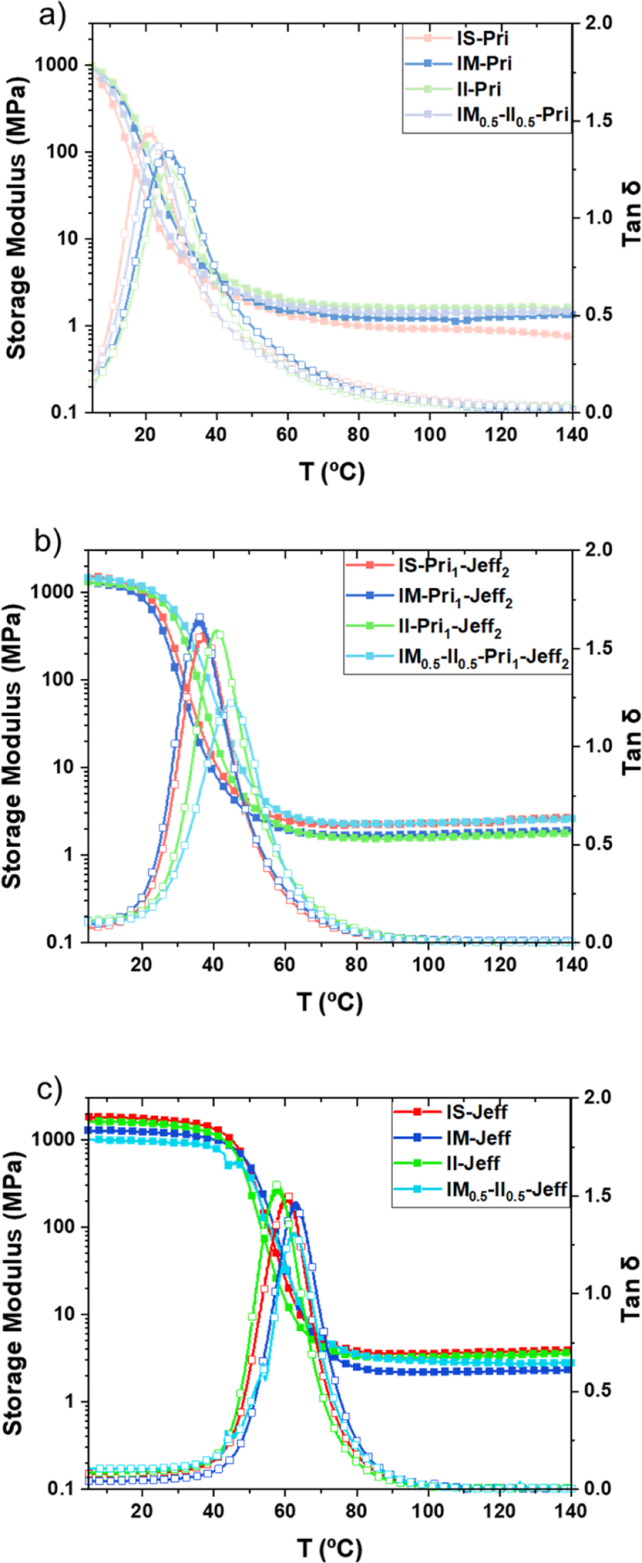
DMA curves of CANs IH-Pri (a), IH-Pri_1_-Jeff_2_ (b), and IH-Jeff (c).

The cross-link density (υ_e_), calculated from the storage moduli (*E*′) in the rubbery plateau, was higher when short and rigid Jeff was used in the CAN formulation (υ_e_ = 269–350 mol m^–3^). An expected decrease in the cross-link density was observed when long and flexible Pri was incorporated in the CAN networks along with Jeff (υ_e_ = 238–156 mol m^–3^). The CANs solely composed of the long and flexible Pri displayed the lowest cross-link density (υ_e_ = 123–206 mol m^–3^), and for IS-TREN, no rubbery plateau was obtained, since a continuous decrease of *E*′ revealed that this CAN is in the terminal zone, where the elastic dominance is no longer present, but a viscous one prevails (Figure S48). It was also observed that when IS-TREN samples were withdrawn after DMA analysis, a significant number of blisters were present in the sample, revealing some sort of thermal degradation that ultimately will make the stress relaxation behavior at high temperatures impossible to study. In addition, less toxic and safer alternatives to TREN, such as Jeff, have been reported.^45^ Therefore, we decided not to further evaluate CANs with TREN. In order to highlight the contribution of rigidity of the IH core of the prepared CANs, we prepared an analogous linear AAc using 1,4-butanediol, thereby keeping the same distance between reactive points as that for the IH-based CANs. The monomer 1,4-BD-AAc (Figures S15, S16, and S30) and the subsequent CAN BD-Pri_1_-Jeff_2_ (Figures S44) were successfully prepared in the same fashion as IH-AAc. Unsurprisingly, BD-Pri_1_-Jeff_2_ exhibited a *T*_g_ of 7 °C (Figure S49), a value significantly lower compared to the homologous IH-Pri_1_-Jeff_2_, proving the effect of the IH core.

The dynamic character of the synthesized CANs was evaluated by stress relaxation experiments. A constant strain of 2% was applied, and the relaxation modulus was recorded as a function of time in a temperature range from 80 to 200 °C (Figures 3 and S50–S61). The relaxation behavior of the prepared CANs showed dependence on the amines employed and to some extent on the calculated cross-link density, where the relaxation times for IH-Pri (104–216 s at 140 °C) were the fastest. The presence of nonbulky substituents in the alfa position for CANs with Pri seems to facilitate the dynamicity of the whole network. When Pri was partially replaced by Jeff, i.e., IH-Pri_1_-Jeff_2_, the observed relaxation times (180–647 s at 140 °C) increased due to the incorporation of Jeff, which portrays a lower nucleophilicity compared to Pri due to the bulkier methyl groups located in the alfa position.^45^ Finally, the IH-Jeff-based CANs exhibited the longest relaxation times (777–932 s at 140 °C), which is in agreement and correlates with the highest cross-link density (υ_e_) (269–350 mol m^–3^). The higher the cross-link density, the longer time needed to dissipate the stress.^46^ The use of less nucleophilic amines still render a quantitative conversion of the acetoacetate groups, as confirmed by FTIR analysis, matching literature examples, where Jeff was employed along with some more nucleophilic amines such as TREN.^30,45^ In conjunction with the cross-linking density, another determining variable for the stress relaxation times is *T*_g_.^47^ The lower the *T*_g_ of the CANs, the faster the CANs were able to relax. This can also be related to the chain mobility of the CANs prepared, as the Pri-based CANs had a higher chain mobility compared to the Jeff-based CANs, as observed vide infra related to the mechanical properties of the IH-based CANs. As the networks relaxed following a Maxwell model, it was possible to adjust to the Arrhenius law and ultimately calculate the activation energy (*E*_a_). The obtained *E*_a_ varied from 44.1 to 70.6 kJ mol^–1^ (see Supporting Information for calculation details), values that are in good agreement with other vinylogous urethane-based CANs previously reported.^48,49^ In addition to the *T*_g_ typically observed in amorphous polymers like thermosets, CANs also portray a secondary thermal transition known as the topology freezing transition temperature (*T*_v_). This transition indicates the temperature at which the bond exchange takes place within the network, and above this temperature, the material behaves more like a viscoelastic liquid, and therefore, the material can be reprocessed like a thermoplastic.^50–52^ In all the examples, the calculated values (see Supporting Information for calculation details) displayed a William–Landel–Ferry behavior in which *T*_v_ < *T*_g_.^12^

**Figure 3:**
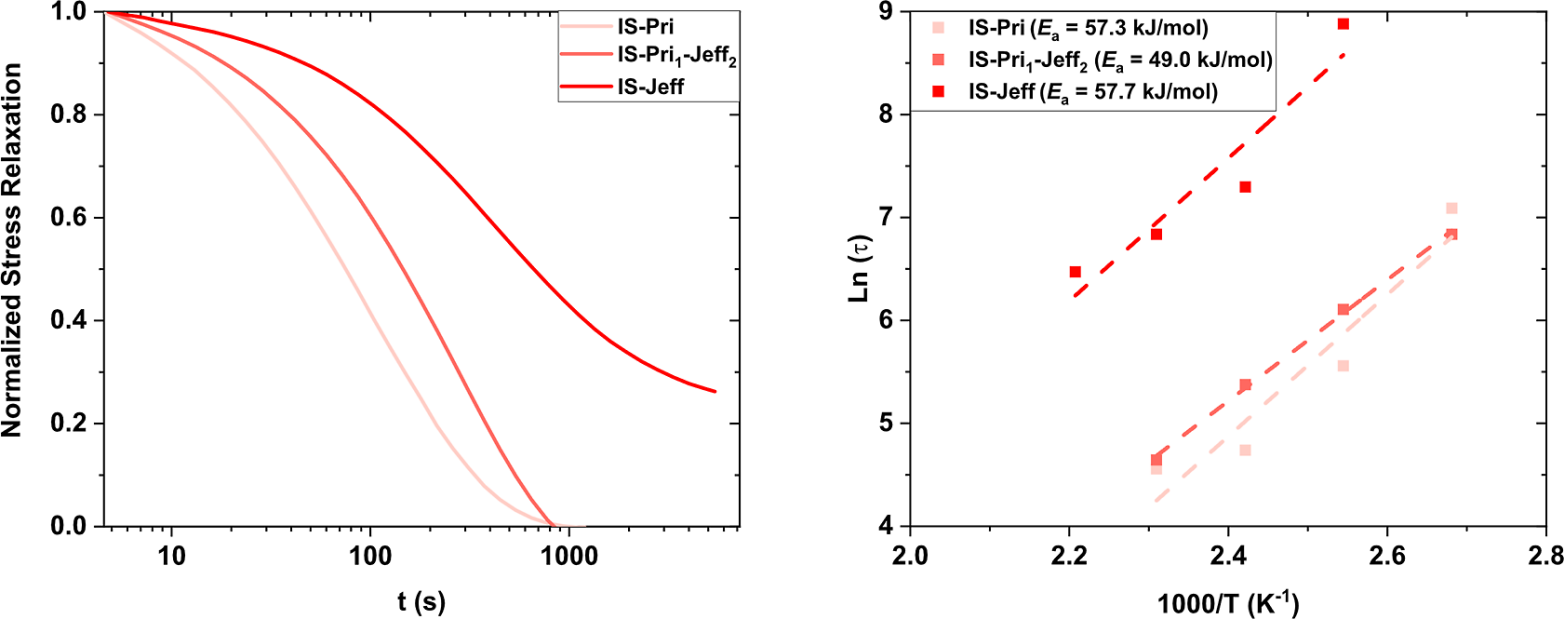
Stress relaxation curves of IS-based CANs at 140 °C (left) and the Arrhenius plot obtained from the relaxation times τ* used to calculate the *E*_a_ of IS-based CANs (right).

One of the main virtues is their reprocessability and subsequent recyclability as a consequence of the rapid dynamic rearrangements taking place within the network, which are oddities in classical thermosets. However, the reversible chemical reactions taking place within the network comes at the expenses of permanent deformation at higher temperatures than *T*_g_, and it is commonly known as creep. Therefore, creep resistance experiments were performed in order to assess the tendency of this permanent deformation typically observed in CANs under mechanical stress at temperatures higher than *T*_g_.^53–55^ Creep resistance experiments were performed for IS-Pri_1_-Jeff_2_ in a temperature range of 60–120 °C applying a constant stress for 20 min, followed by stress release and monitorization of the deformation of the CAN (Figure 4). As expected, at higher temperatures, the creep resistance was lower as a consequence of the faster reaction exchange kinetics occurring in the CANs. IS-Pri_1_-Jeff_2_ showed irreversible deformation regardless of the temperature, and after the stress was released and an elastic recovery of the deformation was observed, further elongation of the material was observed, especially when the material was tested at 120 °C. This behavior can be attributed to the very rapid exchange reactions taking place in this example.

**Figure 4:**
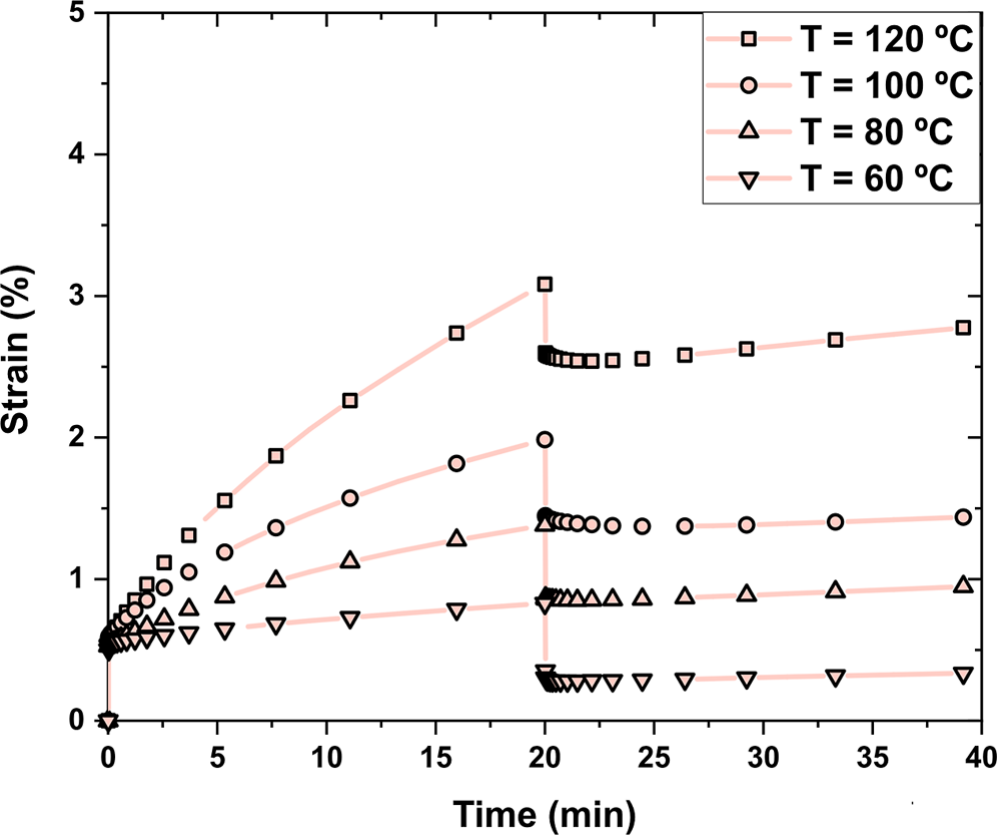
Creep experiments of IS-Pri_1_-Jeff_2_ with an applied stress of 0.01 MPa for 20 min.

The CANs mechanical properties depend on both the IH stereochemistry and the amine structure, where the amine structure had a larger influence on the mechanical properties (Figures 5 and 6). A quick and straightforward molecular mechanics (MM2) calculation revealed that the disposition of the hydroxyl groups in every IH core has an effect on where the acetoacetate functional groups are located and also on the distance between the cross-linking points within the network (Figure S62).^56^ We previously demonstrated the influence of the stereochemistry of the IH core on the thermal properties, and now we extended this to the mechanical properties. Thus, after stress–strain experiments, it was observed that the obtained values for BD-Pri_1_-Jeff_2_ (Figure S63 and Table S2, entry 19) were outnumbered even by the most elastic, fully Pri-based CAN IS-Pri. The CANs prepared ranged from soft and ductile materials when Pri was solely used as a cross-linking agent with low Young’s modulus (*E*), low strength at break (σ_b_), and high elongation at break (ε_b_) (Table S2, entries 1, 5, 6, and 7) to brittle and rigid CANs when short and rigid Jeff was used as a cross-linking agent. The Jeff-containing materials exhibited a remarkable *E* and a high σ_b_, yet at the same time, ε_b_ was very low as a consequence of the high cross-linking density (Table S2, entries 15–18). In between these examples, CANs bearing ratios of both Pri and Jeff, i.e., IH-Pri_1_-Jeff_2_, brought both toughness and elasticity (Table S2, entries 8, 12, 13, and 14). The IH stereochemistry also influenced the mechanical properties of the soft and ductile Pri-based CANs, where the distance between cross-links is the largest. When this distance is short, chain mobility is restricted by the cross-links and the influence of stereochemistry should be the smallest or nonexisting, while when the distance is larger, chain mobility is a distinct possibility and therefore stereochemical effects are possible (Table S2, entries 1, 5, 6, and 7). The stereoregular (*endo-endo* and *exo-exo*), i.e., IM-Pri and II-Pri, CANs had higher σ_b_ compared with the nonstereoregular (*endo-exo*) counterpart IS-Pri, having the lowest σ_b_ and the highest ε_b_. A loss of stereoregularity thereby leads to a decrease in strength, similarly to what was previously observed in other IH-based thermoplastic systems, where chain mobility is not restricted by cross-linking points.^57^ II-Pri was less elastic compared to IM-Pri, while the σ_b_ was unaffected (Table S2, entries 5 and 6). This indicates that the *exo-exo* conformation of IM-Pri renders a more elastomeric material in contrast with the *endo-endo* conformation present in II-Pri that displays more plastic behavior. In addition, an example using equimolar amounts of opposite stereoisomers IM_0.5_-II_0.5_-Pri was prepared (Table S2, entry 7). To our delight, this CAN showed the highest σ_b_ and an ε_b_ between those observed for CANs composed of one IH stereoisomer. Hence, despite the cross-linked nature of the CANs, there is a slight steric effect within the network influencing the mechanical properties if the distance between cross-links is large enough. A similar trend and behavior were observed for the IH-Pri_1_-Jeff_2_ CANs (Table S2, entries 8, 12, 13, and 14). For these CANs, an increase in *E* and σ_b_ was observed as a consequence of the incorporation of a shorter and more rigid amine like Jeff in detriment of Pri, which render materials with an overall higher *E* and σ_b_ while at the same time the ε_b_ approximately remained with the same range of values, resulting in a significant increase in the toughness. The IM-containing CAN (IM-Pri_1_-Jeff_2_) showed the most elastomeric behavior of the four examples tested, with the lowest *E* and σ_b_ values and the highest ε_b_ values (Table S2, entry 12). On the contrary, II-Pri_1_-Jeff_2_ had a higher *E*, slightly higher σ_b_, and a noticeably reduced ε_b_ (Table S2, entry 13). Likewise, IM_0.5_-II_0.5_-Pri_1_-Jeff_2_ exhibited a steric effect since an increase of σ_b_ and a subsequent ε_b_ between both stereoisomers on their own was obtained (Table S2, entry 14). On the contrary, for the fully Jeff-based CANs (Table S2, entries 15–18), no significant differences were observed based on the stereoisomer utilized. This observation can be attributed to the high cross-linking density, making any stereoisomer effect limited. When the network is densely cross-linked, any sort of secondary interactions via hydrogen bonding or steric effects derived from the nature of the IH used are insignificant and thereby do not affect the mechanical properties because of the short distance between cross-links.

**Figure 5:**
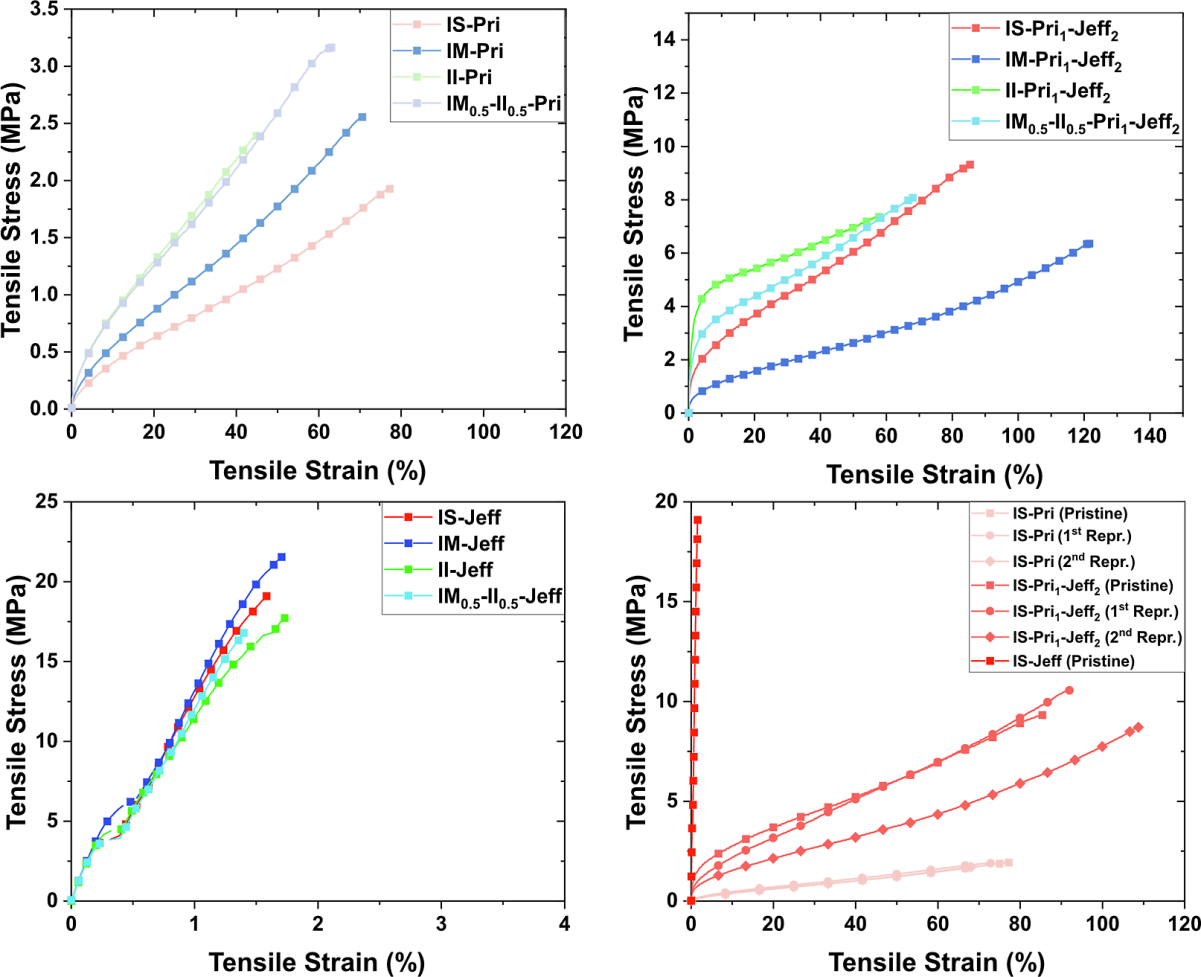
Stress–strain curves for CANs IH-Pri (top left), stress–strain curves for CANs IH-Pri_1_-Jeff_2_ (top right), stress–strain curves for CANs IH-Jeff (bottom left), and overlap stress–strain curves for CANs IS-Pri_*x*_-Jeff_*y*_ with the 1st, 2nd, and 3rd reprocessed curves of IS-Pri_1_ and IS-Pri_1_-Jeff_2_ (bottom right).

**Figure 6:**
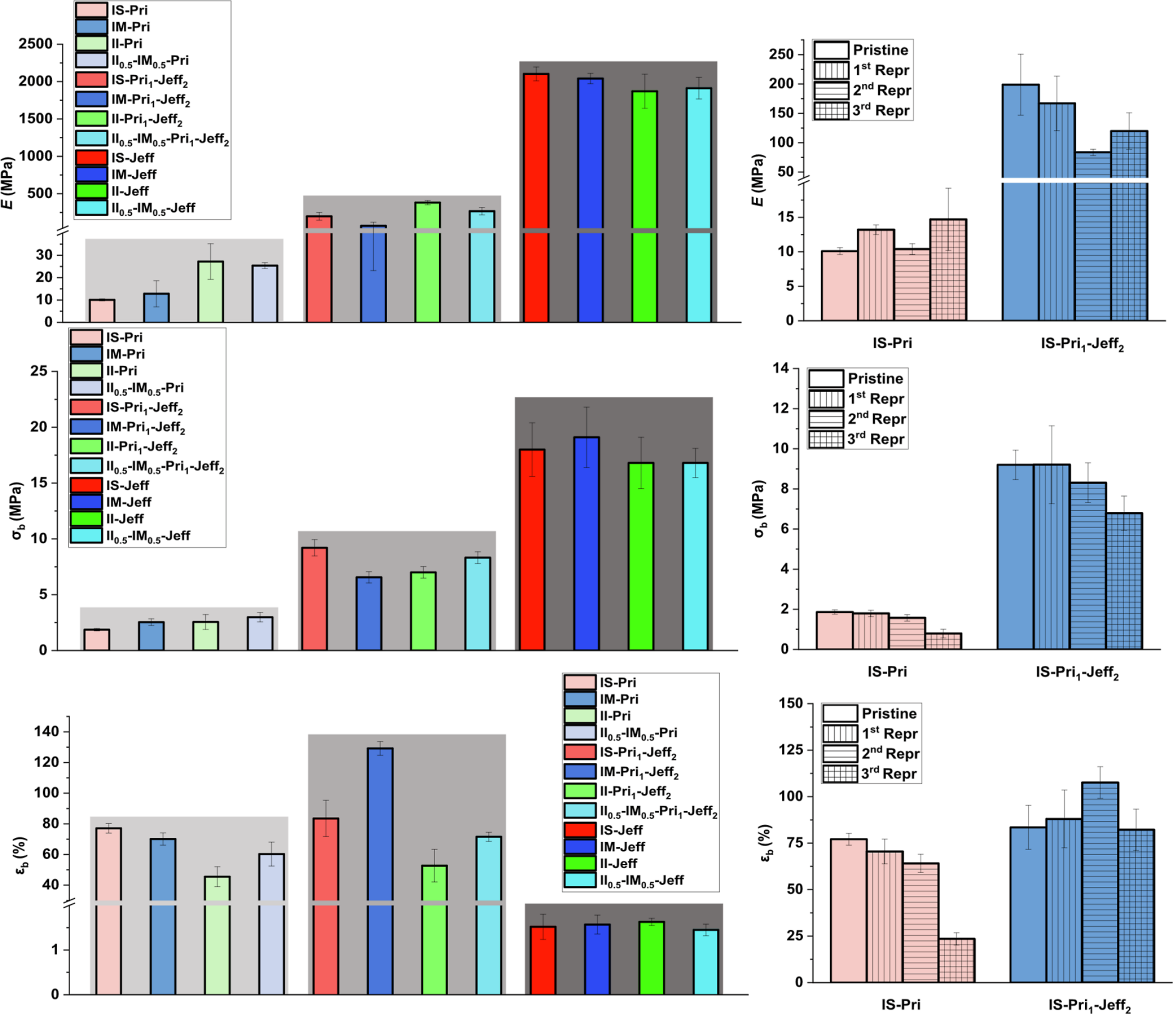
Young’s modulus (*E*) of IH-based vinylogous urethane CANs (top left), *E* of the pristine and the first, second, and third reprocessing of CANs IS-Pri and IS-Pri_1_-Jeff_2_ (top right), tensile strength at break (σ_b_) of IH-based vinylogous urethane CANs (middle left), σ_b_ of the pristine and the first, second, and third reprocessing of CANs IS-Pri and IS-Pri_1_-Jeff_2_ (middle right), elongation at break (ε_b_) of IH-based vinylogous urethane CANs (bottom left), and ε_b_ of the pristine and the first, second, and third reprocessing of CANs IS-Pri and IS-Pri_1_-Jeff_2_ (bottom right).

A characteristic feature of CANs is their reprocessability as a consequence of the reversible chemical reactions taking place within the network upon a trigger. In this study, we proved that the CANs prepared were reprocessable. IS-Pri_1_-Jeff_2_ retained its mechanical properties after three reprocessing cycles (Figures 5 and 6 and Table S2, entries 8–11), while IS-Pri retained its properties for the first and second reprocessing cycles, whereas after the third reprocessing, a large decay of the mechanical performance was observed (Figures 5 and 6 and Table S2, entries 1–4). This behavior can be attributed to a possible oxidation of the amines in excess during hot press reprocessing, which ultimately hampers the reversible reactions taking place in the network that allow the reprocessing of the materials. Furthermore, we tested the self-healing and the reshaping properties of IS-Pri_1_-Jeff_2_, properties complementary to the ability of being reprocessable. The CAN displayed both malleability and self-healing behavior (Figure 7). Two small rectangular pieces were cut and self-healed after they were placed in an oven at 140 °C for 1 h. Reshaping after heating was also demonstrated. A rectangular specimen was bent in three different points at room temperature, and after being placed in an oven for 1 min, it quickly recovered its original shape. This observed behavior is typical from CANs due to the thermally triggered reversible mechanism present within the network.^45,58^

**Figure 7:**
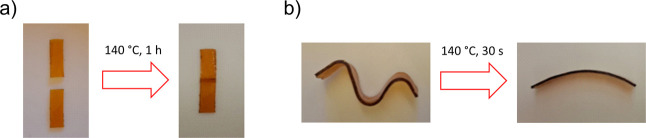
Self-healing experiment of IS-Pri_1_-Jeff_2_ (a) and the shape memory experiment of IS-Pri_1_-Jeff_2_ (b).

Vinylogous urethane moieties are well-known to undergo hydrolysis under acidic conditions following a similar mechanism to those observed for imines.^59^ The amine becomes protonated in the presence of an acid rendering an iminium functionality, making it a good leaving group. Subsequently a molecule of water attacks the electrophilic carbon, kicking out the iminium group, and the primordial acetoacetate is obtained. As expected, when the CANs were immersed in an aqueous solution of HCl (1 M) at room temperature, the CANs degraded to their monomeric counterparts and were solubilized in the aqueous media. An interesting observation to note is that for the IH-Pri CANs, no degradation was observed and the CAN structure prevailed, most likely due to the high hydrophobicity of the long and flexible Pri that hindered the hydrolysis process. This behavior simultaneously broadens the application scope for these CANs while hindering the degradability and possible chemical recycling.^60^

A second approach to achieve degradation was performed via amine exchange within the network when treated with a monoamine.^38^ IS-Pri_*x*_-Jeff_*y*_ (100 mg) was suspended in EtOH (10 mL) with an excess of benzyl amine (1 mL) and stirred for 24 h at room temperature (Figure S54). After a few hours, the CANs swelled, and IS-Pri_1_-Jeff_2_ and IS-Jeff were dissolved, highlighting the dynamic process taking place within the network (Scheme S6). The reaction taking place is the benzyl amine reacting with the Pri- and Jeff-based vinylogous urethane moieties displacing the amines used as cross-linkers, i.e., Pri and Jeff, respectively, and a bis-benzylamine vinylogous urethane is obtained. These molecules are soluble in EtOH, and therefore, the network is degraded, forming the original amines and bis-benzylamine vinylogous urethane. On the contrary, for IS-Pri, the CAN swelled, but no degradation of the network was observed. This can be attributed to the presence of the hydrophobic cross-linker Pri, and it is possible that given time or an increased temperature chemical degradation could also be achieved.

## Conclusions

In this work, a series of biobased vinylogous urethane CANs were prepared by reacting IH-AAc monomers with a long flexible fatty acid-derived amine and a short and rigid triamine. The physical properties of CANs could be tailored depending on the IH stereoisomer and the ratios and nature of the amines employed. A wide range of *T*_g_ from 20 to 114 °C was achieved, tailorable by the amine structure and independent of the stereoisomer used. The mechanical properties displayed were strongly influenced not only by the amine employed, where long flexible fatty acid-derived Priamine 1071 led to more elastomeric materials and short and rigid Jeffamine T-403 led to more plastic materials, but also by the stereoisomer utilized when the distance between cross-links was long, which was the case for the IH-Pri and IH-Pri_1_-Jeff_2_ CANs. For the most elastomeric CANs, IH-Pri, stereoregular II-Pri, and IM-Pri exhibited both increased Young’s modulus and strength at break values compared to the nonstereoregular IS-Pri CAN. Steric effects were observed for the CAN IM_0.5_-II_0.5_-Pri with a slight increase in strength at break (from 2.54 ± 0.30 and 2.55 ± 0.67 to 2.98 ± 0.43 MPa) and an elongation at break value which was in between the ones observed for their constituents individually (from 70.1 ± 4.0 and 45.5 ± 6.5 to 60.3 ± 7.8%), i.e., IM-Pri and II-Pri, respectively. The same effect was also observed for the mixed CANs based on two different amines (IH-Pri_1_-Jeff_2_). IH-Jeff CANs had the shortest distance between cross-links, and in this scenario, no effect on the properties based on the stereoisomer employed was seen. Examples of the designed CANs were reprocessed twice with retained mechanical performance as a consequence of the dynamic bonds present within the network. The reversibility of the vinylogous urethane moieties present in the CANs was also proven via stress relaxation experiments, and chemical degradation was proven by treating the prepared CANs with an excess of benzylamine, opening up for future chemical recycling which enables the development of sustainable closed-loop thermosets.

## Supplementary Material



## Notes

NMR characterization of monomers, FTIR analysis, TGA curves, stress relaxation experiments, stress–strain curves, and calculations for *E*_a_ and *T*_v_ (PDF)
