# Supplementary material for: Effects of Isohexide Stereochemistry on Vinylogous Urethane Covalent Adaptable Networks
Source: Macromolecules. 2025 Sep 29;58(19):10714–24. doi: 10.1021/acs.macromol.5c00270 (PMC12530046; doi:10.1021/acs.macromol.5c00270)
Supplement: Supplementary file 1 [file ma5c00270_si_001.pdf]

Effects of Isohexides Stereochemistry on Vinylogous Urethane Covalent Adaptable Networks

Noé Fanjul-Mosteirín,[1] and Karin Odelius[1]*

[1]Department of Fibre and Polymer Technology, KTH Royal Institute of Technology, SE-100 44, Stockholm, Sweden

*Corresponding author: Karin Odelius, hoem@kth.se

Table of Contents

Synthesis of isohexide acetoacetate (IH-AAc) ... 6

Scheme S1. Synthesis of IS-AAc. ... 6

Scheme S2. Synthesis of IM-AAc. ... 6

Scheme S3. Synthesis of II-AAc. ... 6

Synthesis of isomannide tosylate (IM-Ts) ... 6

Synthesis of isomannide acetate (IM-Ac) ... 7

Synthesis of isoidide (II) ... 7

Synthesis of isohexides hexyl-based vinylogous urethane (IS-Hx-VU and IM-Hx-VU) ... 7

Scheme S4. Synthesis of isosorbide and isomannide hexyl vinylogous urethane (IS-Hx-VU and IM-Hx-VU) ... 8

Model reactions for the VU exchange ... 8

Scheme S5. Isosorbide and isomannide vinylogous urethane reaction exchange with benzyl amine. ... 9

^{1}H and ^{13}C NMR spectra ... 10

Figure S1. ^{1}H NMR spectrum of IS-hexylamine-based vinylogous urethane (400 MHz, 298 K, $CDCl_3$). Unassigned peaks correspond to excess of hexylamine employed. ... 10

Figure S2. ^{1}H NMR spectrum of IM-hexylamine-based vinylogous urethane (400 MHz, 298 K, $CDCl_3$). Unassigned peaks correspond to excess of hexylamine employed. ... 10

Figure S3. ^{1}H NMR spectrum of IS-AAc (400 MHz, 298K, $CDCl_3$). Unassigned peaks correspond to enol tautomer. ... 11

Figure S4. ^{13}C APT NMR spectrum of IS-AAc (100 MHz, 298 K, $CDCl_3$). ... 11

Figure S6. ^{13}C APT NMR spectrum of IM-AAc (100 MHz, 298 K, $CDCl_3$). 12
Figure S8. ^{13}C APT NMR spectrum of Di-tosyl isomannide (100 MHz, 298 K, $CDCl_3$). .. 13
Figure S9. ^{1}H NMR spectrum of Di-acetyl isoidide (400 MHz, 298 K, $CDCl_3$). 14
Figure S10. ^{13}C APT NMR spectrum of Di-acetyl isoidide (100 MHz, 298 K, $CDCl_3$). 14
Figure S11. ^{1}H NMR spectrum of Isoidide (400 MHz, 298 K, D_2O). 15
Figure S12. ^{13}C APT NMR spectrum of Isoidide (100 MHz, 298 K, D_2O). 15
Figure S13. ^{1}H NMR spectrum of II-AAc (400 MHz, 298K, $CDCl_3$). Unassigned peaks correspond to enol tautomer. 16
Figure S14. ^{13}C APT NMR spectrum of IM-AAc (100 MHz, 298 K, $CDCl_3$). 16
Figure S15. ^{1}H NMR spectrum of 1,4-BD-AAc (400 MHz, 298K, $CDCl_3$). 17
Figure S16. ^{13}C APT NMR spectrum of 1,4-BD-AAc (100 MHz, 298 K, $CDCl_3$). 17
Figure S17. ^{1}H NMR spectra of IS-Hx-VU exchange with Bn-NH_2 at T = 80 °C (400 MHz, 298 K, d_6-DMSO). 18
Figure S18. ^{1}H NMR spectra of IM-Hx-VU exchange with Bn-NH_2 at T = 80 °C (400 MHz, 298 K, d_6-DMSO). 18
Figure S19. ^{1}H NMR spectra of IS-Hx-VU exchange with Bn-NH_2 at T = 100 °C (400 MHz, 298 K, d_6-DMSO). 19
Figure S21. ^{1}H NMR spectra of IS-Hx-VU exchange with Bn-NH_2 at T = 120 °C (400 MHz, 298 K, d_6-DMSO). 20
Figure S22. ^{1}H NMR spectra of IM-Hx-VU exchange with Bn-NH_2 at T = 120 °C (400 MHz, 298 K, d_6-DMSO). 20
Kinetic plots for the model reaction vinylogous urethane exchanges 21
Figure S23. Decrease of IS-Hx-VU and IM-Hx-VU as a function of time at T = 80 °C when treated with an excess of Bn-NH_2. 21
Figure S24. Decrease of IS-Hx-VU and IM-Hx-VU as a function of time at T = 100 °C when treated with an excess of Bn-NH_2. 21
Figure S25. Decrease of IS-Hx-VU and IM-Hx-VU as a function of time at T = 120 °C when treated with an excess of Bn-NH_2. 22
Arrhenius plots for the model reaction vinylogous urethane exchanges 22

Figure S26. Arrhenius plot on the exchange reaction of IS-Hx-VU with Bn-NH_2 (*left*) and Arrhenius plot on the exchange reaction of IM-Hx-VU with Bn-NH_2 (*right*)....................22

FTIR 23

Figure S27. FTIR spectrum of monomer IS-AAc. 23

Figure S28. FTIR spectrum of monomer IM-AAc. 23

Figure S29. FTIR spectrum of monomer II-AAc. 24

Figure S30. FTIR spectrum of monomer 1,4-BD-AAc. 24

Figure S31. FTIR spectrum of network IS-Pri after curing process 140 °C during 8 h.......25

Figure S32. FTIR spectrum of network IM-Pri after curing process 140 °C during 8 h. 25

Figure S33. FTIR spectrum of network II-Pri after curing process 140 °C during 8 h. 26

Figure S34. FTIR spectrum of network $IM_{0.5}$-$II_{0.5}$-Pri after curing process 140 °C during 8 h. 26

Figure S35. FTIR spectrum of network IS-Pri_1-$Jeff_2$ after curing process 140 °C during 8 h. 27

Figure S36. FTIR spectrum of network IM-Pri_1-$Jeff_2$ after curing process 140 °C during 8 h. 27

Figure S37. FTIR spectrum of network II-Pri_1-$Jeff_2$ after curing process 140 °C during 8 h. 28

Figure S38. FTIR spectrum of network $IM_{0.5}$-$II_{0.5}$-Pri_1-$Jeff_2$ after curing process 140 °C during 8 h. 28

Figure S39. FTIR spectrum of network IS-Jeff after curing process 140 °C during 8 h. 29

Figure S40. FTIR spectrum of network IM-Jeff after curing process 140 °C during 8 h....29

Figure S41. FTIR spectrum of network II-Jeff after curing process 140 °C during 8 h. 30

Figure S42. FTIR spectrum of network $IM_{0.5}$-$II_{0.5}$-Jeff after curing process 140 °C during 8 h. 30

Figure S43. FTIR spectrum of network IS-TAEA after curing process 140 °C during 8 h.31

Figure S44. FTIR spectrum of network BD-Pri_1-$Jeff_2$ after curing process 140 °C during 8 h. 31

Gel content experiments 32

Figure S45. Gel content experiments for IS-Pri (*left*) and IS-Pri$_1$-Jeff$_2$ (*right*). Pictures on the top correspond to t = 0 and pictures at the bottom correspond to t = 24 h. 32

Table S1. Gel content after immersion at r.t in different solvents of IH-based vinylogous CANs. 32

TGA results 33

Figure S46. TGA curves of monomers IS-AAc, IM-AAc, II-AAc, IS-TAEA (*top*) and CANs IH-Pri$_x$-Jeff$_y$ (*bottom*). 33

DSC analysis 34

Figure S47. DSC curves (2nd heating scan) of II-Pri, II-Pri$_1$-Jeff$_2$, II-Jeff. 34

DMA analysis 35

Figure S48. DMA of CAN IS-TAEA 35

Figure S49. DMA of CAN BD-Pri$_1$-Jeff$_2$ 35

Stress relaxation experiments and Arrhenius obtained plots 36

Figure S50. Stress relaxation curve of IS-Pri (*left*) and Arrhenius plot obtained from the relaxation times τ^* used to calculate the E_a of IS-Pri (*right*). 36

Figure S51. Stress relaxation curve of IM-Pri (*left*) and Arrhenius plot obtained from the relaxation times τ^* used to calculate the E_a of IM-Pri (*right*). 36

Figure S52. Stress relaxation curve of II-Pri (*left*) and Arrhenius plot obtained from the relaxation times τ^* used to calculate the E_a of II-Pri (*right*). 37

Figure S53. Stress relaxation curve of IM$_{0.5}$-II$_{0.5}$-Pri (*left*) and Arrhenius plot obtained from the relaxation times τ^* used to calculate the E_a of IM$_{0.5}$-II$_{0.5}$-Pri (*right*). 37

Figure S54. Stress relaxation curve of IS-Pri$_1$-Jeff$_2$ (*left*) and Arrhenius plot obtained from the relaxation times τ^* used to calculate the E_a of IS-Pri$_1$-Jeff$_2$ (*right*). 38

Figure S55. Stress relaxation curve of IM-Pri$_1$-Jeff$_2$ (*left*) and Arrhenius plot obtained from the relaxation times τ^* used to calculate the E_a of IM-Pri$_1$-Jeff$_2$ (*right*). 38

Figure S56. Stress relaxation curve of II-Pri$_1$-Jeff$_2$ (*left*) and Arrhenius plot obtained from the relaxation times τ^* used to calculate the E_a of II-Pri$_1$-Jeff$_2$ (*right*). 39

Figure S57. Stress relaxation curve of IM$_{0.5}$-II$_{0.5}$-Pri$_1$-Jeff$_2$ (*left*) and Arrhenius plot obtained from the relaxation times τ^* used to calculate the E_a of IM$_{0.5}$-II$_{0.5}$-Pri$_1$-Jeff$_2$ (*right*). 39

Figure S58. Stress relaxation curve of IS-Jeff (*left*) and Arrhenius plot obtained from the relaxation times τ^* used to calculate the E_a of IS-Jeff (*right*). 40

Figure S59. Stress relaxation curve of IM-Jeff (*left*) and Arrhenius plot obtained from the relaxation times τ^* used to calculate the E_a of IM-Jeff (*right*). 40

Figure S60. Stress relaxation curve of II-Jeff (*left*) and Arrhenius plot obtained from the relaxation times τ^* used to calculate the E_a of II-Jeff (*right*). 41

Figure S61. Stress relaxation curve of $IM_{0.5}$-$II_{0.5}$-Jeff (*left*) and Arrhenius plot obtained from the relaxation times τ^* used to calculate the E_a of $IM_{0.5}$-$II_{0.5}$-Jeff (*right*). 41

Molecular mechanics (MM2) calculation 42

Figure S62. Ball and stick model for IS-AAc, IM-AAc and II-AAc using MM2 energy-minimized method. In grey and red carbon and oxygen atoms respectively. Hydrogen atoms were removed for simplicity. 42

Stress-strain curves 43

Figure S63. Stress-strain curve of BD-Pri_1-$Jeff_2$, numbers correspond to each sample run. 43

Calculations for stress relaxation derived from activation energy 44

Calculations for vitrimer temperature (T_v) using Arrhenius equation from stress relaxation 45

Mechanical properties of vinylogous urethane CANs 48

Table S2. Mechanical properties of vinylogous urethane CANs. 48

Chemical degradation 49

Figure S64. IS-Pri_x-$Jeff_y$ CANs in EtOH (*left*) and degraded IS-Pri_x-$Jeff_y$ after addition of benzyl amine and orbital stirring at room temperature for 24 h (*right*). 49

Scheme S6. Mechanism for the chemical degradation of CANs. 49

References 49

Synthesis of isohexide acetoacetate (IH-AAc)

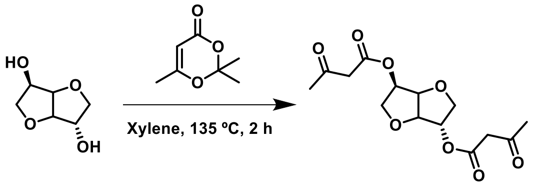

Scheme S1. Synthesis of **IS-AAc.**

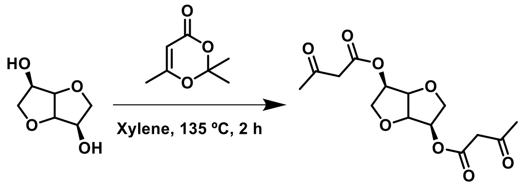

Scheme S2. Synthesis of **IM-AAc.**

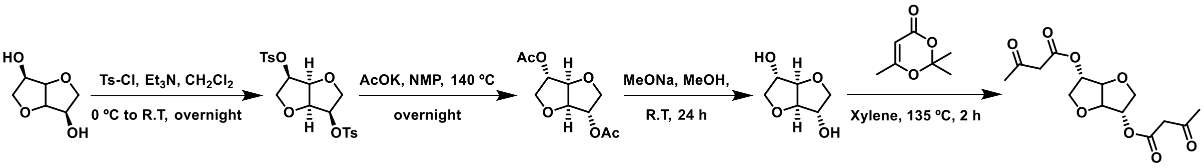

Scheme S3. Synthesis of **II-AAc.**

Synthesis of isomannide tosylate (IM-Ts)

A 250 mL round bottom flask was charged with isomannide (20.0 g, 136.86 mmol, 1 equiv.), triethylamine (47.7 mL, 342.14 mmol, 2.5 equiv.), *N,N*-4-dimethylaminopyridine (DMAP) (1.67 g, 13.69 mmol, 0.1 equiv.) and CH_2Cl_2 (200 mL). The reaction mixture was cooled down to 0 °C and then, tosyl chloride (58,4 g, 287.4 mmol, 2.1 equiv.) was added portion wise over several minutes. After the addition was complete, the reaction was stirred at room temperature overnight. The mixture was transferred into a 500 mL separatory funnel and washed with 1M HCl (1 x 100 mL), water (2 x 100 mL), $NaHCO_3$ (1 x 100 mL) and brine (1 x 100 mL). The remaining organic phase was dried over Na_2SO_4 and concentrated to afford a brownish solid which was further purified by recrystallization in EtOH (aprox. 200 mL) (55.92 g obtained, 123.17 mmol, 90% yield). Characterization data was in agreement with those reported in the literature.[1] **^{1}H NMR** (400 MHz, $CDCl_3$) δ 7.80 (d, 4H, $^3J_{H\text{-}H}$ = 8.4 Hz, CH-C-SO_3), 7.34 (d, 4H, $^3J_{H\text{-}H}$ = 8.1 Hz, C*H*-C-CH_3), 4.88-4.78 (m, 2H, CH-OSO_2), 4.48-4.45 (m, 2H, C*H*-O-CH_2), 3.91 (dd, 2H, $^3J_{H\text{-}H}$ = 9.5 and 6.7 Hz, CH_{anti}-CH-OSO_2), 3.72 (dd, 2H, $^3J_{H\text{-}H}$ = 9.6 and 7.7 Hz, CH_{syn}-CH-OSO_2), 2.45 (s, 6H, CH_3). **^{13}C APT NMR** (100 MHz, $CDCl_3$) δ 145.4 (C-SO_3), 133.2 (*C*-

CH_3), 130.1 (*C*H-C-CH_3), 128.1 (*C*H-C-SO_3), 80.1 (C-OSO_2), 78.0 (*C*H-CH-OSO_2), 70.2 (*C*H_2-CH-OSO_2), 21.8 (CH_3).

Synthesis of isomannide acetate (IM-Ac)

A 500 mL round bottom flask was charged with isomannide tosylate (55.80 g, 122.77 mmol, 1.0 equiv), potassium acetate (72.29 g, 736.62 mmol, 6.0 equiv) and 186 mL NMP. A condenser was attached to the RBF and the reaction was heated to 130 °C and checked by TLC overnight (starting material finished). The reaction mixture was diluted in 100 mL of water and transferred to a separation funnel. The aqueous phase was extracted twice with EtOAc and the combined organic phases were washed 3 times with brine. The organic phase was dried over Na2SO4 filtered and concentrated under high vacuum. A pale brownish oil was obtained (26.52 g obtained, 115.40 mmol, 94% yield). Characterization data was in agreement with those reported in the literature.[1] **^{1}H NMR** (400 MHz, $CDCl_3$) δ 5.21-5.14 (m, 2H, CH-O-C=O), 4.62 (s, 2H, C*H*-O-CH_2), 3.97-3.88 (m, 4H, CH_2), 2.07 (s, 6H, CH_3). **^{13}C APT NMR** (100 MHz, $CDCl_3$) δ 170.1 (C=O), 86.4 (*C*H-O-C=O), 74.7 (*C*H-O-CH_2), 73.8 (CH_2), 21.0 (CH_3).

Synthesis of isoidide (II)

In a 2-necked 250 mL RBF, Isomannide acetate (20.75 g, 90.13 mmol, 1 equiv.) was dissolved in 74 mL of MeOH(dry). To this mixture, a solution of NaOH *i.e* MeONa (1.19 g, 29.74 mmol, 0.33 equiv.) in MeOH(dry) (16 mL) was added dropwise and the mixture was stirred overnight at room temperature (Checked starting material consumption by TLC). Dowex 50W X8 (4.45 g) was added and the mixture was stirred for 4 h at r.t. Reaction mixture was filtered over celite and the Dowex resin rinsed with MeOH (HPLC grade). The liquors were concentrated under high vacuum and a light yellowish solid was obtained. The crude was purified by flash column chromatography ($CHCl_3$/MeOH 9:1). (10.19 g obtained, 69.40 mmol, 77% yield). Characterization data was in agreement with those reported in the literature.[1] **^{1}H NMR** (400 MHz, D_2O) δ 4.64 (s, 2H, CH), 4.36 (d, 2H, $^3J_{H\text{-}H}$ = 3.2 Hz, C*H*-OH), 3.91-3.82 (m, 4H, CH_2). **^{13}C APT NMR** (100 MHz, D_2O) δ 86.4 (CH), 74.7 (CH-OH), 73.8 (CH_2).

Synthesis of isohexides hexyl-based vinylogous urethane (IS-Hx-VU and IM-Hx-VU)

In a 50 mL round bottomed flask, IH-AAc (1.51 g, 4.81 mmol, 1 equiv.) was dissolved in $CHCl_3$ (4.8 mL). Then, hexyl amine (1.40 mL, 10.57 mmol, 2.2 equiv.) was added and the mixture was stirred at room temperature for 4 h. The liquors were concentrated under high vacuum and the crude was purified by flash column chromatography (heptane/EtOAc 3:1). A

colorless oil was obtained (IS-Hx-VU 1.57 g obtained, 3.08 mmol, 64% yield) and IM-Hx-VU (1.66 g obtained, 3.32 mmol, 68% yield).

IS-Hx-VU **^{1}H NMR** (400 MHz, $CDCl_3$) 8.50 (bs, 2H, NH), 5.14-5.07 (m, 2H, $C_{(1)}$H-O-C=O and $C_{(5)}$H-O-C=O), 4.75 (t, 1H, $^3J_{H\text{-}H}$ = 4.7 Hz, C*H*-$C_{(5)}$H), 4.50-4.49 (m, 2H, C*H*-$C_{(1)}$H-O-C(=O)-C*H*=C), 4.39 (s, 1H, CH-$C_{(1)}$H-O-C(=O)-C*H*=C), 4.07-3.91 (m, 3H, CH_2-O-CH-$C_{(5)}$H-CH_{syn}), 3,72-3.66 (m, 1H, $C_{(5)}$H-CH_{anti}) 3.23-3.14 (m, 4H, NH-CH_2), 1.89 (s, 6H, C(=O)-CH_3), 1.54 (p, 4H, $^3J_{H\text{-}H}$ = 6.5 Hz, NH-CH_2-CH_2), 1.39-1.24 (m, 12H, NH-CH_2-CH_2-(CH_2)$_3$-CH_3), 0.87 (t, 6H, $^3J_{H\text{-}H}$ = 6.7 Hz, NH-CH_2-CH_2-(CH_2)$_3$-CH_3).

IM-Hx-VU **^{1}H NMR** (400 MHz, $CDCl_3$) δ 8.45 (bs, 2H, NH), 5.06-5.01 (m, 2H, CH-O-C=O), 4.64-4.62 (m, 2H, C*H*-CH-O-C=O), 4.47 (s, 2H, O-C(=O)-C*H*), 4.01 (dd, 2H, $^3J_{H\text{-}H}$ = 9.0 and 6.8 Hz, CH_{anti}-CH-O-C=O), 3.74 (dd, 2H, $^3J_{H\text{-}H}$ = 8.9 and 7.7 Hz, CH_{syn}-CH-O-C=O), 3.16-3.11 (m, 4H, NH-CH_2), 1.86 (s, 6H, C(=O)-CH_3), 1.54-1.47 (m, 4H, NH-CH_2-CH_2), 1.34-1.24 (m, 12H, NH-CH_2-CH_2-(CH_2)$_3$-CH_3), 0.84 (m, 6H, NH-CH_2-CH_2-(CH_2)$_3$-CH_3).

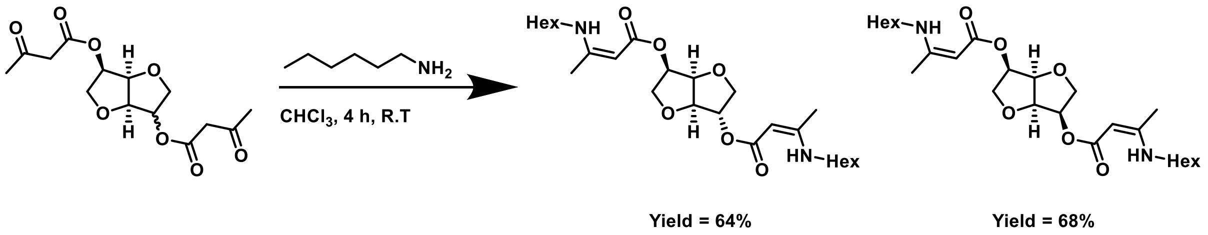

Scheme S4. Synthesis of isosorbide and isomannide hexyl vinylogous urethane (IS-Hx-VU and IM-Hx-VU).

Model reactions for the VU exchange

Benzylamine (55 µL, 0.50 mmol, 10 equiv.) was added to a solution of isohexide hexyl vinylogous urethane (IH-Hx-VU) (25 mg, 0.05 mmol, 1 equiv.) in d_6-DMSO (0.5 mL, [IH-Hx-VU] = 100 mM). Mixtures were kept at 80, 100 and 120 °C in a NMR tube in an oil bath and spectra were taken at predefined time intervals and the VU exchange was followed by integration of the NH-CH_2-Ph (4.22-4.20 ppm) *vs* NH-C$H_{2\ (cis)}$-CH_2-Bu (3.22-3.17 ppm) plus NH-C$H_{2\ (trans)}$-CH_2-Bu (2.94-2.88 ppm).

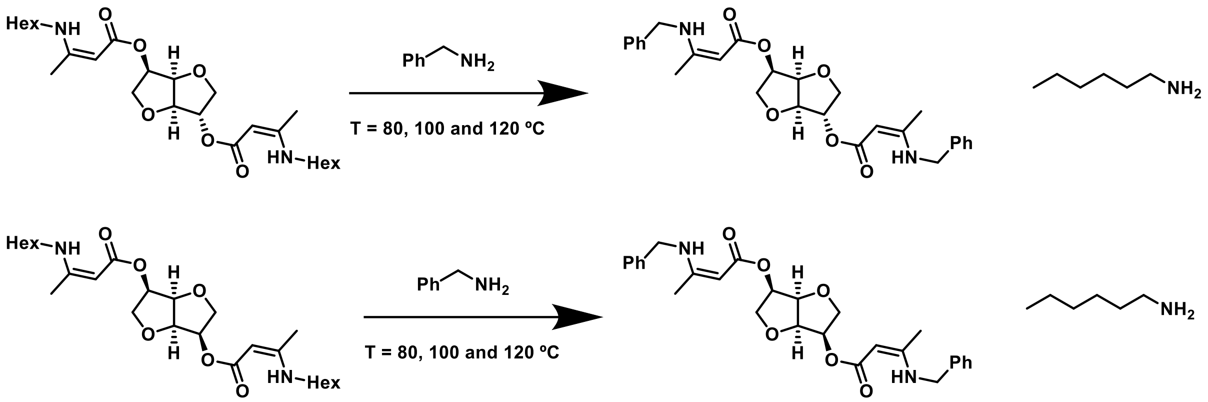

Scheme S5. Isosorbide and isomannide vinylogous urethane reaction exchange with benzyl amine.

^{1}H and ^{13}C NMR spectra

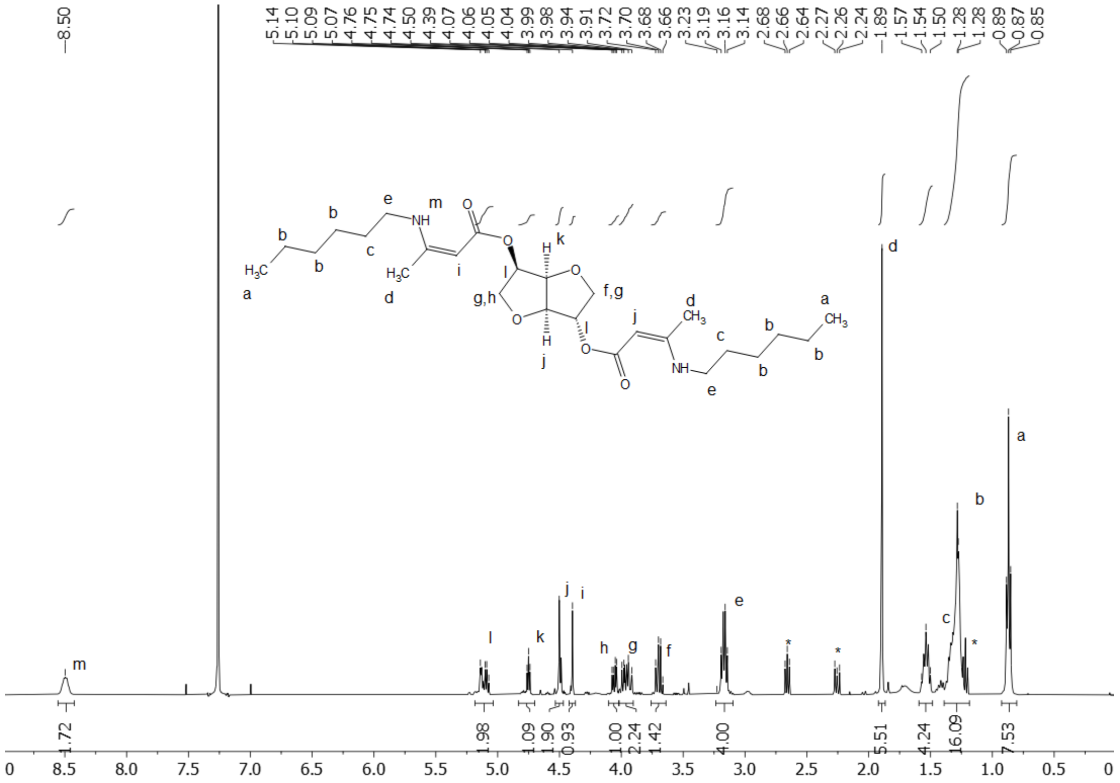

Figure S1. ^{1}H NMR spectrum of IS-hexylamine-based vinylogous urethane (400 MHz, 298 K, $CDCl_3$). Unassigned peaks correspond to excess of hexylamine employed.

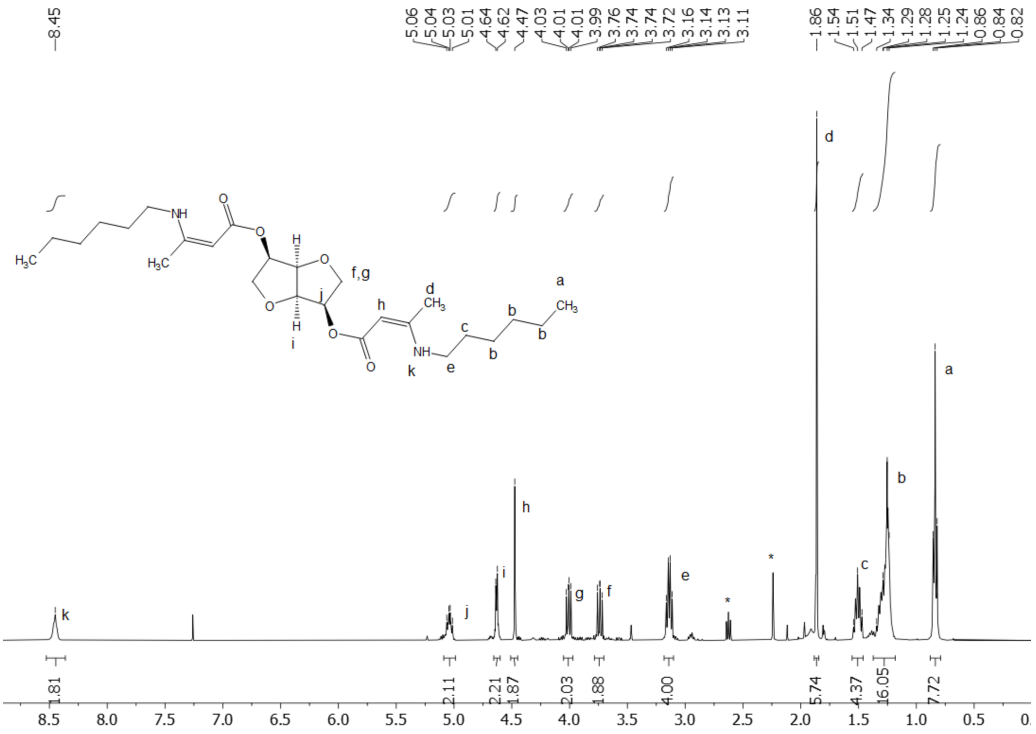

Figure S2. ^{1}H NMR spectrum of IM-hexylamine-based vinylogous urethane (400 MHz, 298 K, $CDCl_3$). Unassigned peaks correspond to excess of hexylamine employed.

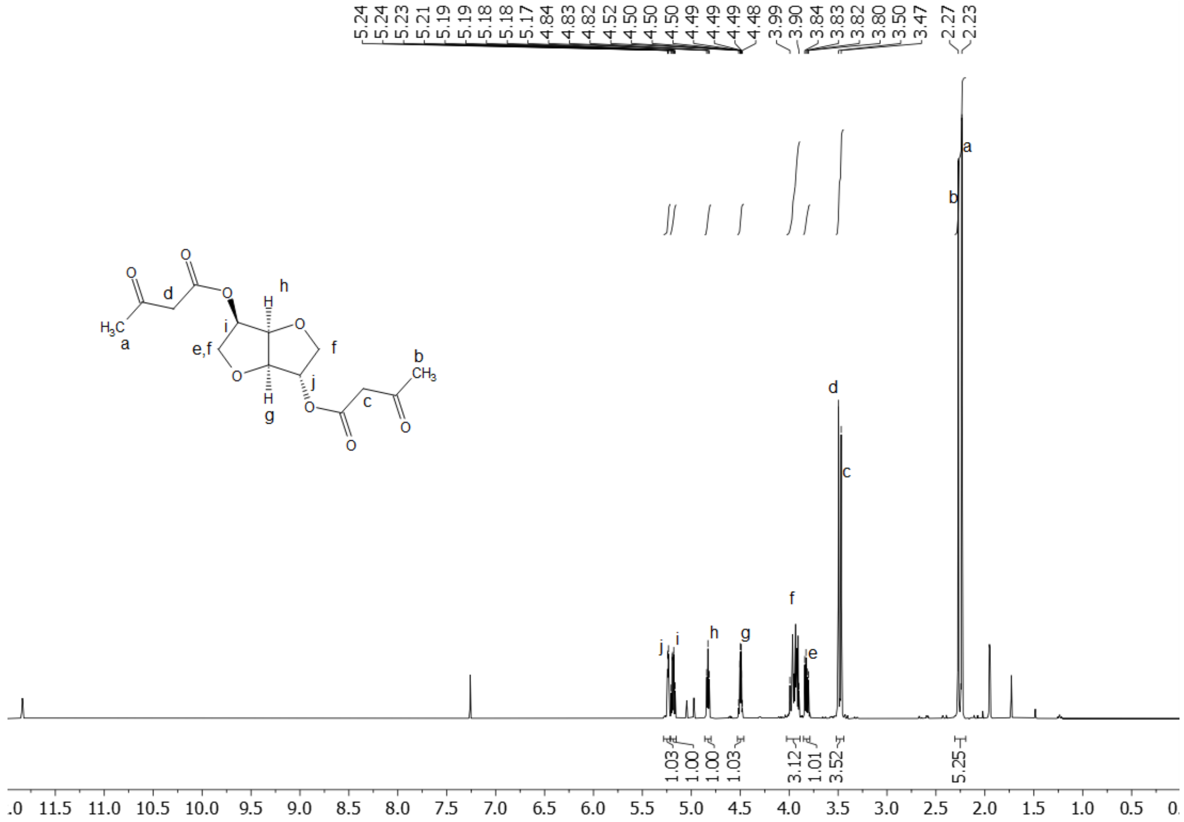

Figure S3. ^{1}H NMR spectrum of **IS-AAc** (400 MHz, 298K, $CDCl_3$). Unassigned peaks correspond to enol tautomer.

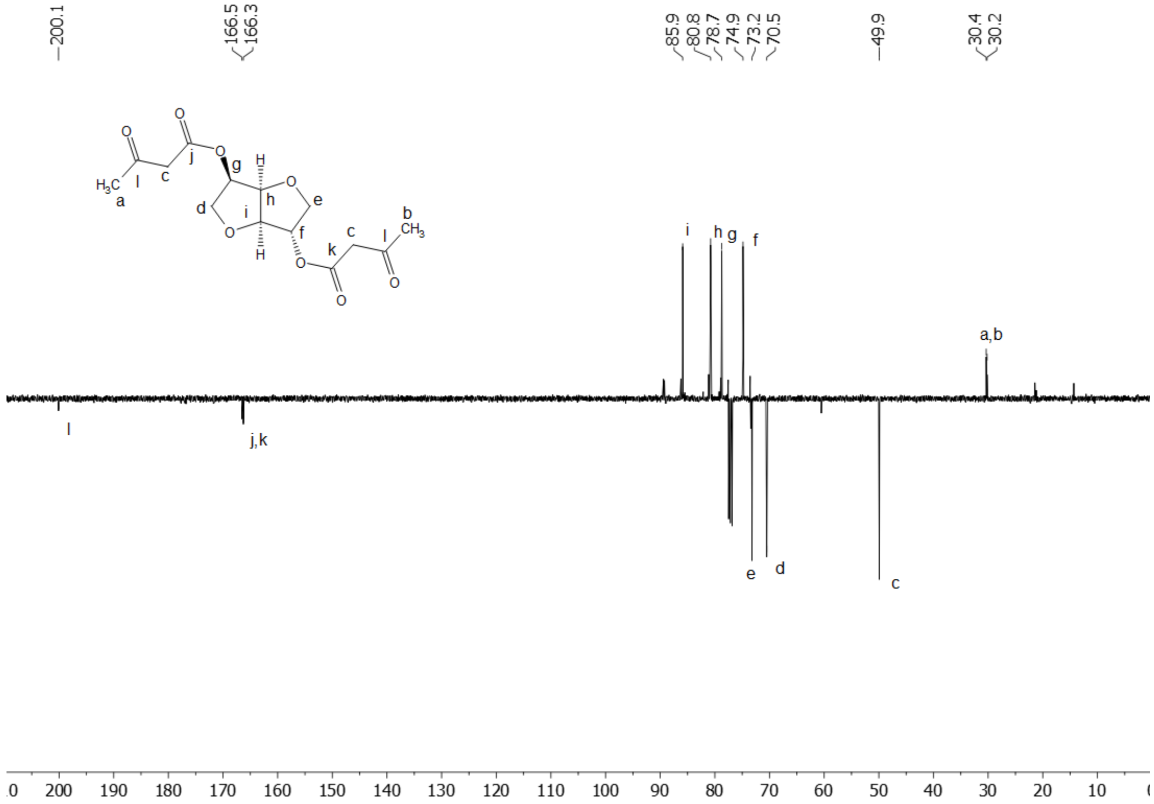

Figure S4. ^{13}C APT NMR spectrum of **IS-AAc** (100 MHz, 298 K, $CDCl_3$).

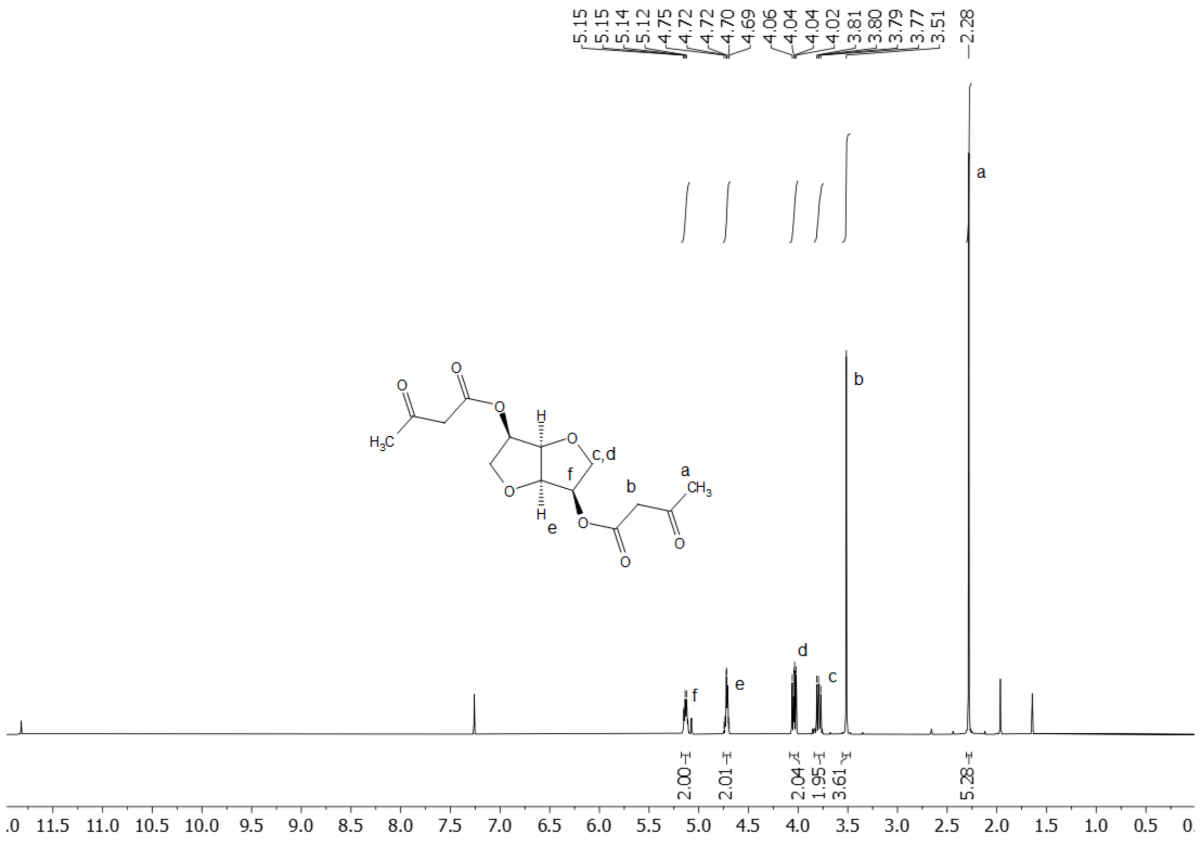

Figure S5. ^{1}H NMR spectrum of **IM-AAc** (400 MHz, 298K, $CDCl_3$). Unassigned peaks correspond to enol tautomer.

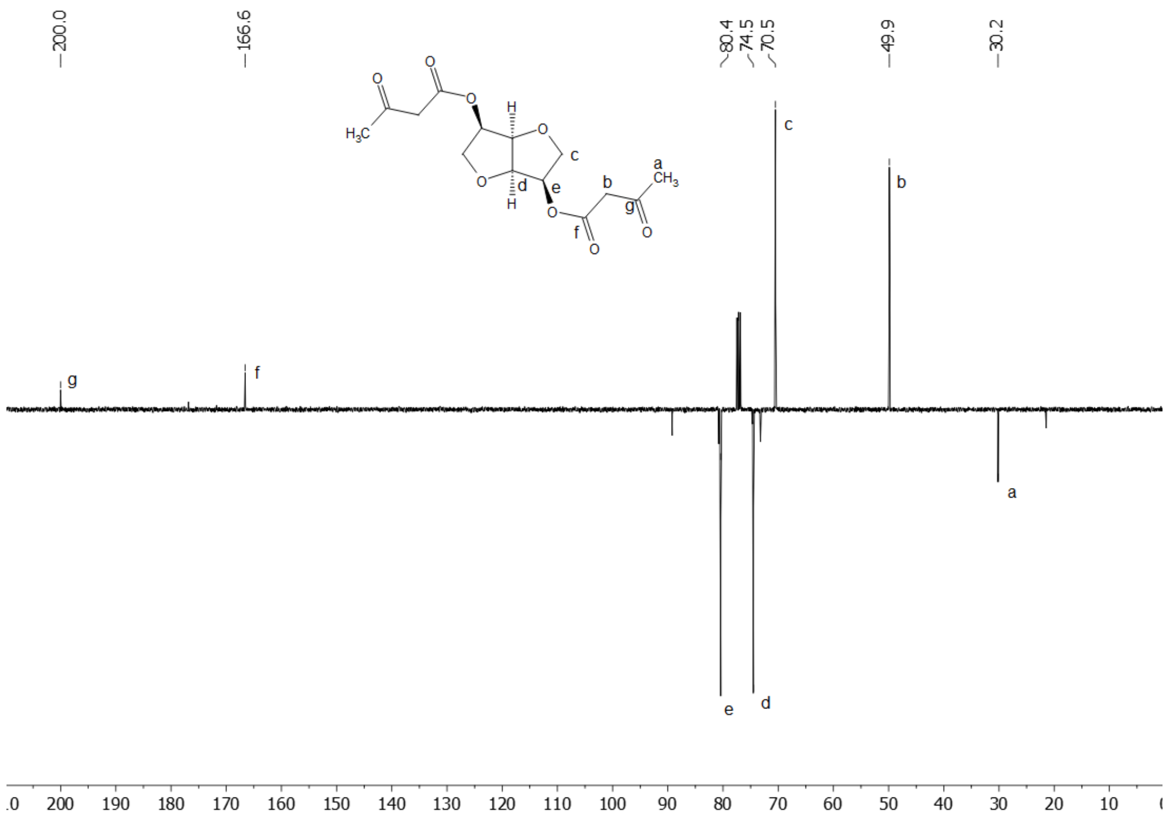

Figure S6. ^{13}C APT NMR spectrum of **IM-AAc** (100 MHz, 298 K, $CDCl_3$).

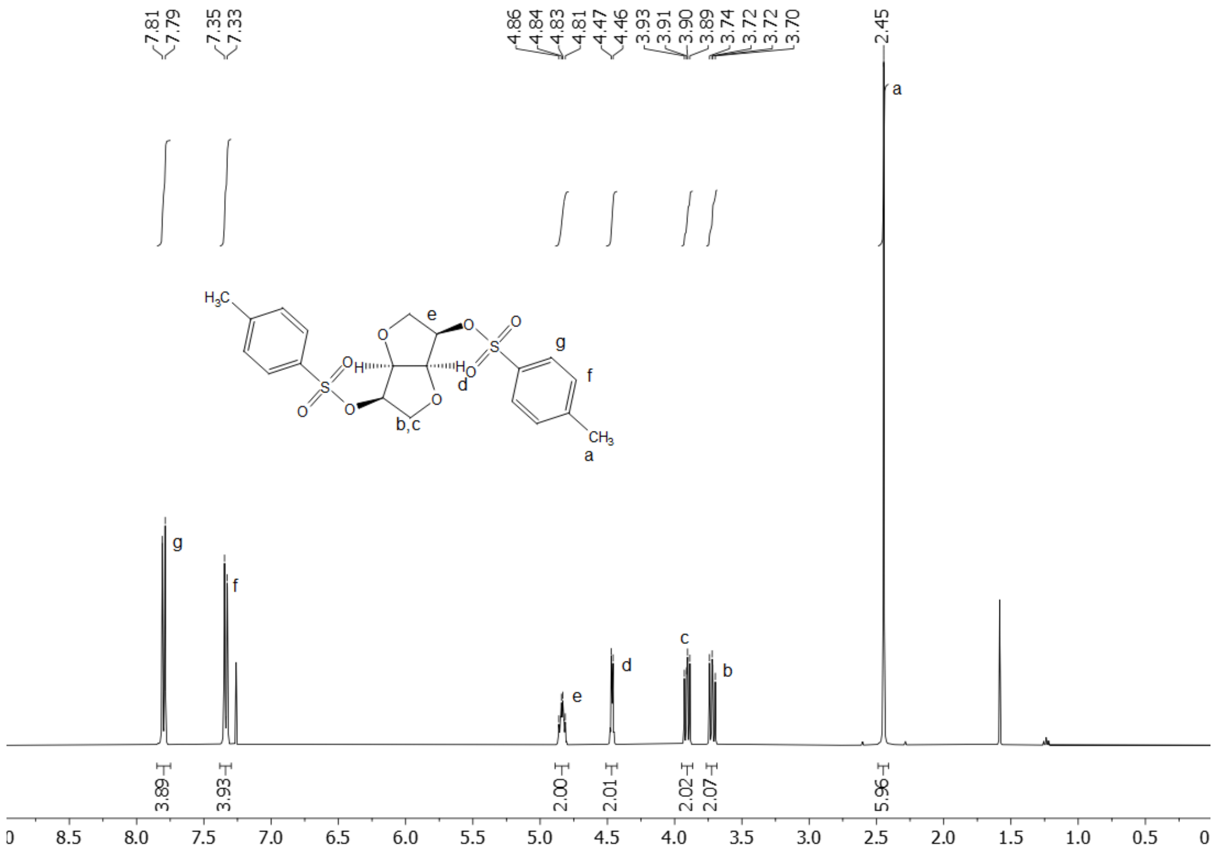

Figure S7. ^{1}H NMR spectrum of **Di-tosyl isomannide** (400 MHz, 298K, $CDCl_3$).

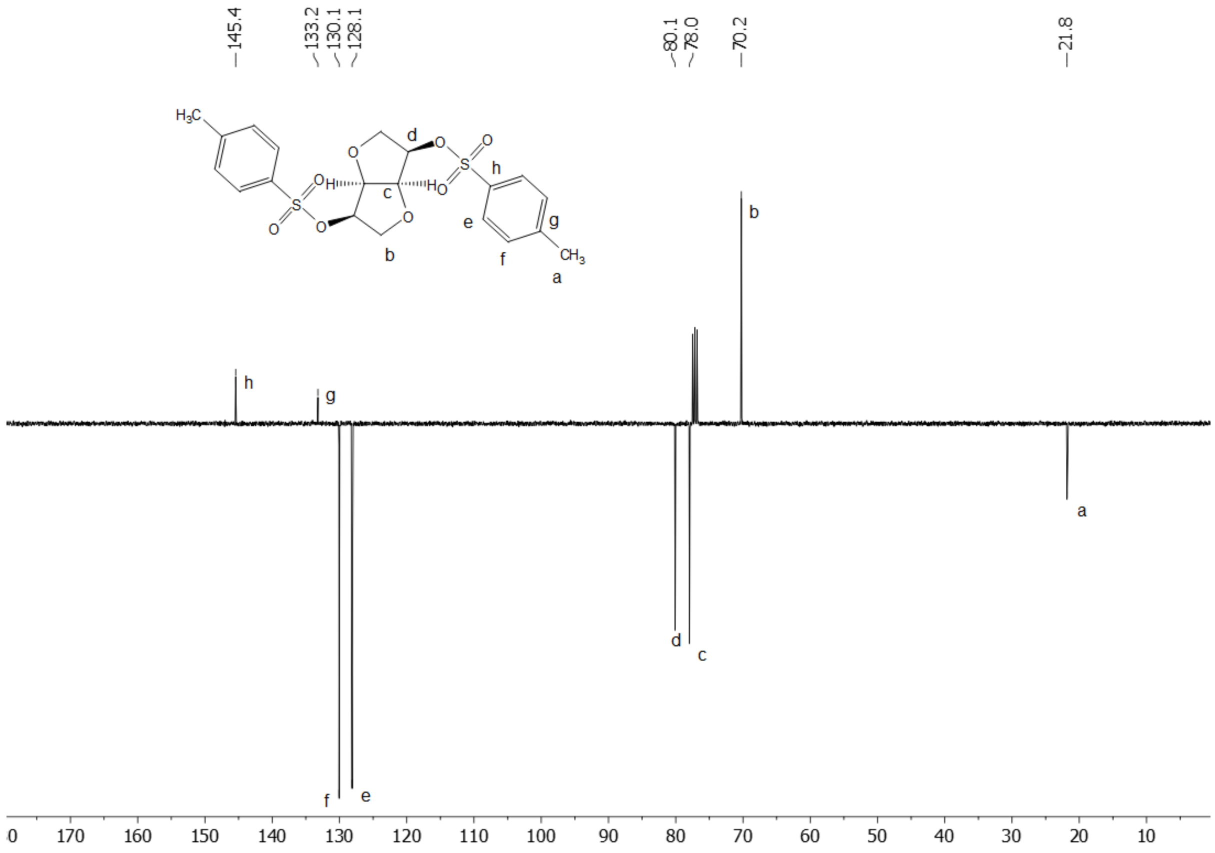

Figure S8. ^{13}C APT NMR spectrum of **Di-tosyl isomannide** (100 MHz, 298 K, $CDCl_3$).

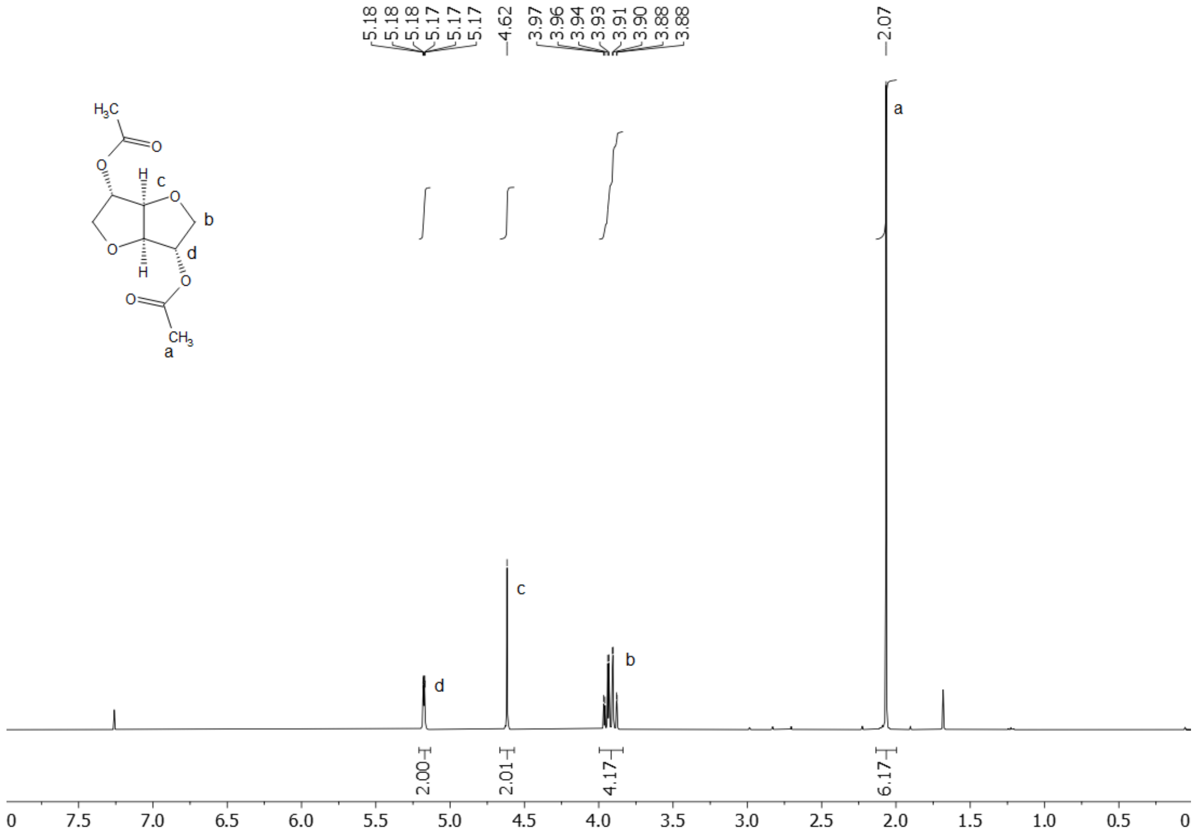

Figure S9. ^{1}H NMR spectrum of **Di-acetyl isoidide** (400 MHz, 298 K, $CDCl_3$).

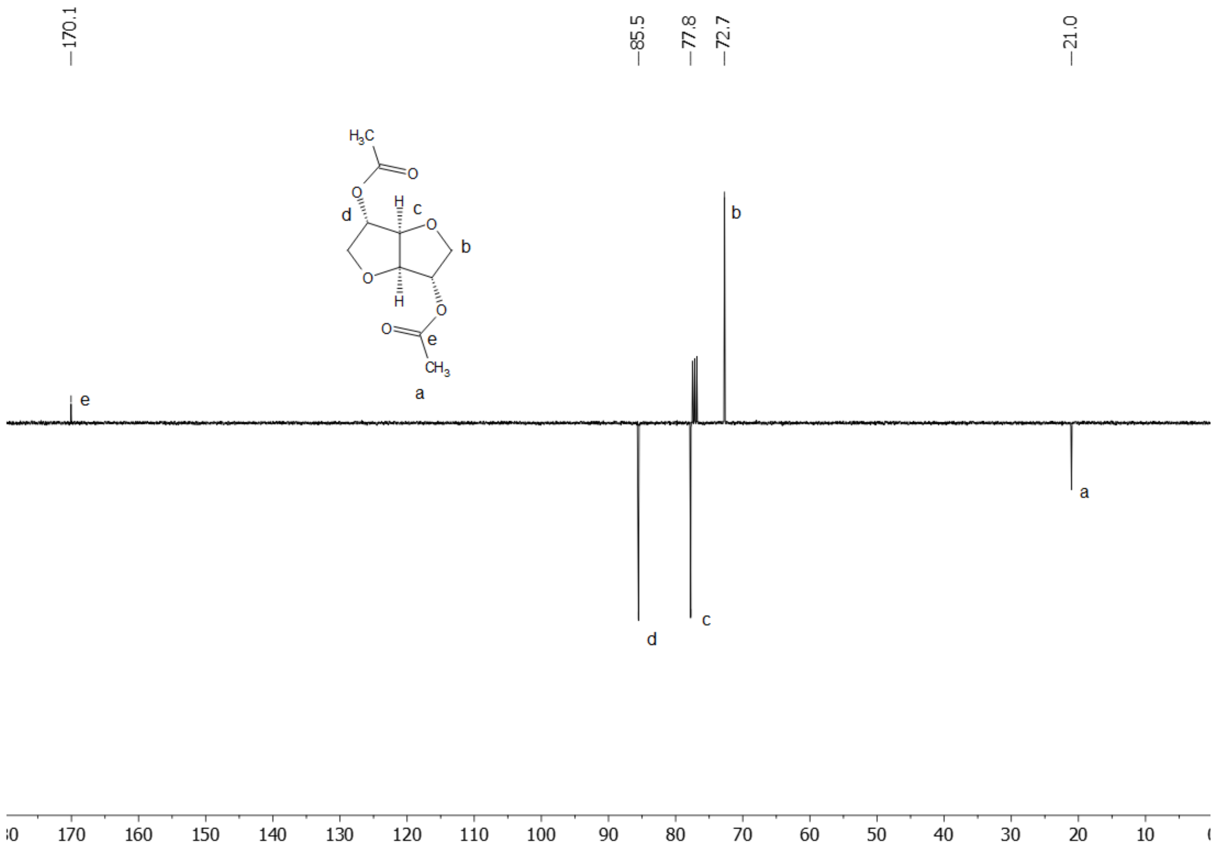

Figure S10. ^{13}C APT NMR spectrum of **Di-acetyl isoidide** (100 MHz, 298 K, $CDCl_3$).

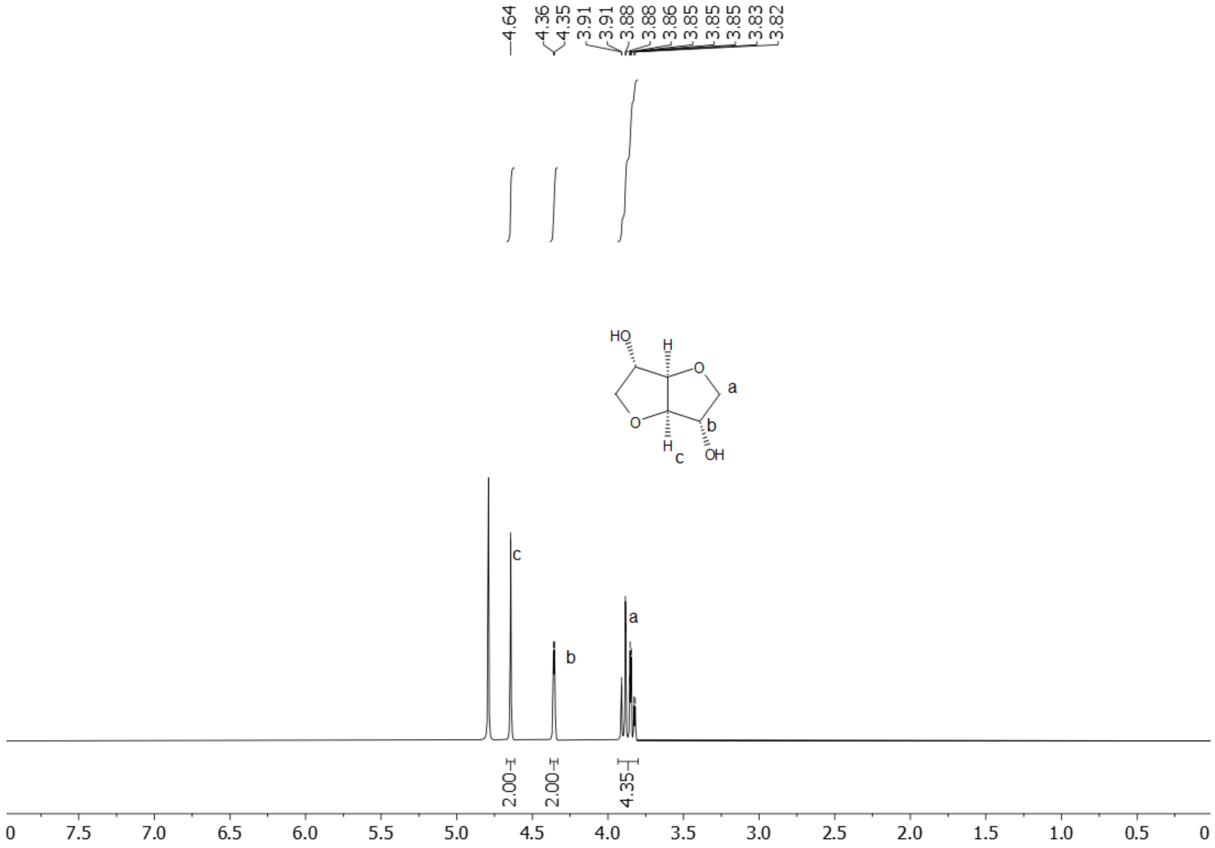

Figure S11. ^{1}H NMR spectrum of **Isoidide** (400 MHz, 298 K, D_2O).

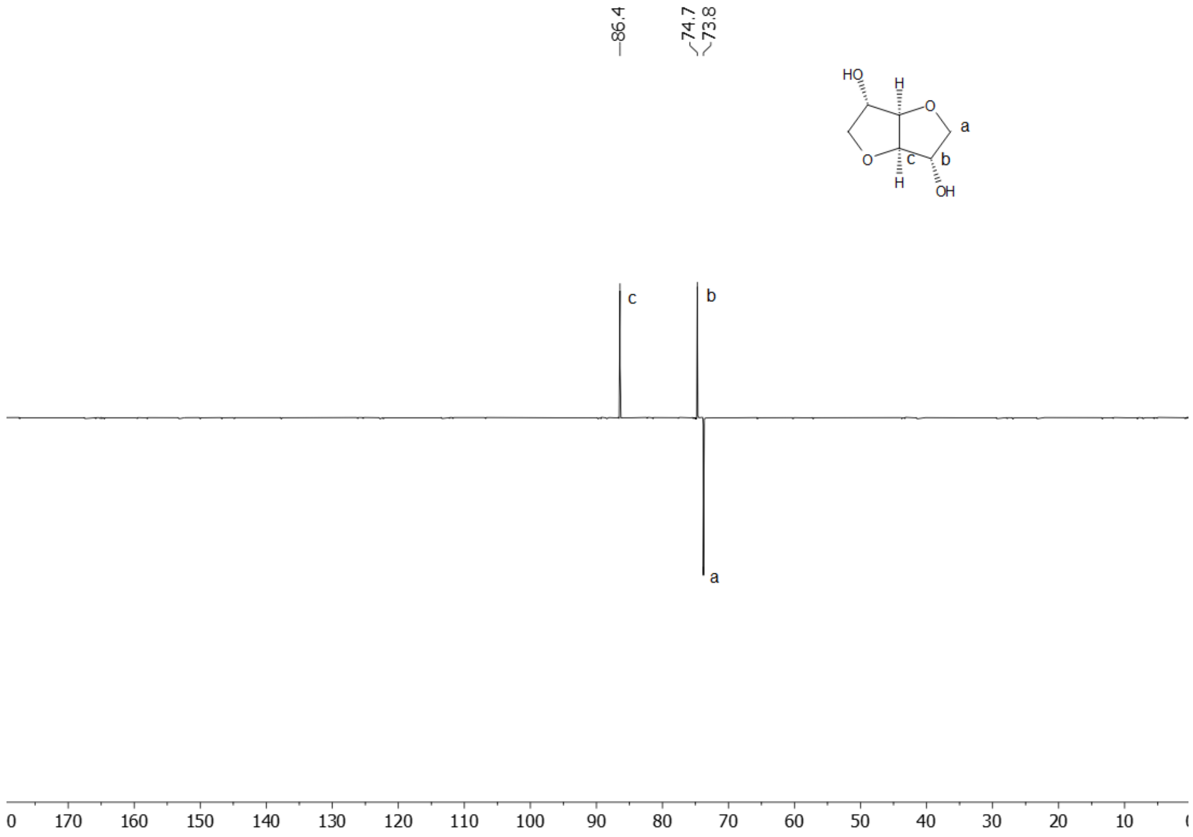

Figure S12. ^{13}C APT NMR spectrum of **Isoidide** (100 MHz, 298 K, D_2O).

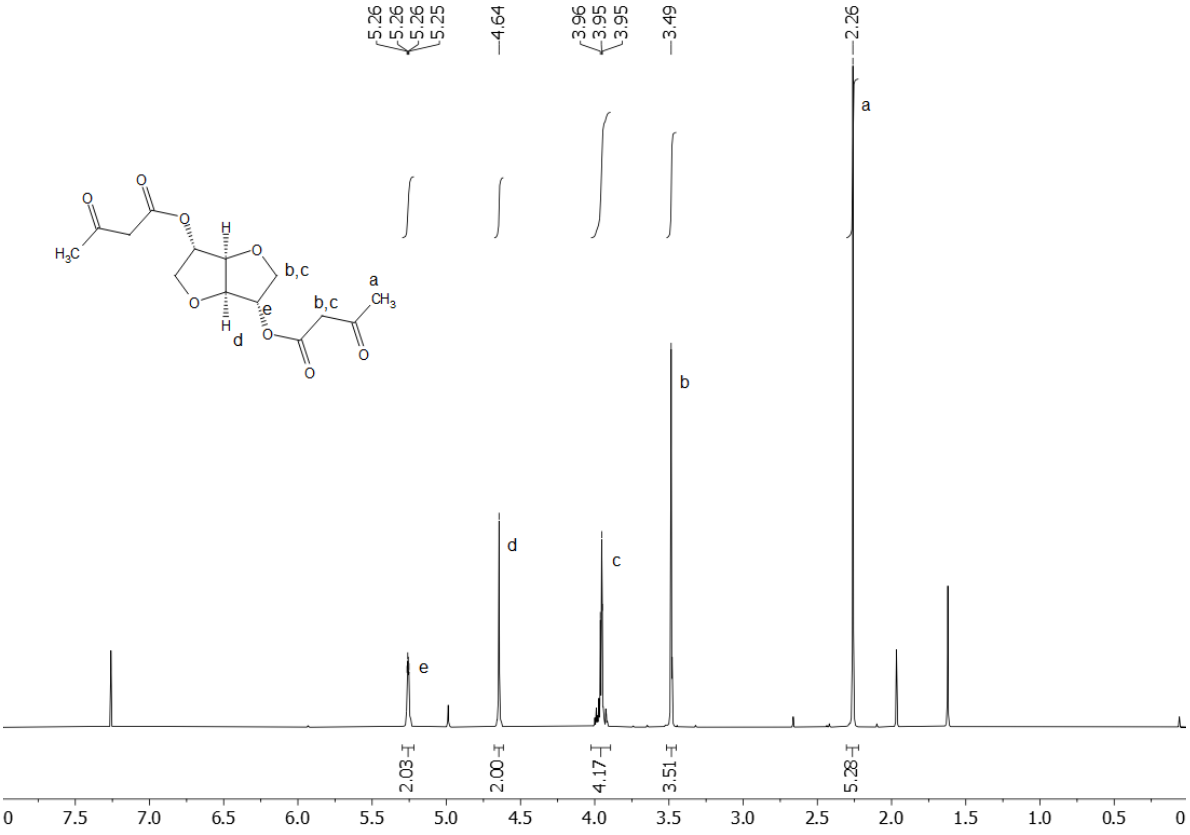

Figure S13. ^{1}H NMR spectrum of **II-AAc** (400 MHz, 298K, $CDCl_3$). Unassigned peaks correspond to enol tautomer.

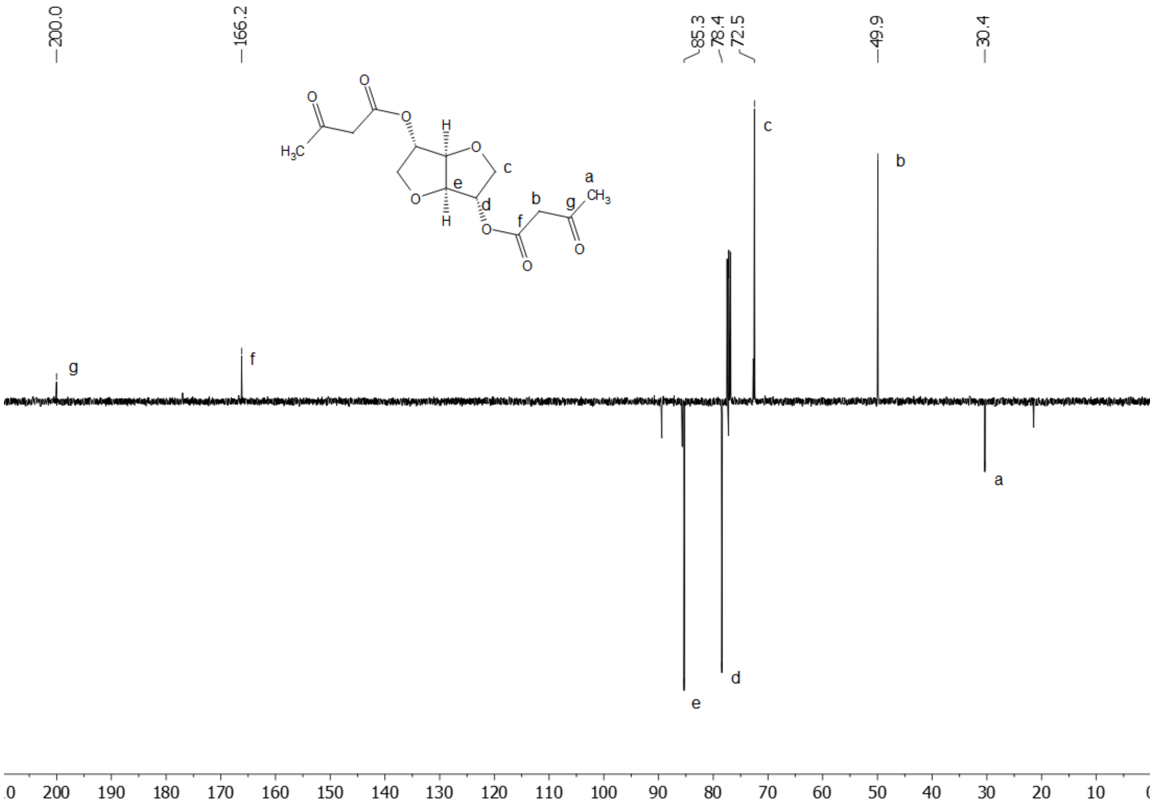

Figure S14. ^{13}C APT NMR spectrum of **IM-AAc** (100 MHz, 298 K, $CDCl_3$).

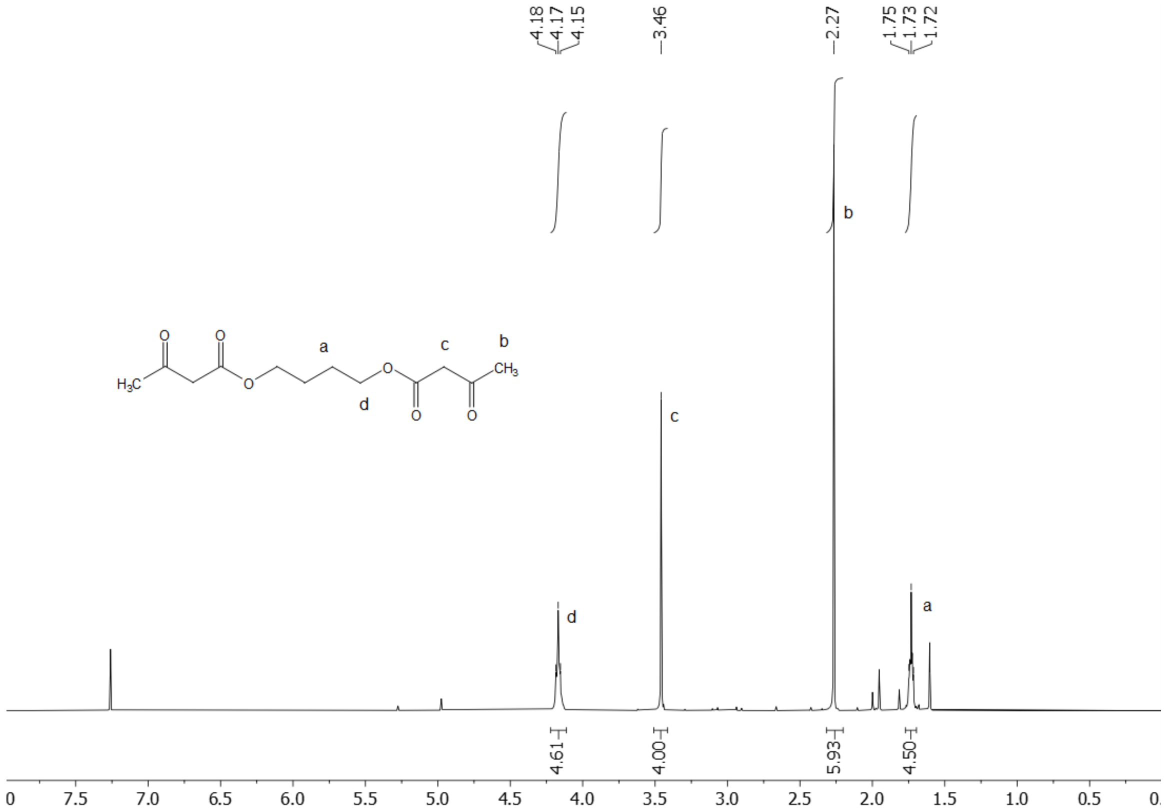

Figure S15. ^{1}H NMR spectrum of **1,4-BD-AAc** (400 MHz, 298K, $CDCl_3$). Unassigned peaks correspond to enol tautomer.

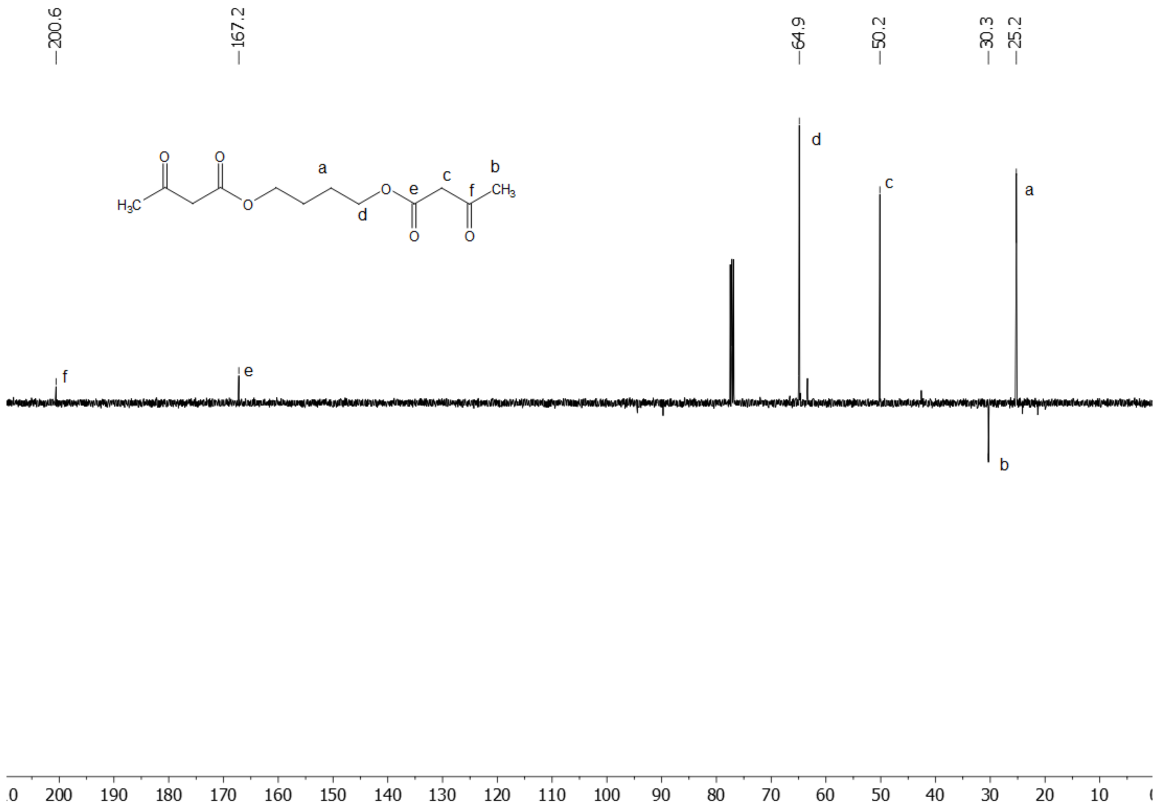

Figure S16. ^{13}C APT NMR spectrum of **1,4-BD-AAc** (100 MHz, 298 K, $CDCl_3$).

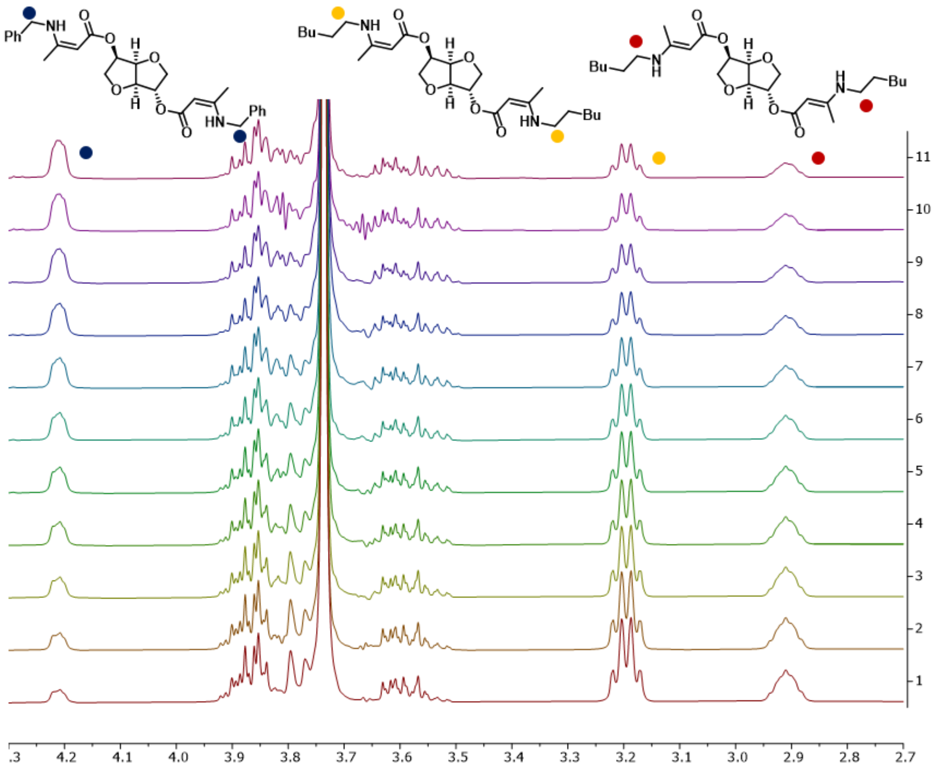

Figure S17. ^{1}H NMR spectra of IS-Hx-VU exchange with Bn-NH_2 at T = 80 °C (400 MHz, 298 K, d_6-DMSO).

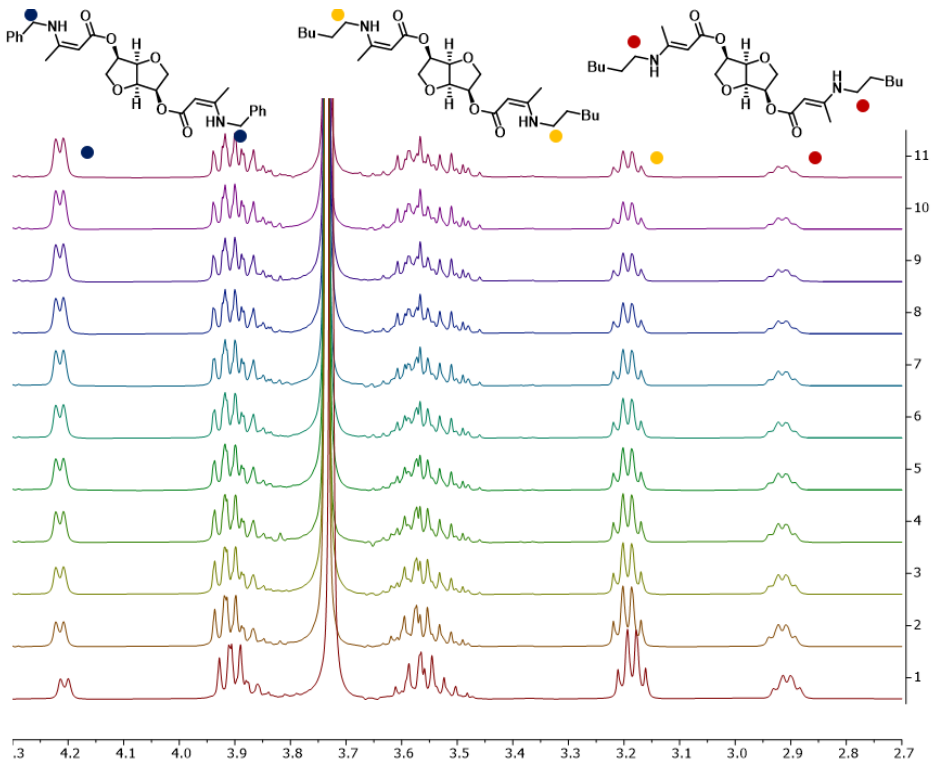

Figure S18. ^{1}H NMR spectra of IM-Hx-VU exchange with Bn-NH_2 at T = 80 °C (400 MHz, 298 K, d_6-DMSO).

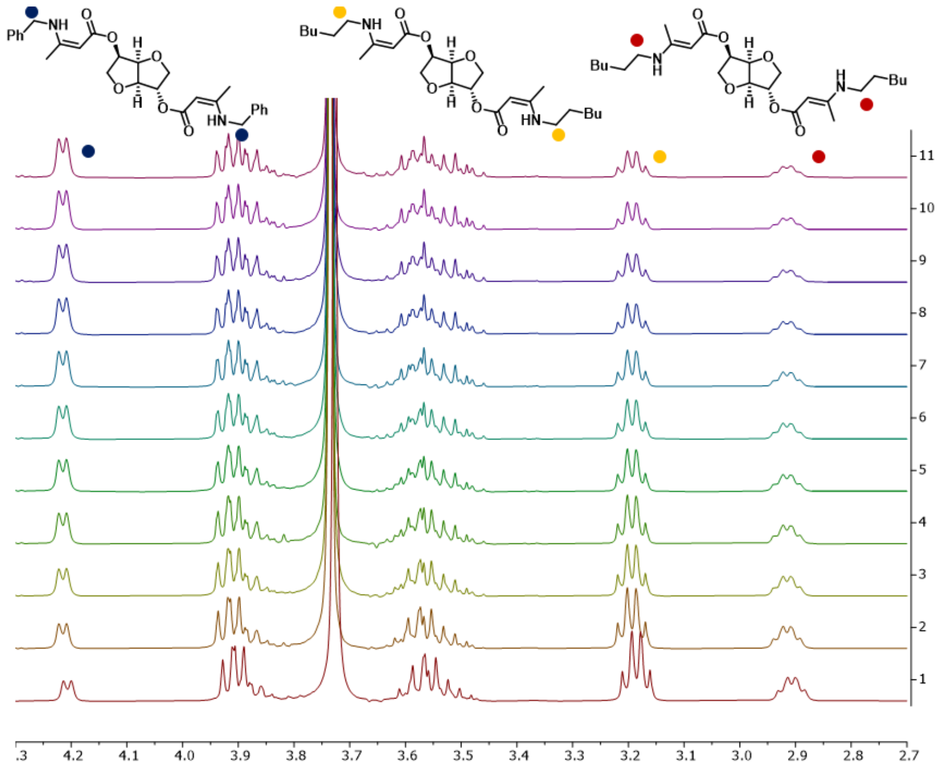

Figure S19. ^{1}H NMR spectra of IS-Hx-VU exchange with Bn-NH_2 at T = 100 °C (400 MHz, 298 K, d_6-DMSO).

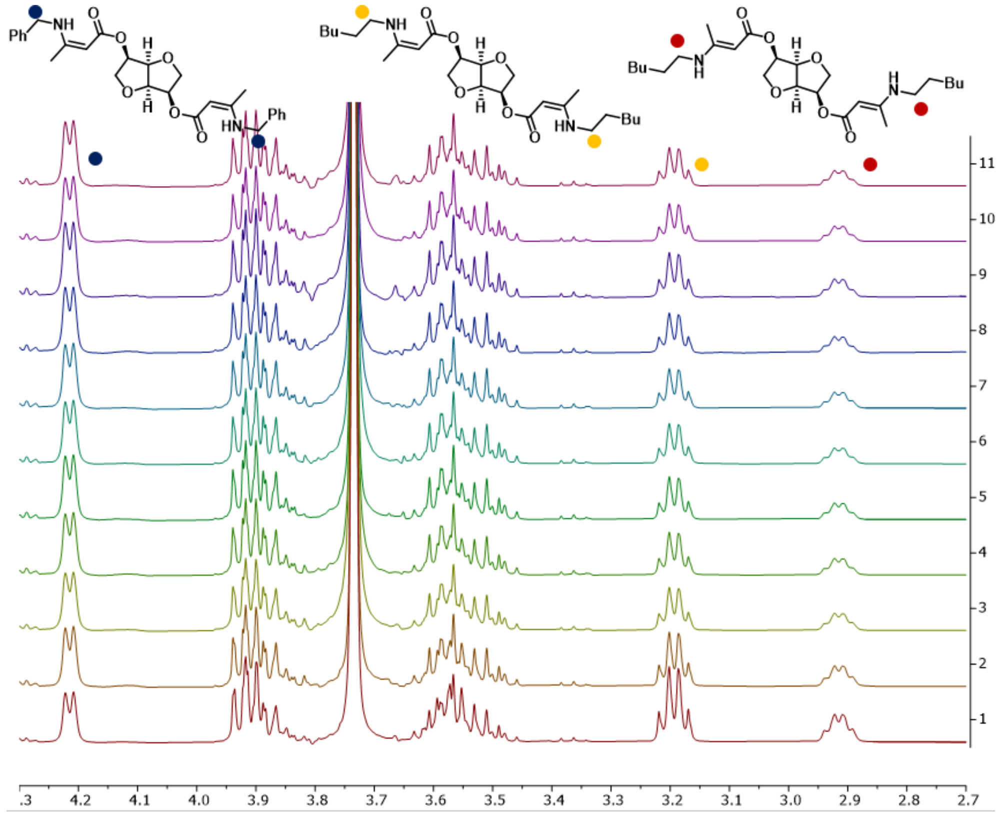

Figure S20. ^{1}H NMR spectra of IM-Hx-VU exchange with Bn-NH_2 at T = 100 °C (400 MHz, 298 K, d_6-DMSO).

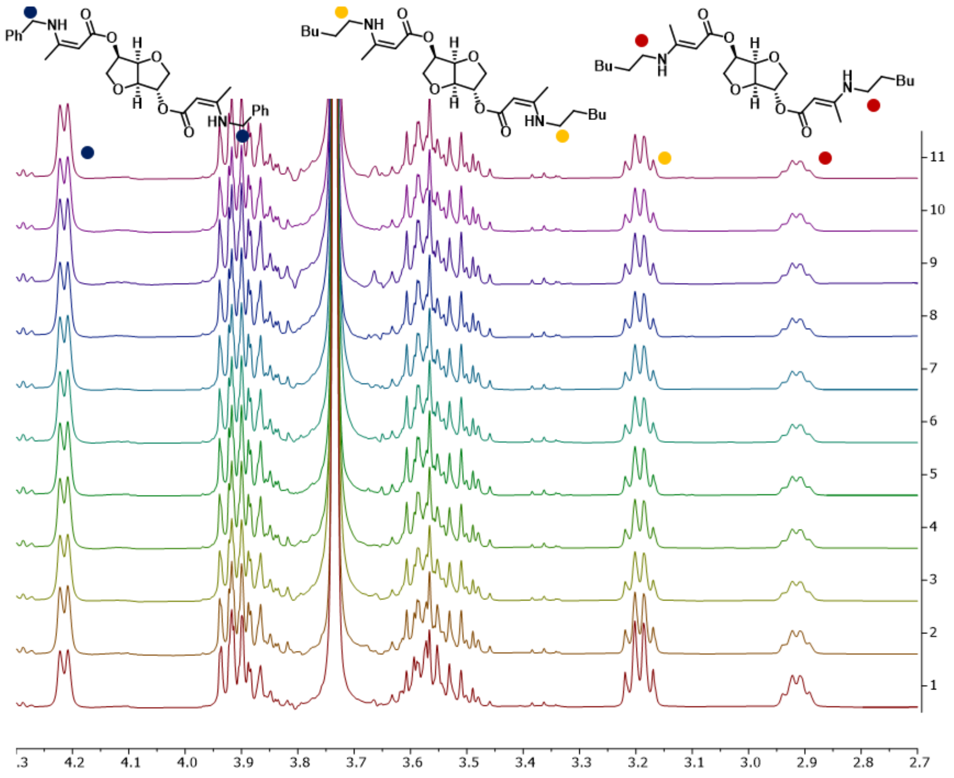

Figure S21. ^{1}H NMR spectra of IS-Hx-VU exchange with Bn-NH_2 at T = 120 °C (400 MHz, 298 K, d_6-DMSO).

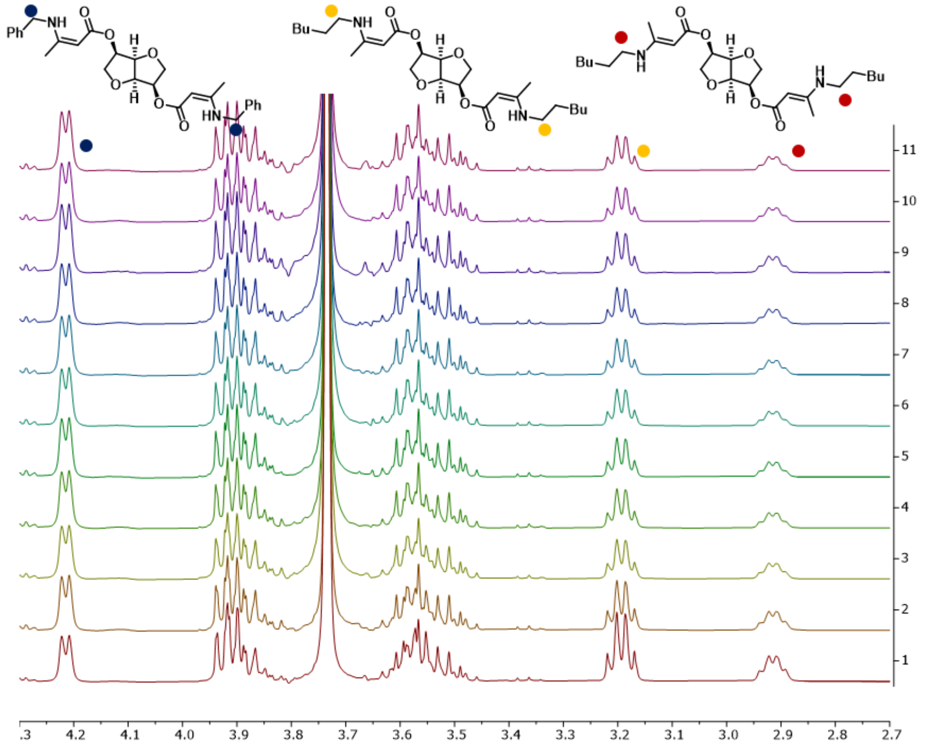

Figure S22. ^{1}H NMR spectra of IM-Hx-VU exchange with Bn-NH_2 at T = 120 °C (400 MHz, 298 K, d_6-DMSO).

Kinetic plots for the model reaction vinylogous urethane exchanges

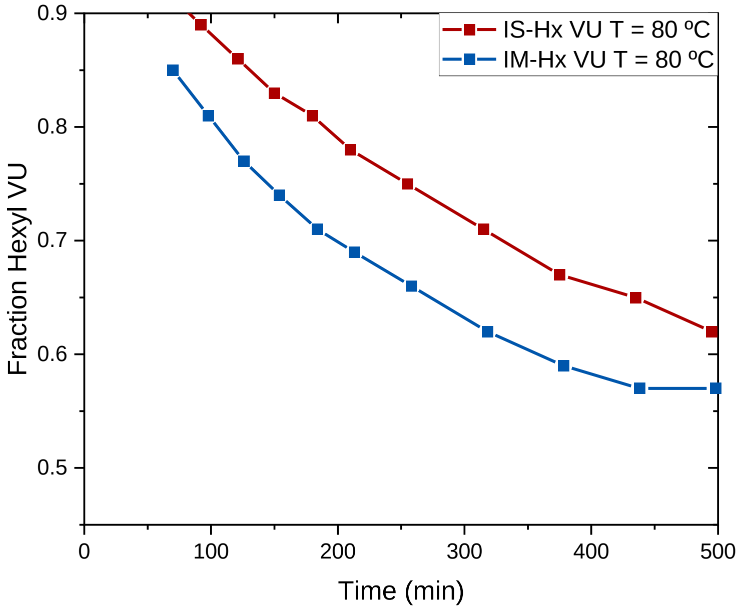

Figure S23. Decrease of IS-Hx-VU and IM-Hx-VU as a function of time at T = 80 °C when treated with an excess of Bn-NH_2.

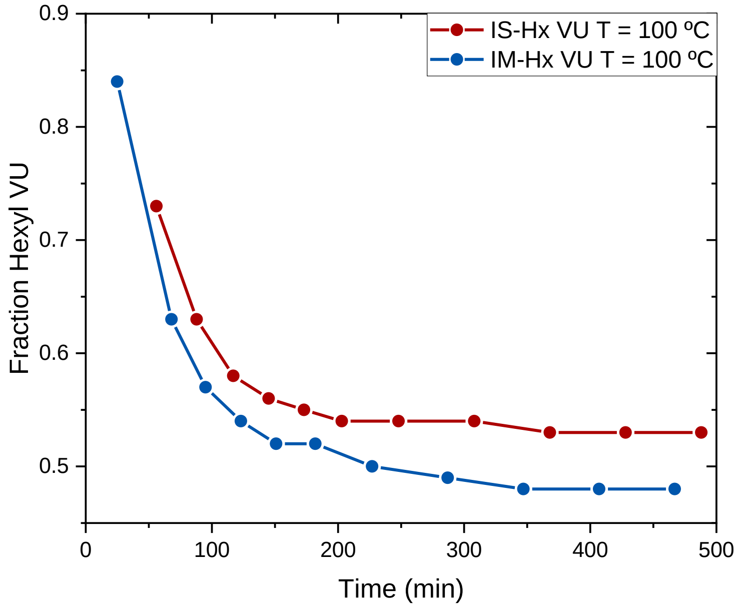

Figure S24. Decrease of IS-Hx-VU and IM-Hx-VU as a function of time at T = 100 °C when treated with an excess of Bn-NH_2.

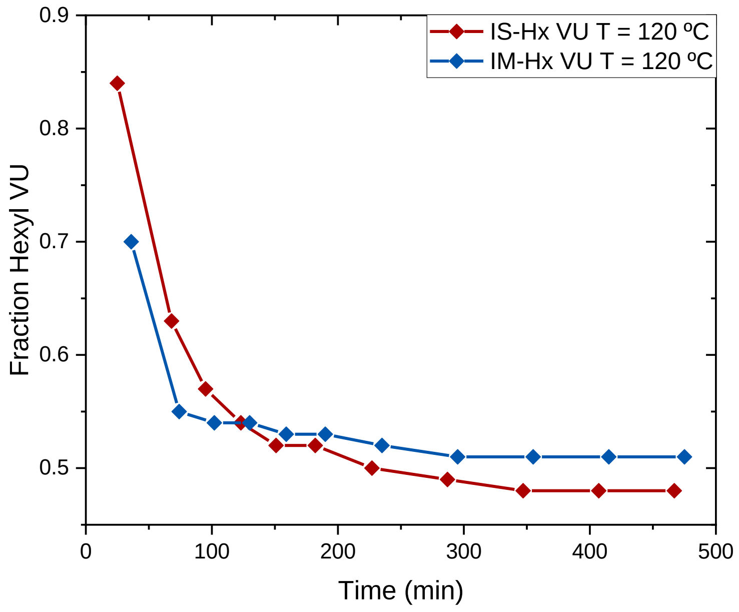

Figure S25. Decrease of IS-Hx-VU and IM-Hx-VU as a function of time at T = 120 °C when treated with an excess of Bn-NH_2.

Arrhenius plots for the model reaction vinylogous urethane exchanges

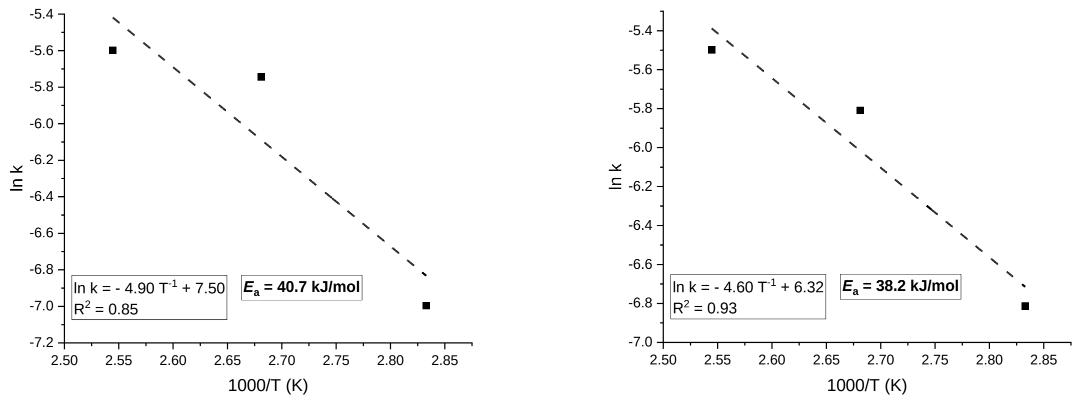

Figure S26. Arrhenius plot on the exchange reaction of IS-Hx-VU with Bn-NH_2 (*left*) and Arrhenius plot on the exchange reaction of IM-Hx-VU with Bn-NH_2 (*right*).

FTIR

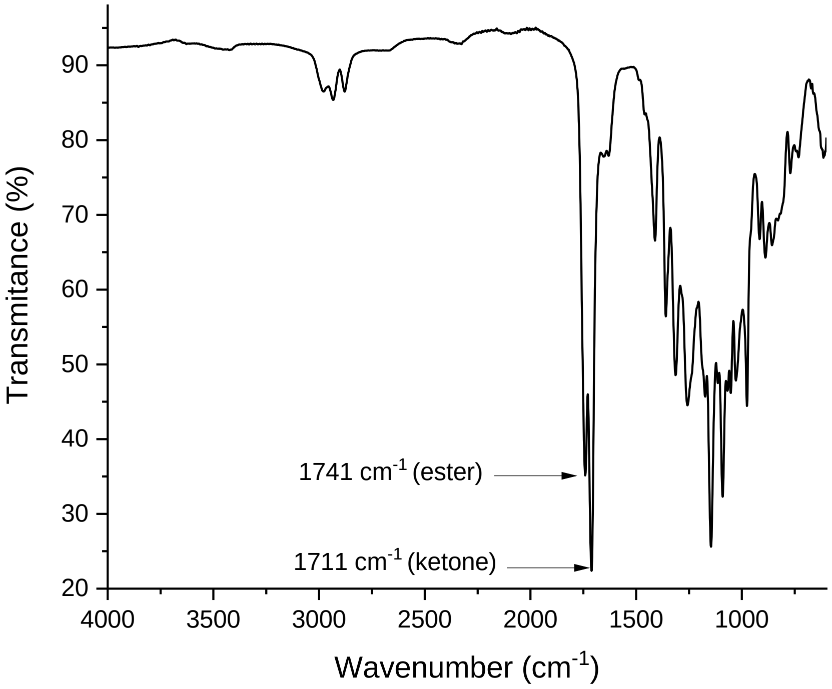

Figure S27. FTIR spectrum of monomer **IS-AAc**.

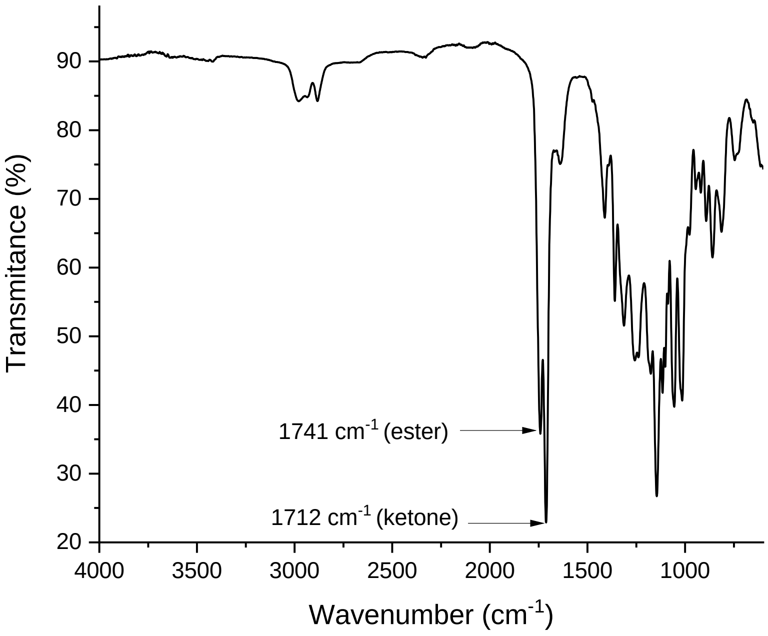

Figure S28. FTIR spectrum of monomer **IM-AAc**.

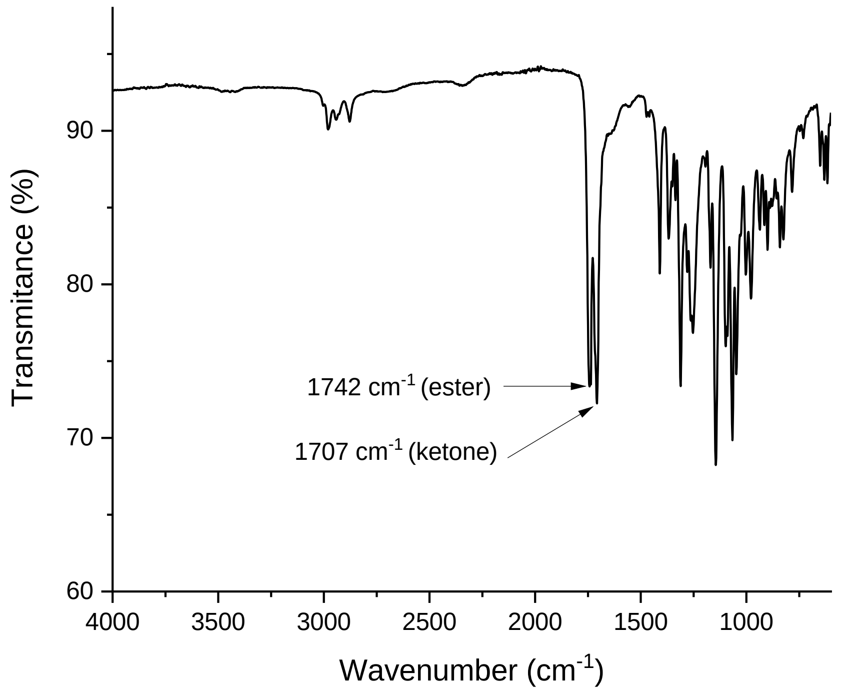

Figure S29. FTIR spectrum of monomer **II-AAc**.

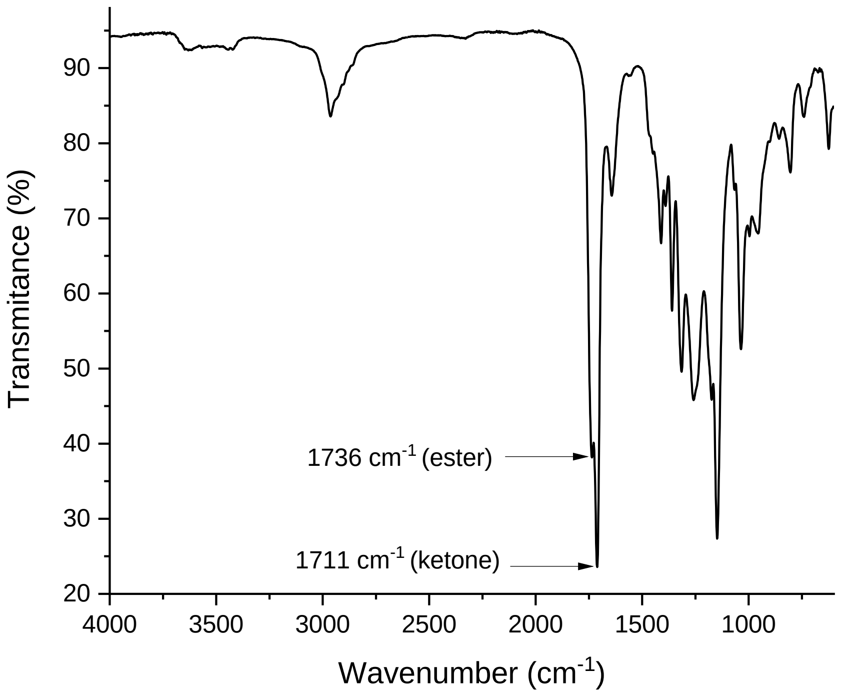

Figure S30. FTIR spectrum of monomer **1,4-BD-AAc**.

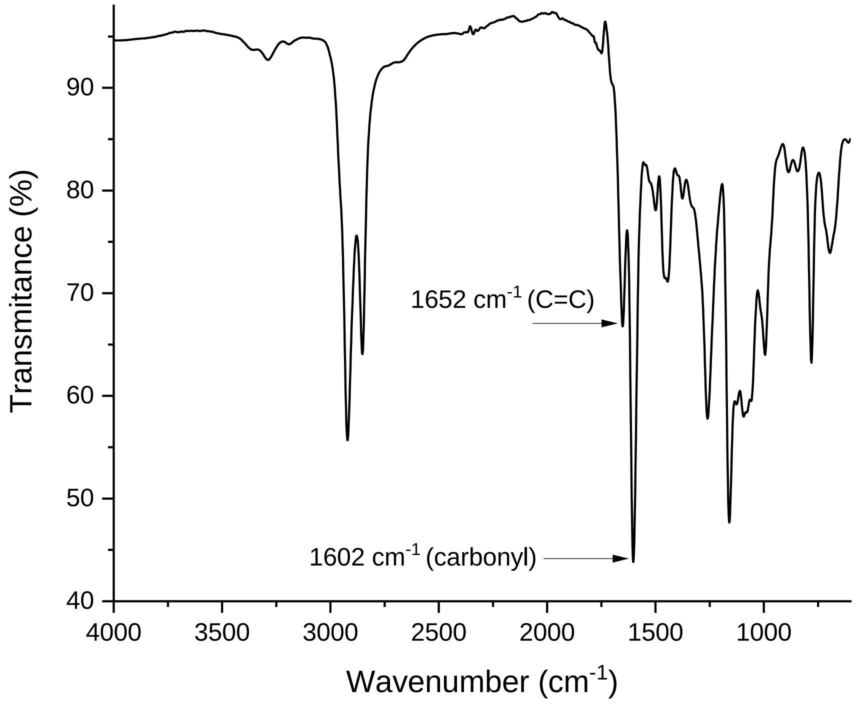

Figure S31. FTIR spectrum of network **IS-Pri** after curing process 140 °C during 8 h.

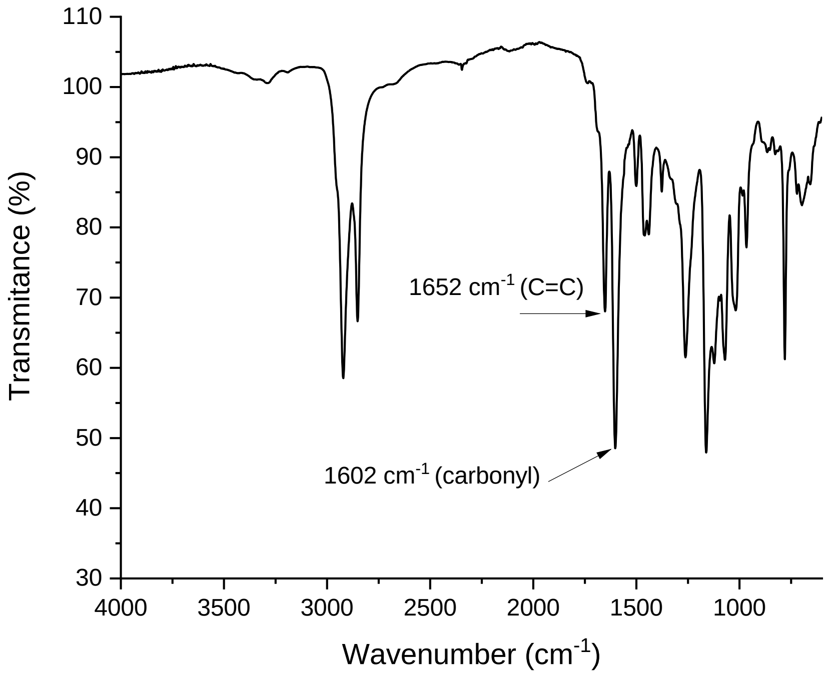

Figure S32. FTIR spectrum of network **IM-Pri** after curing process 140 °C during 8 h.

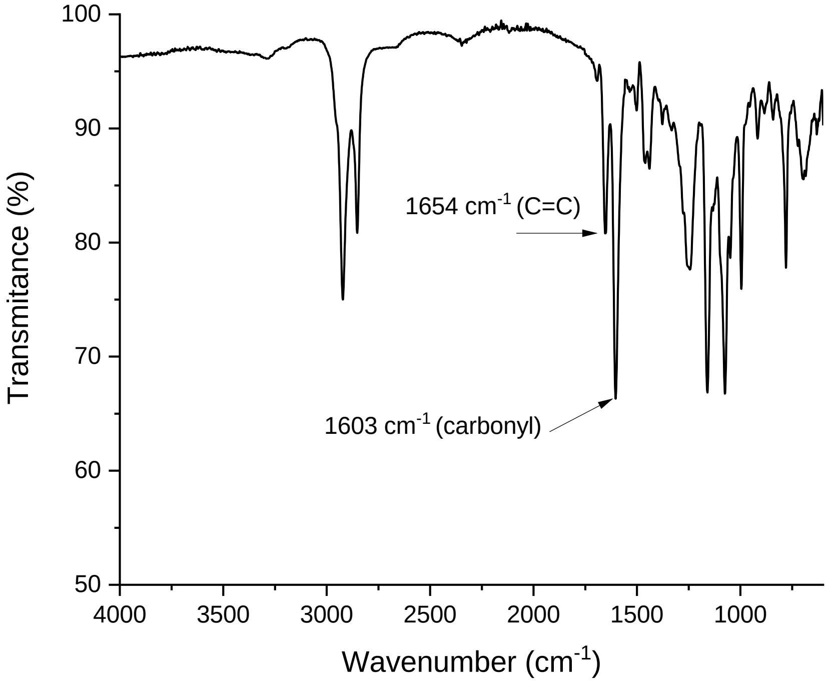

Figure S33. FTIR spectrum of network **II-Pri** after curing process 140 °C during 8 h.

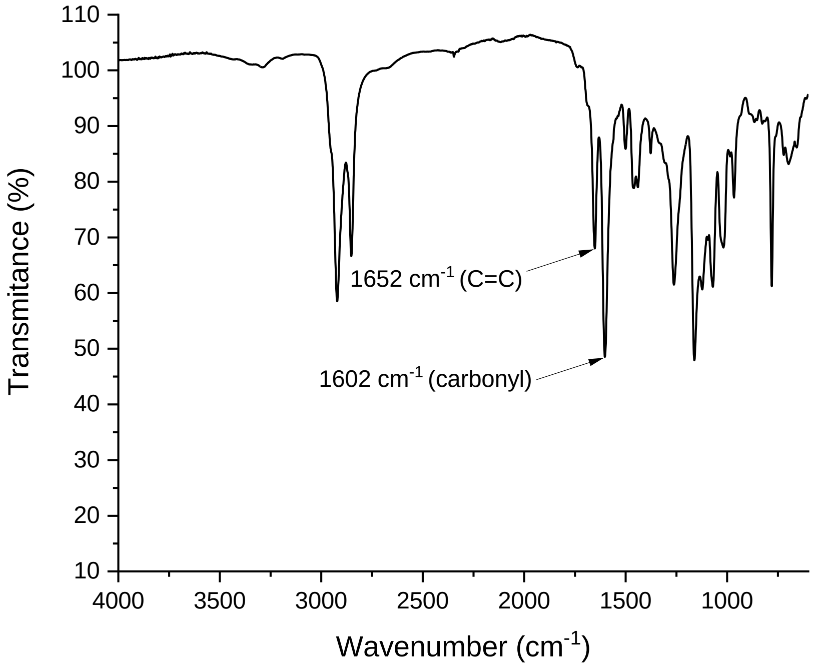

Figure S34. FTIR spectrum of network **IM$_{0.5}$-II$_{0.5}$-Pri** after curing process 140 °C during 8 h.

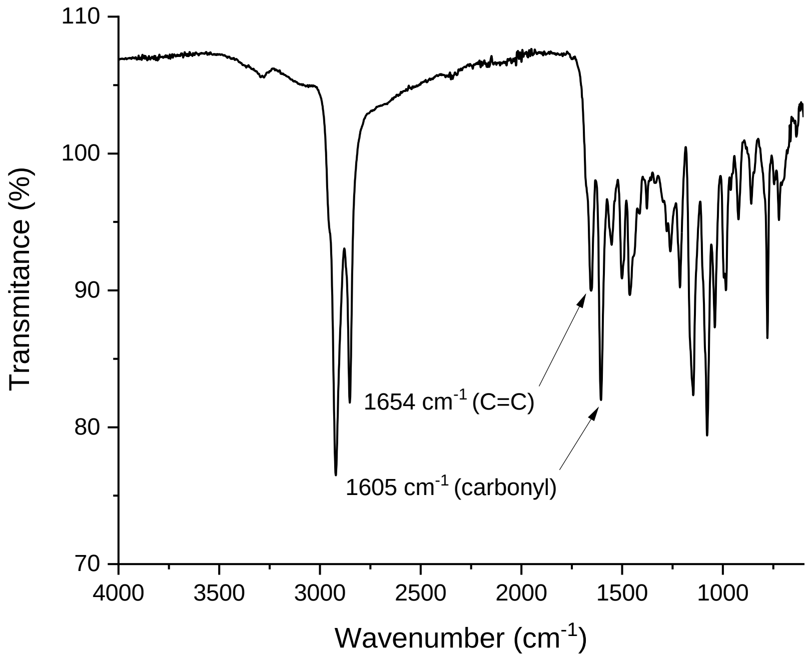

Figure S35. FTIR spectrum of network **IS-Pri$_1$-Jeff$_2$** after curing process 140 °C during 8 h.

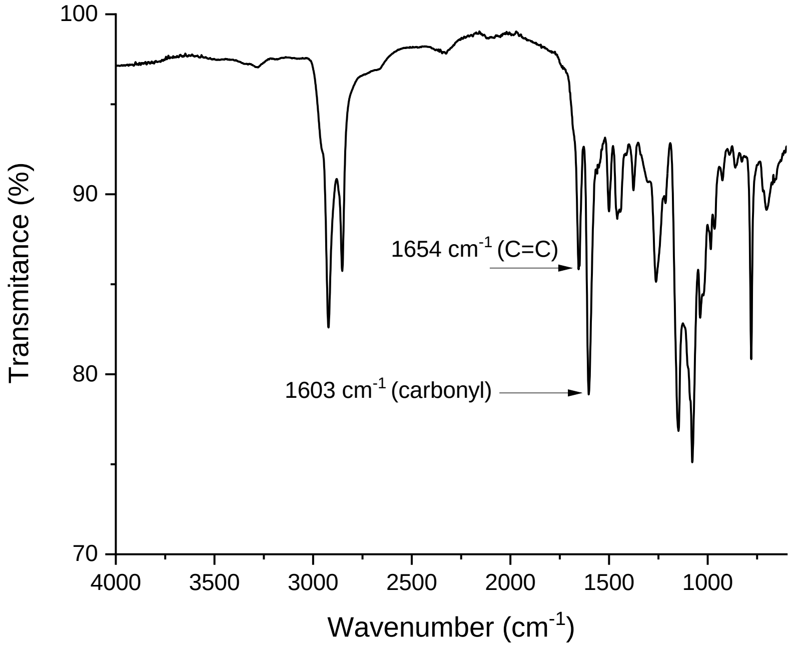

Figure S36. FTIR spectrum of network **IM-Pri$_1$-Jeff$_2$** after curing process 140 °C during 8 h.

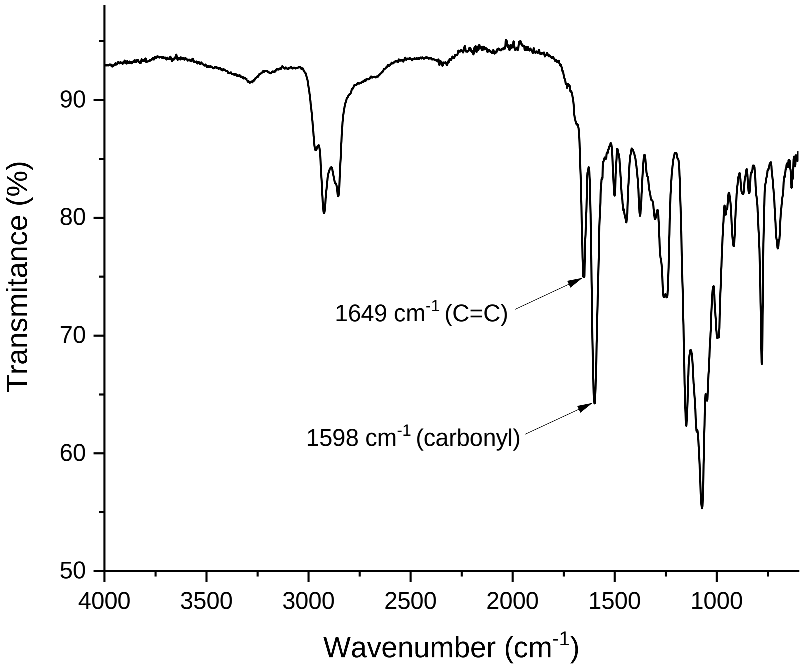

Figure S37. FTIR spectrum of network **II-Pri$_1$-Jeff$_2$** after curing process 140 °C during 8 h.

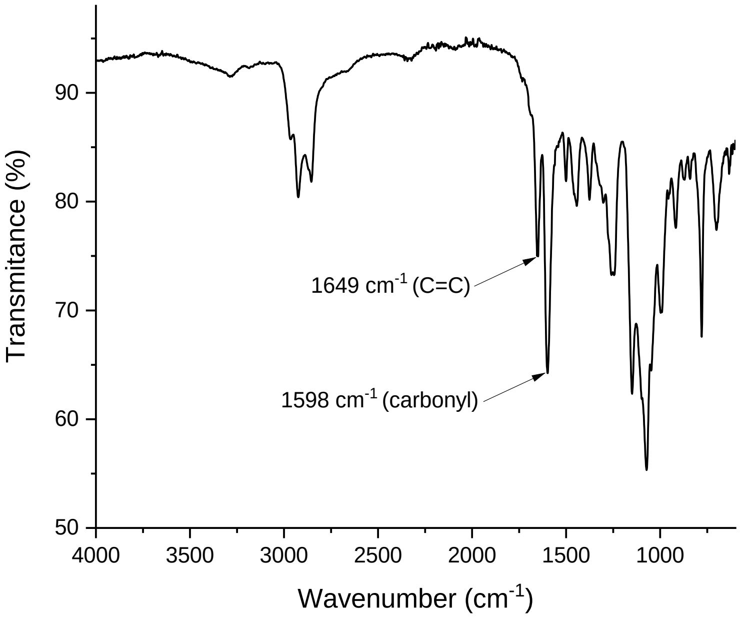

Figure S38. FTIR spectrum of network **IM$_{0.5}$-II$_{0.5}$-Pri$_1$-Jeff$_2$** after curing process 140 °C during 8 h.

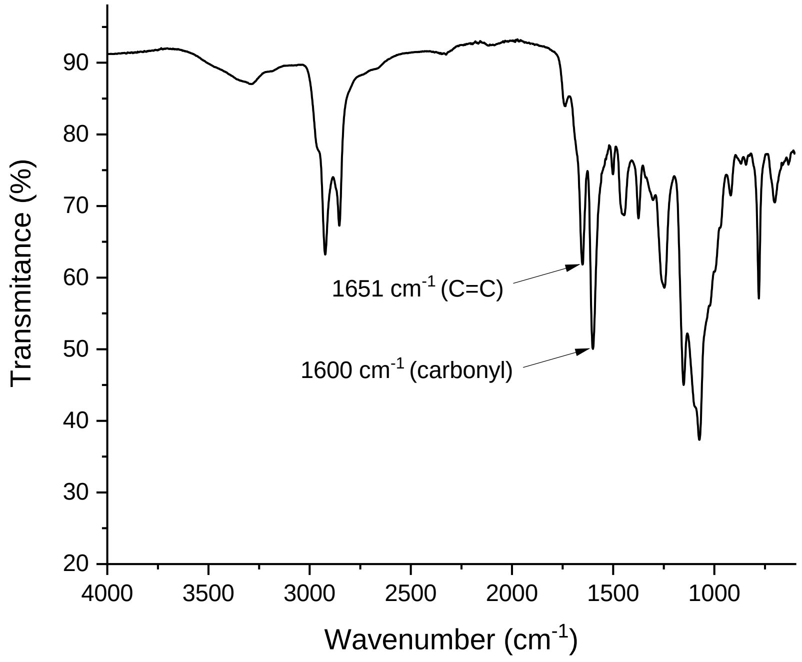

Figure S39. FTIR spectrum of network **IS-Jeff** after curing process 140 °C during 8 h.

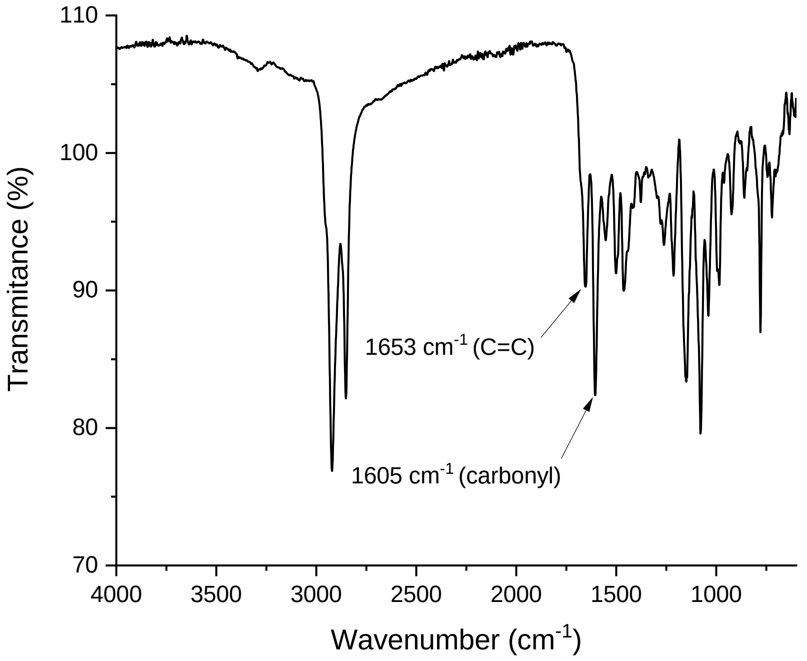

Figure S40. FTIR spectrum of network **IM-Jeff** after curing process 140 °C during 8 h.

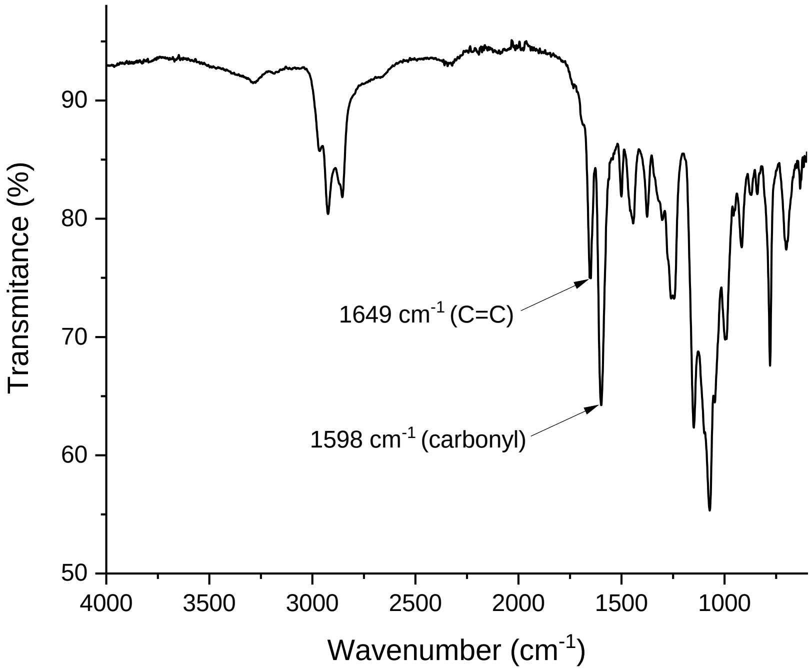

Figure S41. FTIR spectrum of network **II-Jeff** after curing process 140 °C during 8 h.

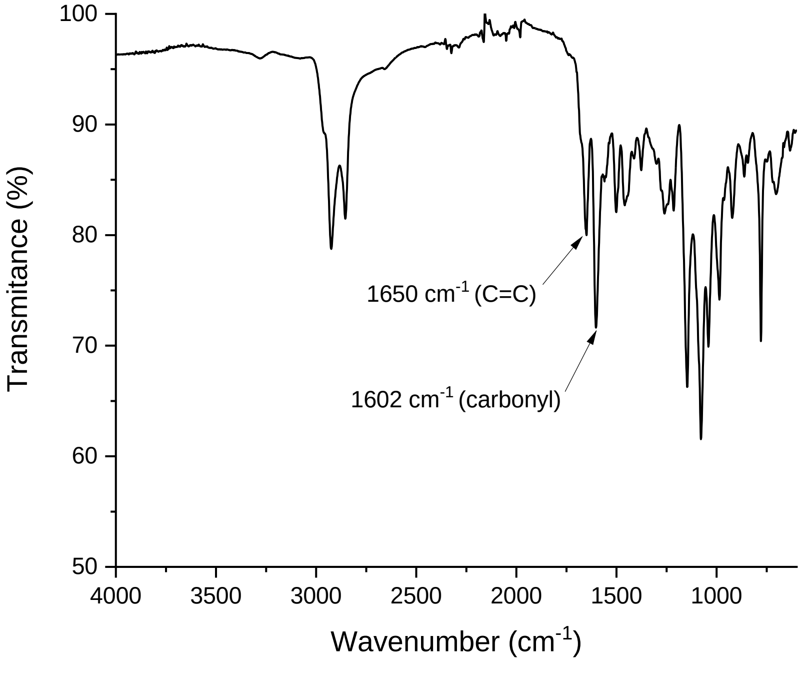

Figure S42. FTIR spectrum of network **IM$_{0.5}$-II$_{0.5}$-Jeff** after curing process 140 °C during 8 h.

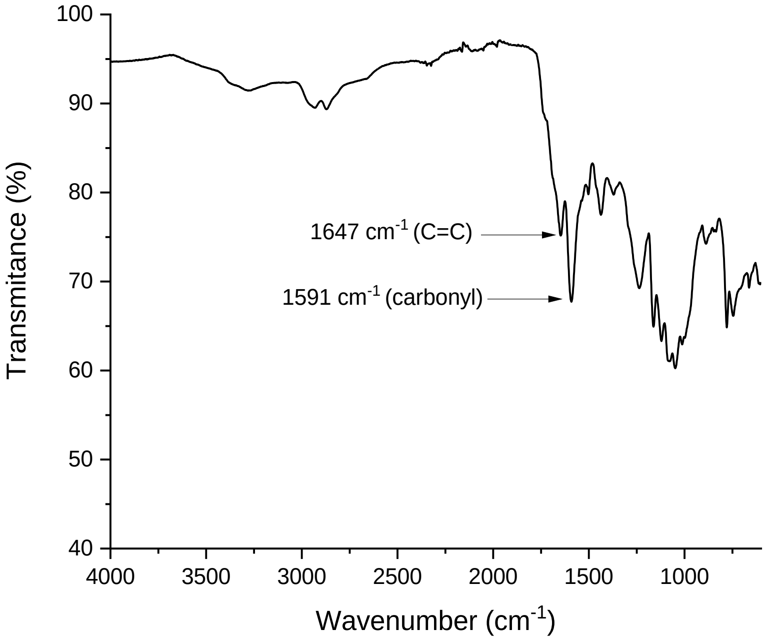

Figure S43. FTIR spectrum of network **IS-TAEA** after curing process 140 °C during 8 h.

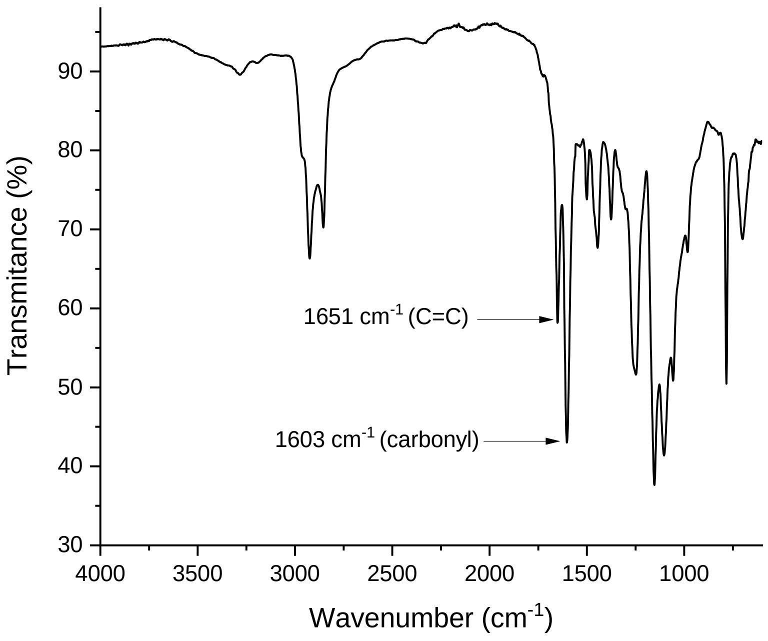

Figure S44. FTIR spectrum of network **BD-Pri$_1$-Jeff$_2$** after curing process 140 °C during 8 h.

Gel content experiments

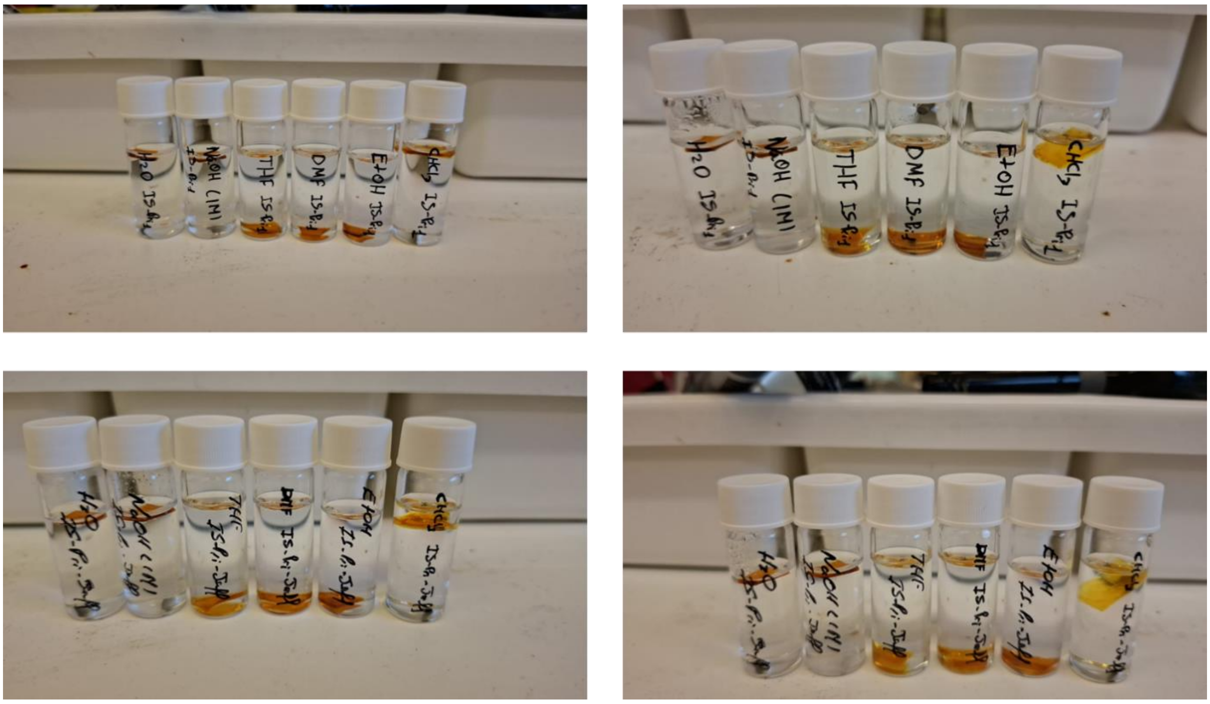

Figure S45. Gel content experiments for **IS-Pri** (*left*) and **IS-Pri$_1$-Jeff$_2$** (*right*). Pictures on the top correspond to t = 0 and pictures at the bottom correspond to t = 24 h.

Table S1. Gel content after immersion at r.t in different solvents of IH-based vinylogous CANs.

Entry	CAN	H_2O (%)	$NaOH_{(aq)}$ (%)	THF (%)	DMF (%)	EtOH (%)	$CHCl_3$ (%)
1	IS-Pri	99 ± 1	99 ± 1	84 ± 1	93 ± 4	99 ± 1	84 ± 1
2	IM-Pri	99 ± 1	99 ± 1	87 ± 1	95 ± 1	95 ± 1	87 ± 1
3	II-Pri	99 ± 1	98 ± 1	88 ± 1	92 ± 1	95 ± 1	87 ± 1
4	II$_{0.5}$-IM$_{0.5}$-Pri	99 ± 1	99 ± 1	87 ± 1	96 ± 1	97 ± 1	88 ± 1
5	IS-Pri$_1$-Jeff$_2$	99 ± 1	99 ± 1	89 ± 1	90 ± 1	95 ± 1	90 ± 1
6	IM-Pri$_1$-Jeff$_2$	99 ± 1	99 ± 1	88 ± 1	89 ± 1	92 ± 1	89 ± 1
7	II-Pri$_1$-Jeff$_2$	99 ± 1	96 ± 4	92 ± 1	96 ± 1	97 ± 2	89 ± 1
8	II$_{0.5}$-IM$_{0.5}$-Pri$_1$-Jeff$_2$	99 ± 1	99 ± 1	93 ± 1	95 ± 1	97 ± 1	91 ± 1
9	IS-Jeff	99 ± 1	99 ± 1	91 ± 1	92 ± 1	98 ± 2	91 ± 1
10	IM-Jeff	99 ± 1	99 ± 1	94 ± 1	95 ± 2	99 ± 1	88 ± 1
11	II-Jeff	99 ± 1	99 ± 1	95 ± 1	94 ± 1	98 ± 1	90 ± 2
12	II$_{0.5}$-IM$_{0.5}$-Jeff	99 ± 1	99 ± 1	96 ± 1	95 ± 1	98 ± 1	89 ± 3

TGA results

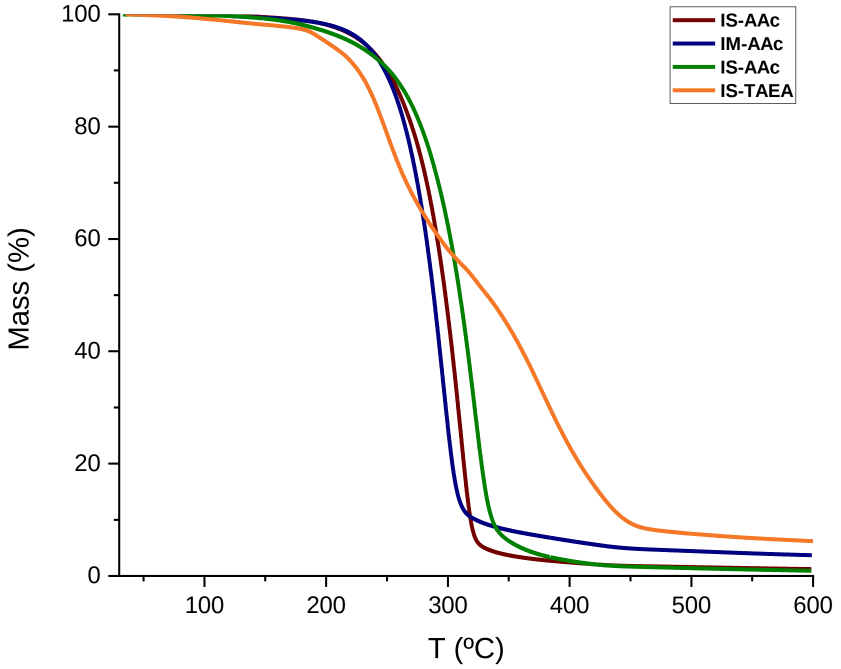

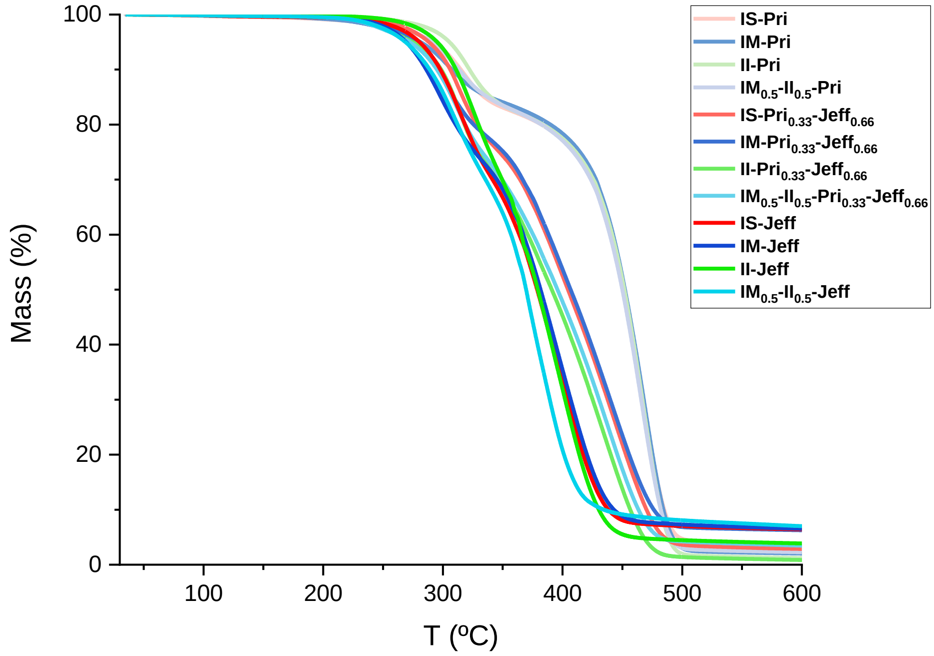

Figure S46. TGA curves of monomers **IS-AAc**, **IM-AAc**, **II-AAc**, **IS-TAEA** (*top*) and CANs **IH-Pri$_x$-Jeff$_y$** (*bottom*).

DSC analysis

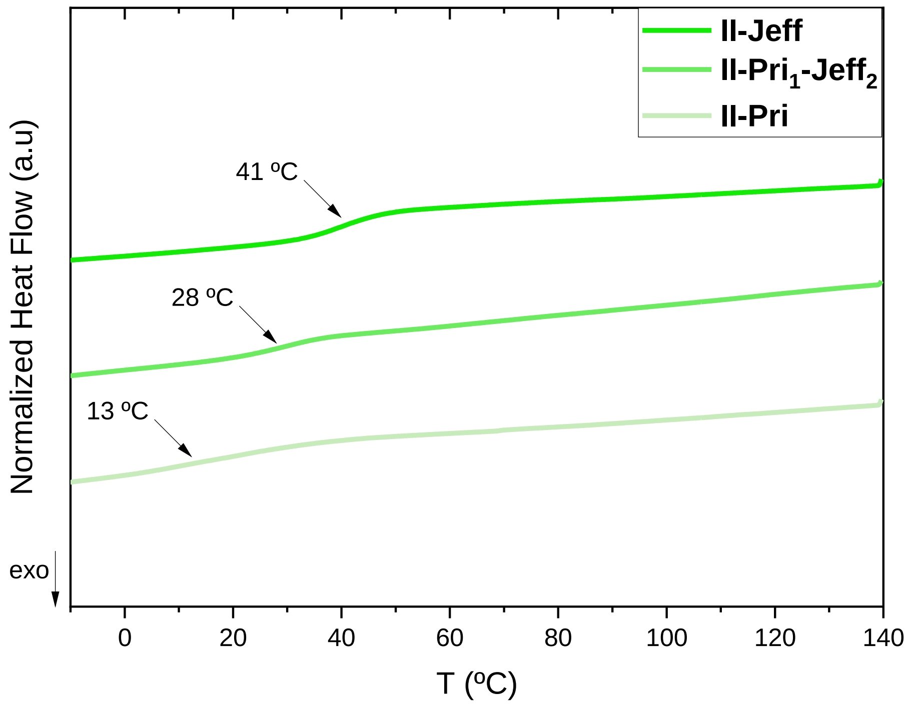

Figure S47. DSC curves (2nd heating scan) of **II-Pri**, **II-Pri1-Jeff2**, **II-Jeff**.

DMA analysis

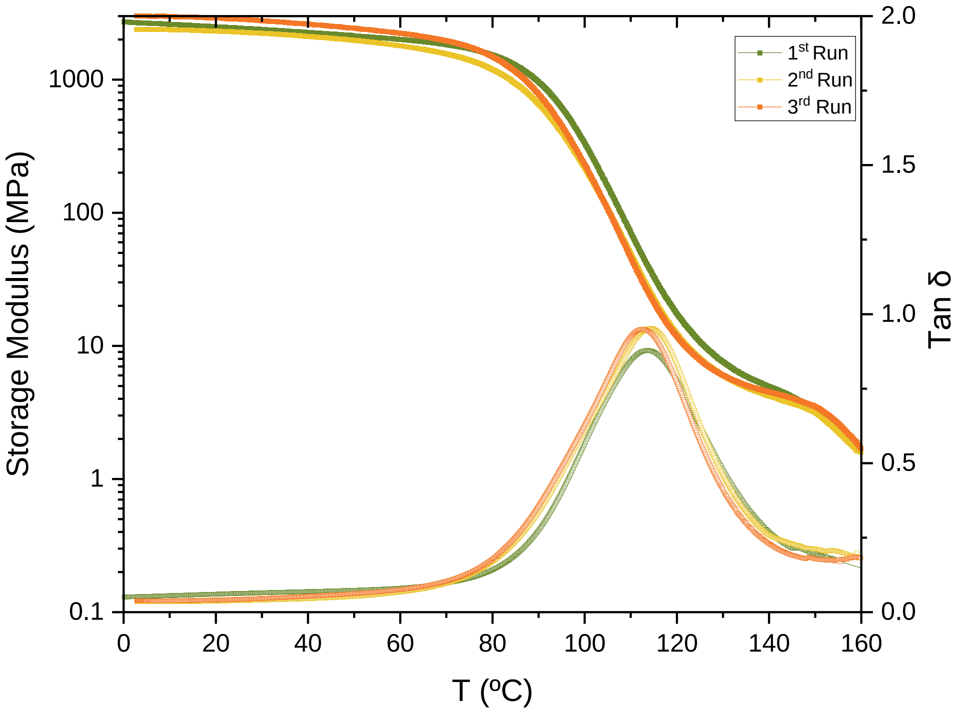

Figure S48. DMA of CAN **IS-TAEA**.

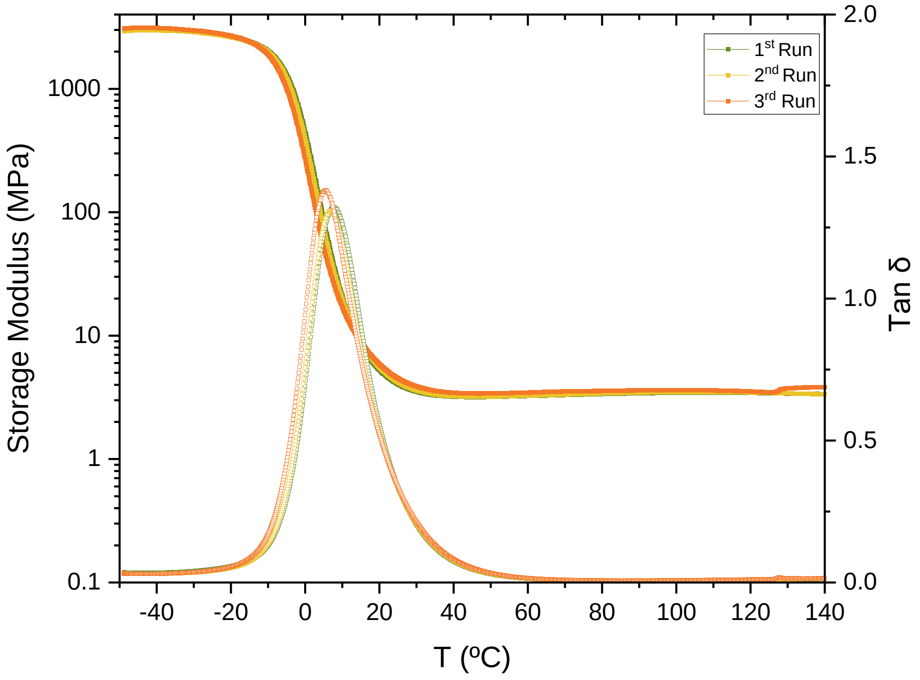

Figure S49. DMA of CAN **BD-Pri$_1$-Jeff$_2$**.

Stress relaxation experiments and Arrhenius obtained plots

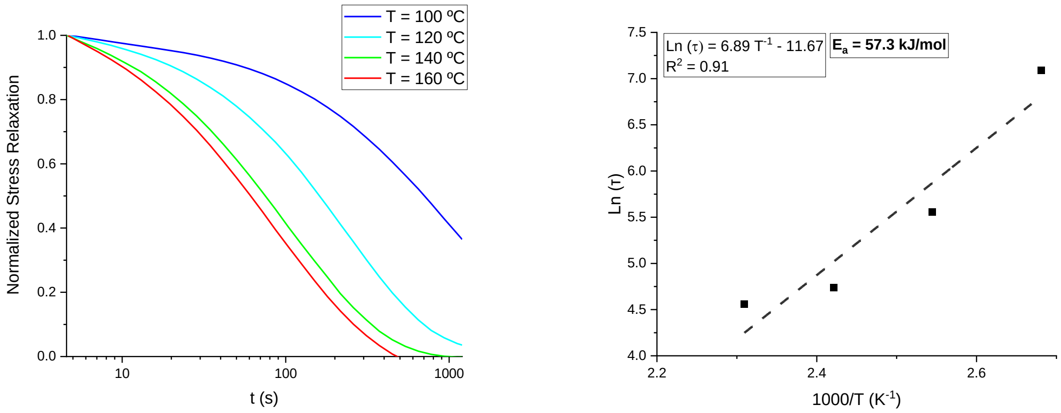

Figure S50. Stress relaxation curve of **IS-Pri** (*left*) and Arrhenius plot obtained from the relaxation times τ^* used to calculate the E_a of **IS-Pri** (*right*).

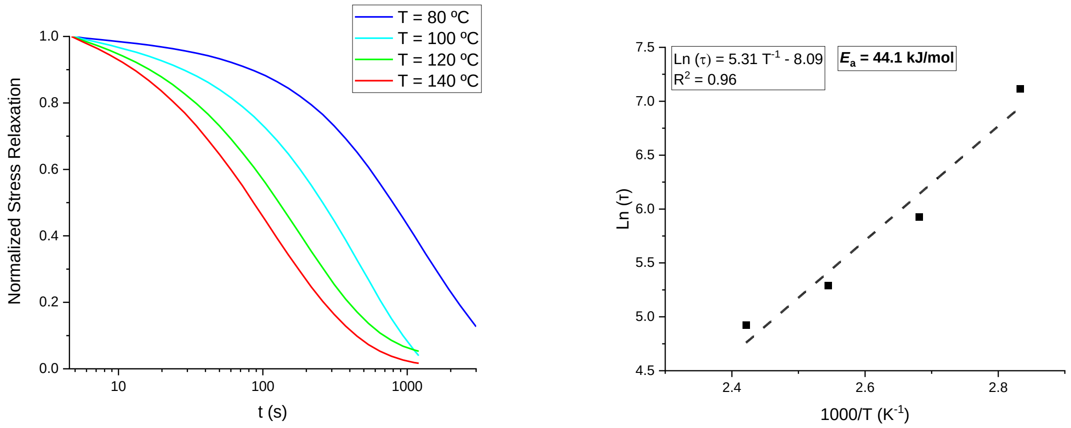

Figure S51. Stress relaxation curve of **IM-Pri** (*left*) and Arrhenius plot obtained from the relaxation times τ^* used to calculate the E_a of **IM-Pri** (*right*).

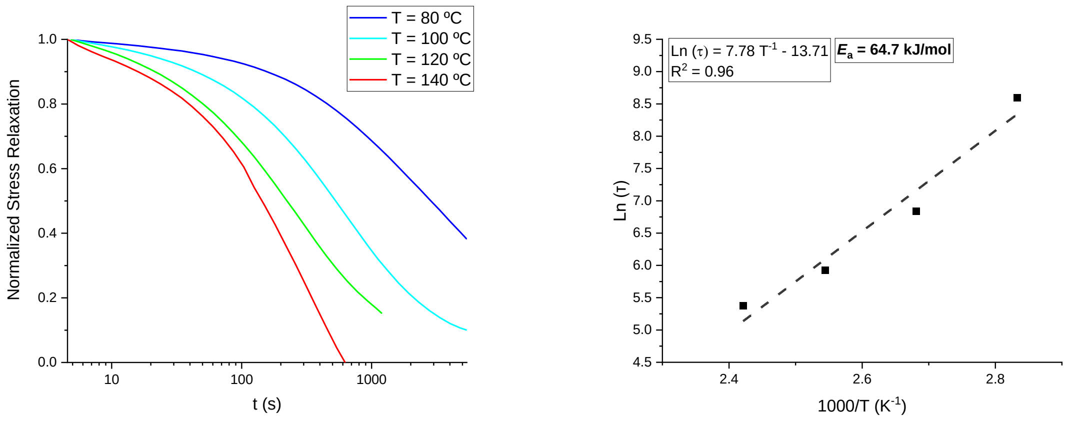

Figure S52. Stress relaxation curve of **II-Pri** (*left*) and Arrhenius plot obtained from the relaxation times τ^* used to calculate the E_a of **II-Pri** (*right*).

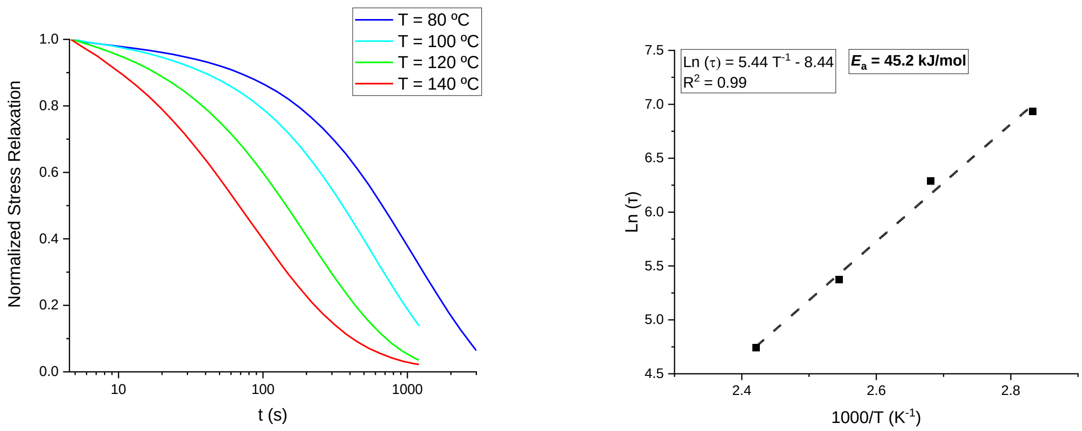

Figure S53. Stress relaxation curve of **IM$_{0.5}$-II$_{0.5}$-Pri** (*left*) and Arrhenius plot obtained from the relaxation times τ^* used to calculate the E_a of **IM$_{0.5}$-II$_{0.5}$-Pri** (*right*).

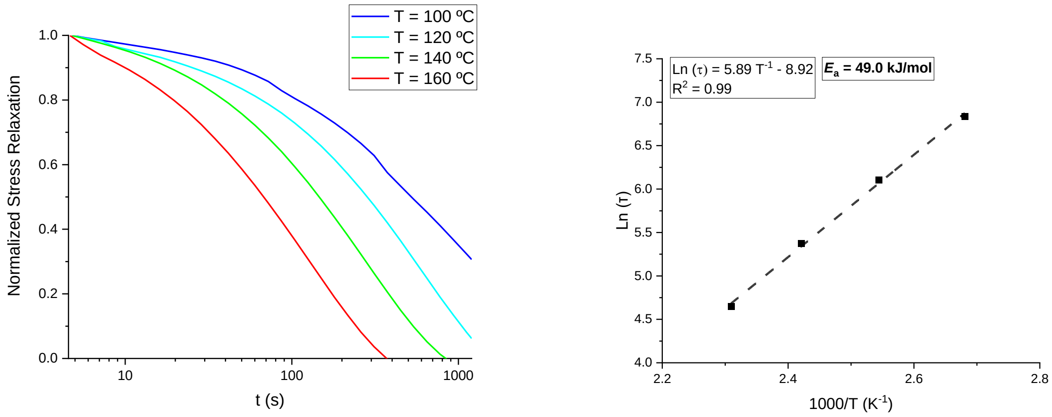

Figure S54. Stress relaxation curve of **IS-Pri1-Jeff2** (*left*) and Arrhenius plot obtained from the relaxation times τ^* used to calculate the E_a of **IS-Pri1-Jeff2** (*right*).

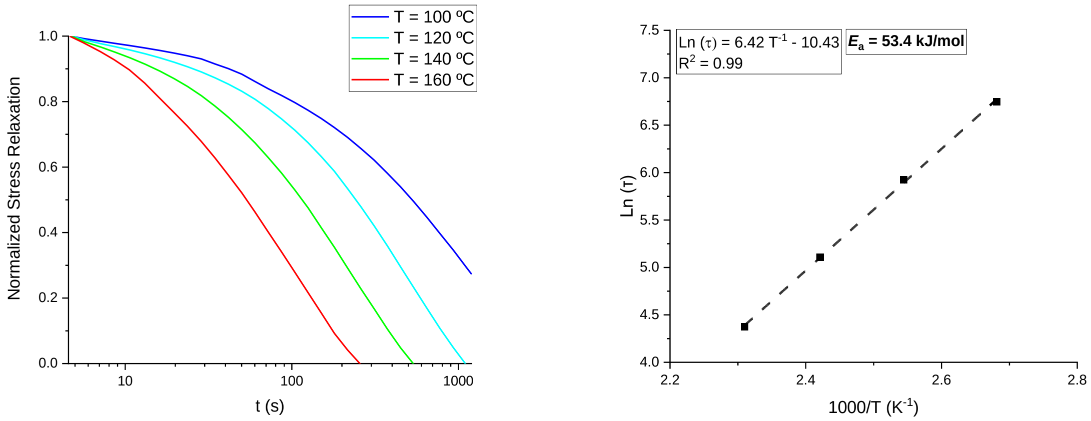

Figure S55. Stress relaxation curve of **IM-Pri1-Jeff2** (*left*) and Arrhenius plot obtained from the relaxation times τ^* used to calculate the E_a of **IM-Pri1-Jeff2** (*right*).

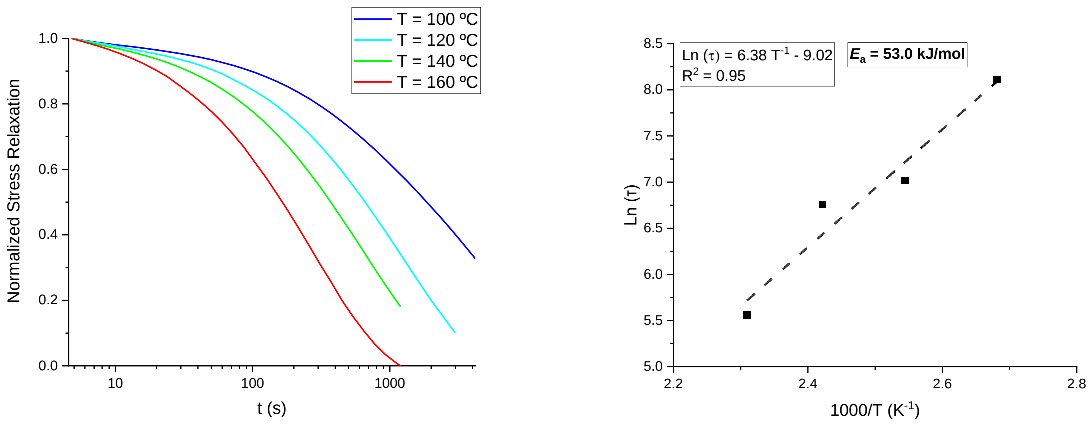

Figure S56. Stress relaxation curve of **II-Pri$_1$-Jeff$_2$** (*left*) and Arrhenius plot obtained from the relaxation times τ^* used to calculate the E_a of **II-Pri$_1$-Jeff$_2$** (*right*).

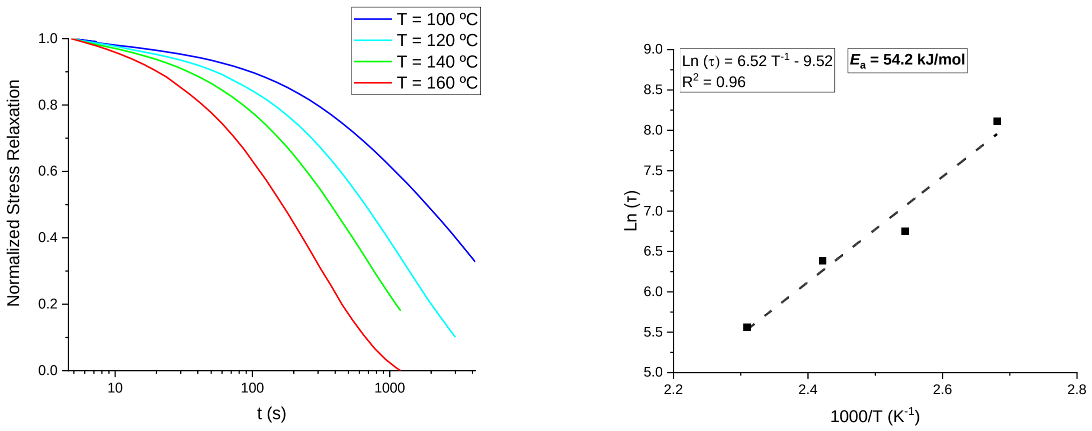

Figure S57. Stress relaxation curve of **IM$_{0.5}$-II$_{0.5}$-Pri$_1$-Jeff$_2$** (*left*) and Arrhenius plot obtained from the relaxation times τ^* used to calculate the E_a of **IM$_{0.5}$-II$_{0.5}$-Pri$_1$-Jeff$_2$** (*right*).

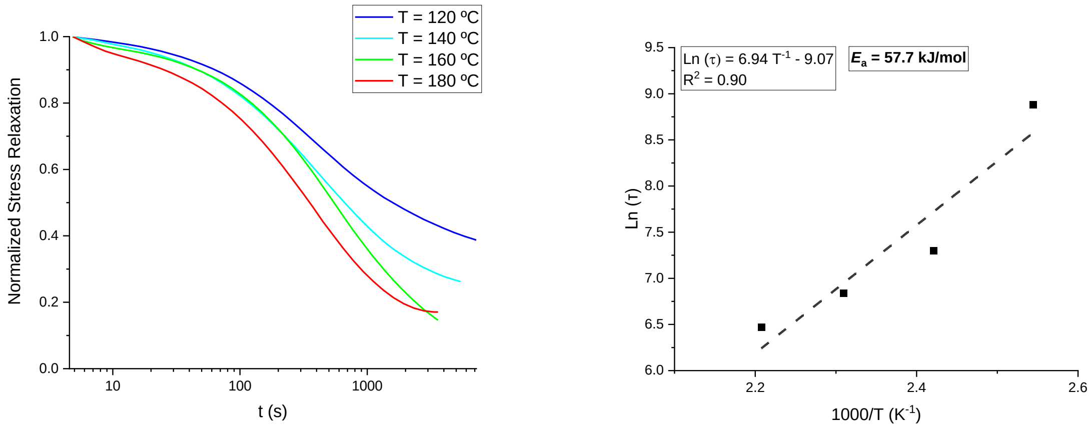

Figure S58. Stress relaxation curve of **IS-Jeff** (*left*) and Arrhenius plot obtained from the relaxation times τ^* used to calculate the E_a of **IS-Jeff** (*right*).

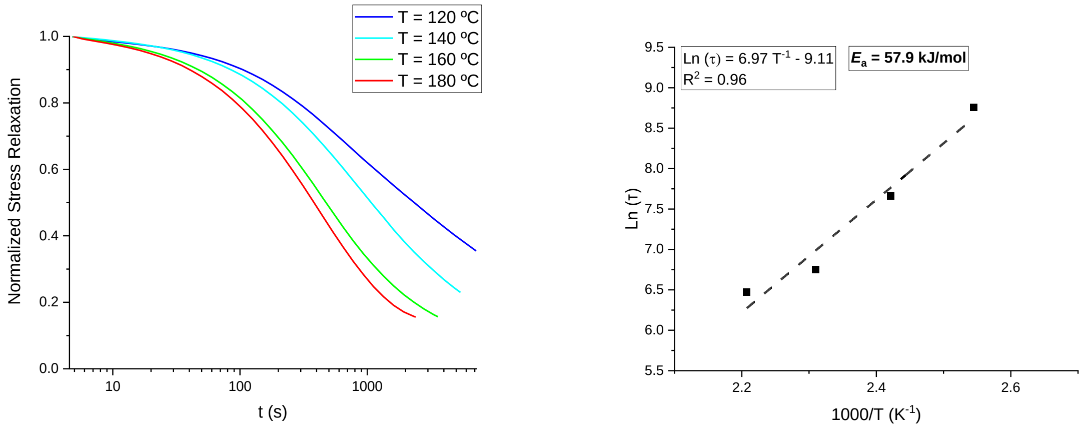

Figure S59. Stress relaxation curve of **IM-Jeff** (*left*) and Arrhenius plot obtained from the relaxation times τ^* used to calculate the E_a of **IM-Jeff** (*right*).

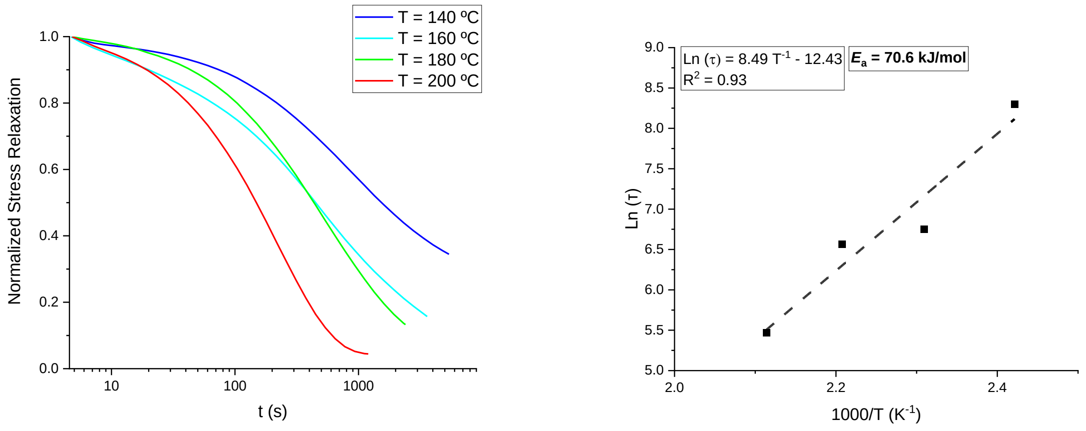

Figure S60. Stress relaxation curve of **II-Jeff** (*left*) and Arrhenius plot obtained from the relaxation times τ^* used to calculate the E_a of **II-Jeff** (*right*).

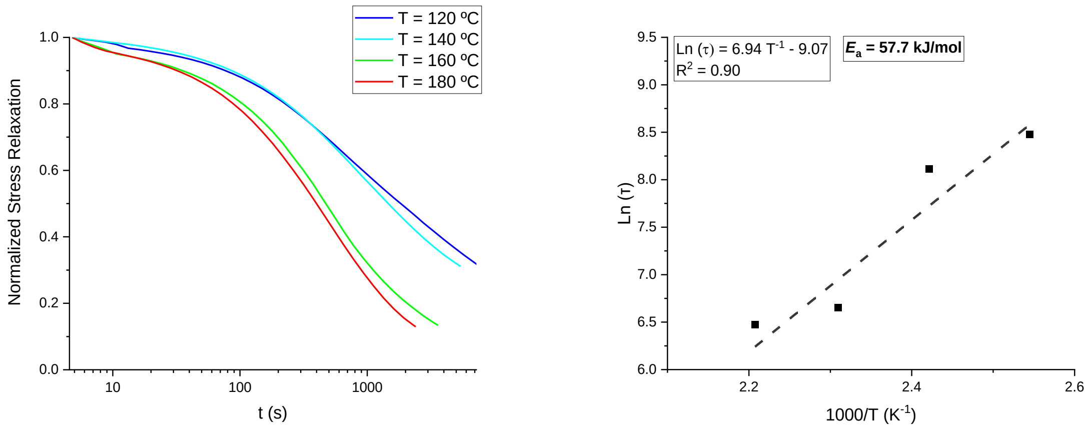

Figure S61. Stress relaxation curve of **IM$_{0.5}$-II$_{0.5}$-Jeff** (*left*) and Arrhenius plot obtained from the relaxation times τ^* used to calculate the E_a of **IM$_{0.5}$-II$_{0.5}$-Jeff** (*right*).

Molecular mechanics (MM2) calculation

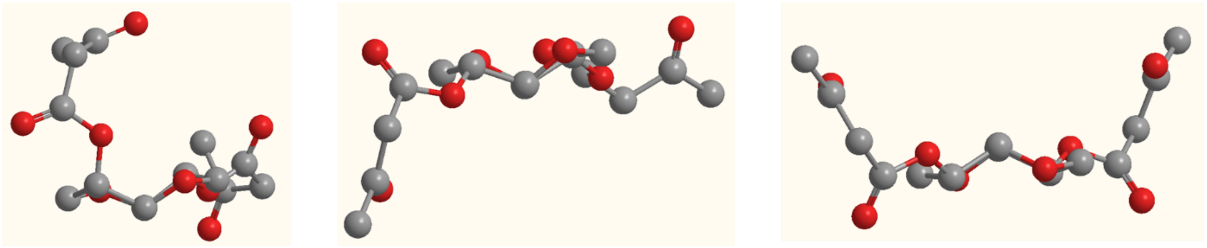

IS-AAc IM-AAc II-AAc

Figure S62. Ball and stick model for **IS-AAc**, **IM-AAc** and **II-AAc** using MM2 energy-minimized method. In grey and red carbon and oxygen atoms respectively. Hydrogen atoms were removed for simplicity.

Stress-strain curves

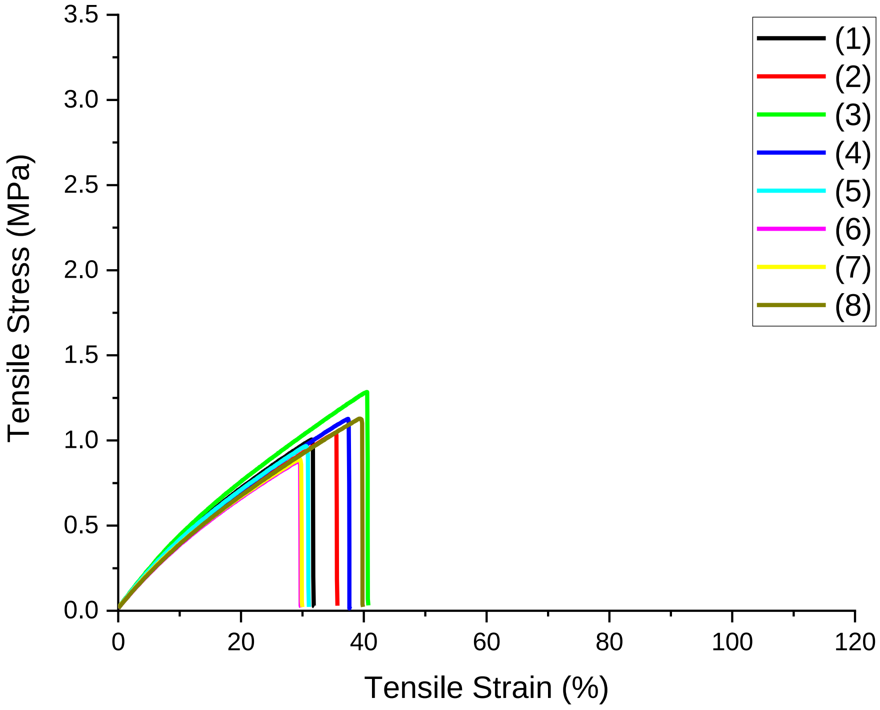

Figure S63. Stress-strain curve of **BD-Pri$_1$-Jeff$_2$**, numbers correspond to each sample run.

Calculations for stress relaxation derived from activation energy

- Equation obtained from Arrhenius law
  - **IS-Pri** ($Ln\ \tau^* = 6.89\ T^{-1} - 11.67$)
  - **IM-Pri** ($Ln\ \tau^* = 5.31\ T^{-1} - 8.09$)
  - **II-Pri** ($Ln\ \tau^* = 7.78\ T^{-1} - 13.71$)
  - **$IM_{0.5}$-$II_{0.5}$-Pri** ($Ln\ \tau^* = 5.44\ T^{-1} - 8.44$)
  - **IS-Pri_1-$Jeff_2$** ($Ln\ \tau^* = 5.89\ T^{-1} - 8.92$)
  - **IM-Pri_1-$Jeff_2$** ($Ln\ \tau^* = 6.42\ T^{-1} - 10.43$)
  - **II-Pri_1-$Jeff_2$** ($Ln\ \tau^* = 6.38\ T^{-1} - 9.02$)
  - **$IM_{0.5}$-$II_{0.5}$-Pri_1-$Jeff_2$** ($Ln\ \tau^* = 6.52\ T^{-1} - 9.52$)
  - **IS-Jeff** ($Ln\ \tau^* = 6.94\ T^{-1} - 9.07$)
  - **IM-Jeff** ($Ln\ \tau^* = 6.97\ T^{-1} - 9.11$)
  - **II-Jeff** ($Ln\ \tau^* = 8.49\ T^{-1} - 12.43$)
  - **$IM_{0.5}$-$II_{0.5}$-Jeff** ($Ln\ \tau^* = 6.66\ T^{-1} - 8.36$)
- Arrhenius law related to E_a

$$Ln\ \tau^* = \ \tau_0\ e^{\frac{E_a}{RT}} \quad (R = 8.314\ \frac{J}{mol\ K})$$

$$Ln\ \tau^* = Ln\ \tau_0 + \frac{E_a}{RT} \quad (R = 8.314\ \frac{J}{mol\ K})$$

  - **IS-Pri_1** ($E_a = 6.89 \times 1000 \times 8.314 = 57.3\ kJ/mol$)
  - **IM-Pri** ($E_a = 5.31 \times 1000 \times 8.314 = 44.1\ kJ/mol$)
  - **II-Pri** ($E_a = 7.78 \times 1000 \times 8.314 = 64.7\ kJ/mol$)
  - **$IM_{0.5}$-$II_{0.5}$-Pri** ($E_a = 5.44 \times 1000 \times 8.314 = 45.2\ kJ/mol$)
  - **IS-Pri_1-$Jeff_2$** ($E_a = 5.89 \times 1000 \times 8.314 = 49.0\ kJ/mol$)
  - **IM-Pri_1-$Jeff_2$** ($E_a = 6.42 \times 1000 \times 8.314 = 53.4\ kJ/mol$)
  - **II-Pri_1-$Jeff_2$** ($E_a = 6.38 \times 1000 \times 8.314 = 53.0\ kJ/mol$)
  - **$IM_{0.5}$-$II_{0.5}$-Pri_1-$Jeff_2$** ($E_a = 6.52 \times 1000 \times 8.314 = 54.2\ kJ/mol$)
  - **IS-Jeff** ($E_a = 6.94 \times 1000 \times 8.314 = 57.7\ kJ/mol$)
  - **IM-Jeff** ($E_a = 6.97 \times 1000 \times 8.314 = 57.9\ kJ/mol$)
  - **II-Jeff** ($E_a = 8.49\ \times 1000 \times 8.314 = 70.6\ kJ/mol$)
  - **$IM_{0.5}$-$II_{0.5}$-Jeff** ($E_a = 6.66\ \times 1000 \times 8.314 = 55.4\ kJ/mol$)

Calculations for vitrimer temperature (T_v) using Arrhenius equation from stress relaxation

- Equation obtained from Arrhenius law
  - **IS-Pri** ($Ln\,\tau^* = 6.89\,T^{-1} - 11.67$)
  - **IM-Pri** ($Ln\,\tau^* = 5.31\,T^{-1} - 8.09$)
  - **II-Pri** ($Ln\,\tau^* = 7.78\,T^{-1} - 13.71$)
  - **IM$_{0.5}$-II$_{0.5}$-Pri** ($Ln\,\tau^* = 5.44\,T^{-1} - 8.44$)
  - **IS-Pri$_1$-Jeff$_2$** ($Ln\,\tau^* = 5.89\,T^{-1} - 8.92$)
  - **IM-Pri$_1$-Jeff$_2$** ($Ln\,\tau^* = 6.42\,T^{-1} - 10.43$)
  - **II-Pri$_1$-Jeff$_2$** ($Ln\,\tau^* = 6.38\,T^{-1} - 9.02$)
  - **IM$_{0.5}$-II$_{0.5}$-Pri$_1$-Jeff$_2$** ($Ln\,\tau^* = 6.52\,T^{-1} - 9.52$)
  - **IS-Jeff** ($Ln\,\tau^* = 6.94\,T^{-1} - 9.07$)
  - **IM-Jeff** ($Ln\,\tau^* = 6.97\,T^{-1} - 9.11$)
  - **II-Jeff** ($Ln\,\tau^* = 8.49\,T^{-1} - 12.43$)
  - **IM$_{0.5}$-II$_{0.5}$-Jeff** ($Ln\,\tau^* = 6.66\,T^{-1} - 8.36$)

- Maxwell equation is used to calculate T_v, using a viscosity value (η = 10^{12} Pa·s).

$$\eta = \frac{E' \times \tau^*}{3}$$

$$\tau^* = \frac{3 \times \eta}{E'}$$

  - **IS-Pri** ($E' = 1.15\,MPa$), ($\tau^* = 2613194.9\,MPa$), ($Ln\,\tau^* = 14.78$)
  - **IM-Pri** ($E' = 1.44\,MPa$), ($\tau^* = 2079617.0\,MPa$), ($Ln\,\tau^* = 14.55$)
  - **II-Pri** ($E' = 1.43\,MPa$), ($\tau^* = 2096250.5\,MPa$), ($Ln\,\tau^* = 14.56$)
  - **IM$_{0.5}$-II$_{0.5}$-Pri** ($E' = 1.91\,MPa$), ($\tau^* = 1567602.9\,MPa$), ($Ln\,\tau^* = 14.27$)
  - **IS-Pri$_1$-Jeff$_2$** ($E' = 2.05\,MPa$), ($\tau^* = 1460062.4\,MPa$), ($Ln\,\tau^* = 14.19$)
  - **IM-Pri$_1$-Jeff$_2$** ($E' = 1.71\,MPa$), ($\tau^* = 1751098.8\,MPa$), ($Ln\,\tau^* = 14.38$)
  - **II-Pri$_1$-Jeff$_2$** ($E' = 1.45\,MPa$), ($\tau^* = 2072381.4\,MPa$), ($Ln\,\tau^* = 14.54$)
  - **IM$_{0.5}$-II$_{0.5}$-Pri$_1$-Jeff$_2$** ($E' = 2.21\,MPa$), ($\tau^* = 1357026.0\,MPa$), ($Ln\,\tau^* = 14.12$)
  - **IS-Jeff** ($E' = 3.26\,MPa$), ($\tau^* = 919172.1\,MPa$), ($Ln\,\tau^* = 13.73$)
  - **IM-Jeff** ($E' = 2.50\,MPa$), ($\tau^* = 1200036.8\,MPa$), ($Ln\,\tau^* = 14.00$)

- **II-Jeff** $(E' = 3.17\ MPa)$, $(\tau^* = 945095.2\ MPa)$, $(Ln\,\tau^* = 13.76)$
- **IM0.5-II0.5-Jeff** $(E' = 3.21\ MPa)$, $(\tau^* = 934524.1\ MPa)$, $(Ln\,\tau^* = 13.75)$

- Stress relaxation equation is used to calculate T_v

IS-Pri

$$x = \frac{1000}{T} = \frac{Ln\,\tau^* + 11.67}{6.89} = 3.84$$

$$T_v = \frac{1000}{x} = \frac{1000}{3.84} = 260\ K = -12\ °C$$

IM-Pri

$$x = \frac{1000}{T} = \frac{Ln\,\tau^* + 8.09}{5.31} = 4.26$$

$$T_v = \frac{1000}{x} = \frac{1000}{4.26} = 235\ K = -38\ °C$$

II-Pri

$$x = \frac{1000}{T} = \frac{Ln\,\tau^* + 13.71}{7.78} = 3.63$$

$$T_v = \frac{1000}{x} = \frac{1000}{3.63} = 275\ K = 2\ °C$$

IM0.5-II0.5-Pri

$$x = \frac{1000}{T} = \frac{Ln\,\tau^* + 8.44}{5.44} = 4.17$$

$$T_v = \frac{1000}{x} = \frac{1000}{4.17} = 240\ K = -33\ °C$$

IS-Pri1-Jeff2

$$x = \frac{1000}{T} = \frac{Ln\,\tau^* + 8.92}{5.89} = 3.92$$

$$T_v = \frac{1000}{x} = \frac{1000}{3.92} = 255\ K = -18\ °C$$

IM-Pri1-Jeff2

$$x = \frac{1000}{T} = \frac{Ln\,\tau^* + 10.43}{6.42} = 3.86$$

$$T_v = \frac{1000}{x} = \frac{1000}{3.86} = 259\ K = \ -14\ °C$$

II-Pri1-Jeff2

$$x = \frac{1000}{T} = \frac{Ln\ \tau^* + 9.02}{6.38} = 3.69$$

$$T_v = \frac{1000}{x} = \frac{1000}{3.69} = 271\ K = \ -2\ °C$$

IM0.5-II0.5-Pri1-Jeff2

$$x = \frac{1000}{T} = \frac{Ln\ \tau^* + 9.52}{6.52} = 3.63$$

$$T_v = \frac{1000}{x} = \frac{1000}{3.63} = 275\ K = \ 2\ °C$$

IS-Jeff

$$x = \frac{1000}{T} = \frac{Ln\ \tau^* + 9.07}{6.94} = 3.29$$

$$T_v = \frac{1000}{x} = \frac{1000}{3.29} = 304\ K = \ 31\ °C$$

IM-Jeff

$$x = \frac{1000}{T} = \frac{Ln\ \tau^* + 9.11}{6.97} = 3.32$$

$$T_v = \frac{1000}{x} = \frac{1000}{3.32} = 302\ K = \ 29\ °C$$

II-Jeff

$$x = \frac{1000}{T} = \frac{Ln\ \tau^* + 12.43}{8.49} = 3.08$$

$$T_v = \frac{1000}{x} = \frac{1000}{3.08} = 324\ K = \ 51\ °C$$

IM0.5-II0.5-Jeff

$$x = \frac{1000}{T} = \frac{Ln\ \tau^* + 8.36}{6.66} = 3.32$$

$$T_v = \frac{1000}{x} = \frac{1000}{3.32} = 301\ K = \ 28\ °C$$

Mechanical properties of vinylogous urethane CANs

Table S2. Mechanical properties of vinylogous urethane CANs.

Entry	CAN	Reprocessing Cycle (RC)	*E* (MPa)	σ_b (MPa)	ε_b (%)
1	IS-Pri	Pristine	10.1 ± 0.50	1.86 ± 0.11	77.1 ± 3.2
2	IS-Pri	1st	13.2 ± 0.69	1.79 ± 0.16	70.5 ± 6.6
3	IS-Pri	2nd	10.4 ± 0.78	1.57 ± 0.16	64.1 ± 4.9
4	IS-Pri	3rd	14.7 ± 4.5	0.79 ± 0.21	23.5 ± 3.3
5	IM-Pri	Pristine	12.8 ± 5.8	2.54 ± 0.30	70.1 ± 4.0
6	II-Pri	Pristine	27.2 ± 8.0	2.55 ± 0.67	45.5 ± 6.5
7	$II_{0.5}$-$IM_{0.5}$-Pri_1	Pristine	25.4 ± 1.3	2.98 ± 0.43	60.3 ± 7.8
8	IS-Pri_1-$Jeff_2$	Pristine	198.9 ± 52.0	9.20 ± 0.73	83.5 ± 11.8
9	IS-Pri_1-$Jeff_2$	1st	167.0 ± 46.3	9.21 ± 1.94	88.0 ± 15.5
10	IS-Pri_1-$Jeff_2$	2nd	83.7 ± 5.4	8.31 ± 0.99	107.6 ± 8.5
11	IS-Pri_1-$Jeff_2$	3rd	120.0 ± 30.9	6.79 ± 0.85	82.2 ± 11.1
12	IM-Pri_1-$Jeff_2$	Pristine	71.5 ± 48.3	6.56 ± 0.51	129.2 ± 4.5
13	II-Pri_1-$Jeff_2$	Pristine	380.9 ± 28.4	7.00 ± 0.52	52.7 ± 10.6
14	$II_{0.5}$-$IM_{0.5}$-Pri_1-$Jeff_2$	Pristine	266.7 ± 48.3	8.32 ± 0.53	71.6 ± 3.0
15	IS-Jeff	Pristine	2102 ± 93	18.0 ± 2.4	1.52 ± 0.28
16	IM-Jeff	Pristine	2041 ± 69	19.1 ± 2.7	1.57 ± 0.21
17	II-Jeff	Pristine	1870 ± 229	16.8 ± 2.3	1.63 ± 0.08
18	$II_{0.5}$-$IM_{0.5}$-Jeff	Pristine	1911 ± 144	16.8 ± 1.3	1.45 ± 0.13
19	BD-Pri_1-$Jeff_2$	Pristine	4.74 ± 0.27	1.04 ± 0.14	34.3 ± 4.5

Chemical degradation

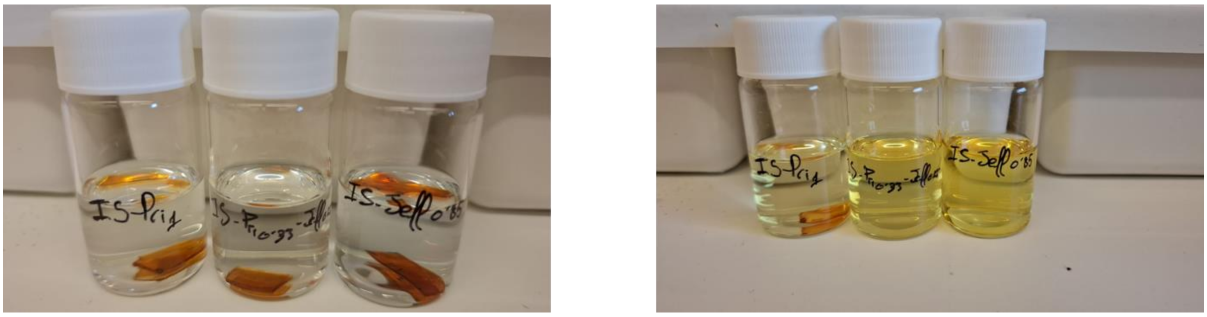

Figure S64. IS-Pri$_x$-Jeff$_y$ CANs in EtOH (*left*) and degraded **IS-Pri$_x$-Jeff$_y$** after addition of benzyl amine and orbital stirring at room temperature for 24 h (*right*).

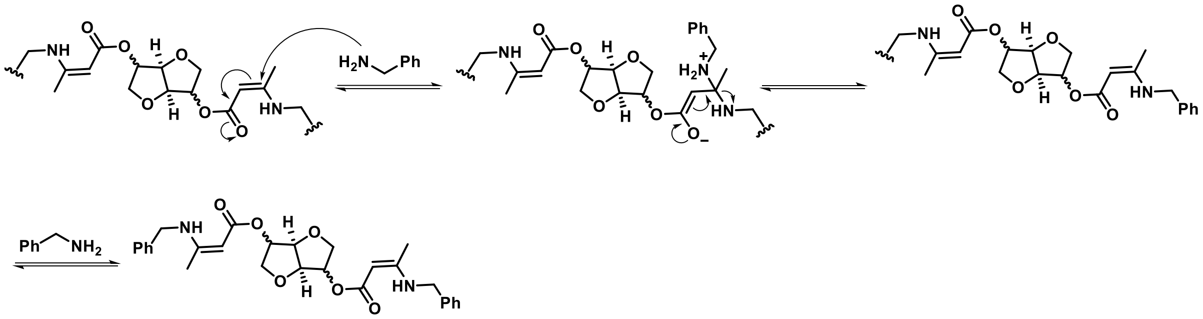

Scheme S6. Mechanism for the chemical degradation of CANs.

References

(1) Stubbs, C. J.; Worch, J. C.; Prydderch, H.; Wang, Z.; Mathers, R. T.; Dobrynin, A. V.; Becker, M. L.; Dove, A. P. Sugar-Based Polymers with Stereochemistry-Dependent Degradability and Mechanical Properties. *J. Am. Chem. Soc.* **2022**, *144* (3), 1243-1250.
